# Regulation of cashmere fineness traits by noncoding RNA in Jiangnan cashmere goats

**DOI:** 10.1186/s12864-023-09531-x

**Published:** 2023-10-11

**Authors:** Cuiling Wu, Qin Xu, Jianying Li, Chongkai Qin, Hanikezi Tulafu, Wenna Liu, Qingwei Lu, Wenxin Zheng, Xuefeng Fu

**Affiliations:** 1 School of Life Sciences, Xinjiang Normal University, Urumqi, China; 2 Key Laboratory of Special Environmental Medicine, Xinjiang Military General Hospital, Urumqi, China; 3 Aksu Prefecture Animal Husbandry Technology Extension Center, Aksu, China; 4 Key Laboratory of Genetics Breeding and Reproduction of Xinjiang Wool-sheep & Cashmere-goat (XJYS1105), Institute of Animal Science, Xinjiang Academy of Animal Sciences, Urumqi, China; 5 College of Animal Science, Xinjiang Agricultural University, Urumqi, China; 6 Xinjiang Uygur Autonomous Region Breeding sheep and wool Cashmere Quality Safety Supervision and Inspection Center, Institute of Animal Husbandry Quality Standard, Xinjiang Academy of Animal Sciences, Urumqi, Xinjiang China

**Keywords:** Transcriptomic, ncRNA, Jiangnan cashmere goat, Cashmere fineness

## Abstract

**Background:**

Cashmere has long been used as the raw material for wool textiles. The diameter of the cashmere fibre determines its quality and economic value. However, the regulatory role of noncoding RNAs (ncRNAs) in cashmere fineness remains unclear, especially regarding the interaction between ncRNAs and coding RNAs.

**Results:**

Transcriptome sequencing was used to identify the expression profiles of long noncoding RNAs (lncRNAs), circular RNAs (circRNAs) and microRNAs (miRNAs) in the skin tissues of Jiangnan cashmere goats with different cashmere fineness levels. Integration analysis of ncRNA and coding RNA was performed in combination with previous research results. The results showed that 16,437 lncRNAs, 2234 circRNAs, and 1322 miRNAs were identified in 8 skin samples of cashmere goats. A total of 403 differentially expressed (DE) lncRNAs, 62 DE circRNAs and 30 DE miRNAs were identified in the skin tissues of the fine groups (Fe) and coarse groups (Ce). We predicted the target gene of DE lncRNA, the target gene of DE miRNA and the host gene of DE circRNA. Based on functional annotation and enrichment analysis of target genes, we found that DE lncRNAs could be involved in regulating the fineness traits of cashmere. The most potential lncRNAs were MSTRG.42054.1, MSTRG.18602.3, and MSTRG.2199.13.

**Conclusions:**

The data from this study enriched the cashmere goat noncoding RNA database and helped to supplement the annotation of the goat genome. The results provided a new direction for the breeding of cashmere characters.

**Supplementary Information:**

The online version contains supplementary material available at 10.1186/s12864-023-09531-x.

## Background

Cashmere goats have two types of hair follicles in their skin. One is the primary hair follicle, which grows wool. The other is the secondary hair follicles, which produces cashmere that are high-quality natural textile materials. According to the textile processing method and the preferences of consumers, finer cashmere of the same colour has a higher price. In addition to the ecological environment and breeding management, the regulation of cashmere traits is mainly influenced by genetic factors. Therefore, it is important to find the key genes that influence the diameter of cashmere. Based on our previous work, the RNA expression levels of skin samples of cashmere with different fineness levels from Jiangnan cashmere goats [1] and Tibetan cashmere goats [2] were analysed by transcriptome sequencing technology. A number of candidate genes related to cashmere traits were screened, including *FA2H*, *SOX18*, *SOX4*, *WNT5A*, *NOTCH2*, *NOTCH3*, *KRT26*, *KRT28*, and *KRT39*. Similar studies have also been carried out on Liaoning cashmere goats and Inner Mongolian cashmere goats [3–5]. All these studies provided valuable genetic resources for cashmere goat genetic breeding and reproduction. It is generally believed that proteins are the final product of genetic information and are responsible gene functions. In fact, less than 2% of genes encode proteins, and the remaining noncoding transcripts are collectively referred to as ncRNA [6, 7]. With the development of science and technology, more studies have suggested that ncRNA is involved in physiological and developmental processes and the expression regulation of related genes in different ways [8, 9].

LncRNAs are noncoding RNA molecules with a length of more than 200 bp and a conserved secondary structure [10, 11]. LncRNAs regulate gene expression through various interaction mechanisms, such as cell cycle regulation, splicing regulation, gene imprinting, mRNA degradation and translation regulation [12]. MiRNA is a kind of noncoding single-stranded small molecule RNA with a length of 19–25 nt. MiRNA is produced by Dicer enzyme processing of a 70 nt single-stranded RNA precursor with a hairpin structure [13]. Similar to small interfering RNAs, miRNAs play an important regulatory role by specifically binding to target gene binding sites, inhibiting target gene translation or degrading target mRNA [14]. At present, in addition to lncRNAs and miRNAs, circRNAs have also attracted the attention of many scholars. CircRNA is a noncoding RNA with a covalent bond forming a ring structure [13]. More studies are needed on the biogenesis and characteristics of circRNAs to reveal the potential role of circRNAs in key processes in the organisms.

Currently, many studies have indicated that ncRNAs can participate in the regulation of hair growth in mammals. An earlier study showed that miR-125b was expressed in large quantities in skin stem cells, and the epidermis of transgenic mice overexpressing miR-125b became thicker, the sebaceous glands became larger, and the mice could not form a coat [15]. Other studies have shown that miR-200b and miR-196a are involved in controlling the development of mouse hair follicles through potential target genes in the WNT signalling pathway [16]. In several critical periods of sheep and goat fetuses, some lncRNAs, miRNAs and circRNAs showed significantly different expression trends, suggesting that they were involved in the regulation of hair growth and follicle development [17–19]. At present, ncRNA has also been studied in the regulation of hair follicle development and cashmere growth in several cashmere goat breeds, including Liaoning cashmere goat, Inner Mongolian cashmere goat and Shanbei white cashmere goat [5, 20–22]. However, the regulatory mechanism of ncRNA is complex. Analysis of ncRNAs combined with coding RNA and verification in different species will help clarify the regulatory mechanism of ncRNAs. In this study, we detected the expression of ncRNA in the skin samples of cashmere with different fineness levels form Jiangnan cashmere goats, and combined the results with those of previous work to explore the regulatory effect of ncRNA on cashmere growth [1]. This study aims to provide valuable genetic resources for the genetic improvement of cashmere goats.

## Results

### Quality control of high-throughput sequencing date

We sequenced eight chain specific libraries and obtained clean data of 92.68 Gb. The Q30 of each sample was no less than 95.03%. The ratio of clean reads from a single library to the reference genome ranged from 94.19 to 94.86% (Table S1). A total of 122.81 M clean reads were obtained by sequencing 8 small RNA libraries. The clean reads from each library were 12.56 M, and the Q30 was no less than 96.22%. The ratio of clean reads from a single library to the reference genome ranged from 78.68 to 83.76% (Table S2). The above data indicated that the noncoding RNA sequencing data were of high quality and could be used for subsequent analysis.

### Analysis of ncRNA expression level

In lncRNA analysis, the intersection of CNCI, CPAT, CPC and Pfam prediction results was used to identify 34,979 noncoding RNAs in 8 samples, including 16,437 lincRNAs, 2146 Antisense-lncRNAs, 15,513 Intronic-lncRNAs, 883 sense_lncRNAs (Fig. S1A-B). A total of 2234 circRNAs were identified in 8 skin tissues. The distribution of circRNA reads on different chromosomes was significantly different (Fig. S1C). CircRNA lengths were mainly concentrated in the range of 400–1200 nt. In addition, most circRNAs were derived from the exon region of the source gene, and only a few were derived from the intergenic region and intron region (Fig. S1D). The above characteristics are consistent with mammalian circRNA characteristics. A total of 1,322 miRNAs were identified from 8 small RNA libraries, including 422 known miRNAs and 900 novel miRNAs.

**Fig. 1:**
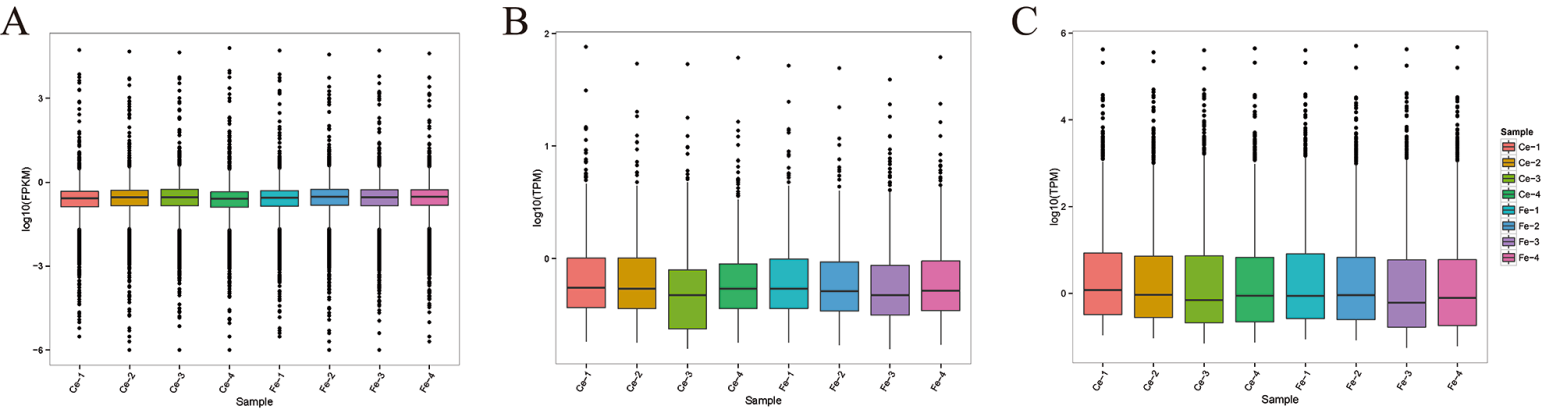
Boxplot of noncoding RNA expression levels. (**A**) LncRNA. (**B**) CircRNA. (**C**) MiRNA. The ordinate represents the log value of the sample expression quantity (FPKM/TPM).

The boxplot of ncRNA expression distribution is shown in Fig. 1. The FPKM distribution for lncRNAs is shown in Fig. 1A, and the TPM distribution for circRNAs and miRNAs is shown in Fig. 1B, C. The overall expression levels of lncRNAs, circRNAs and miRNAs were low in the skin tissues of 8 Jiangnan cashmere goats. lncRNA expression levels in different samples were concentrated, and the degree of fluctuation was small. The expression levels of circRNA and miRNA fluctuated greatly.

### Differential expression analysis of ncRNA

**Fig. 2:**
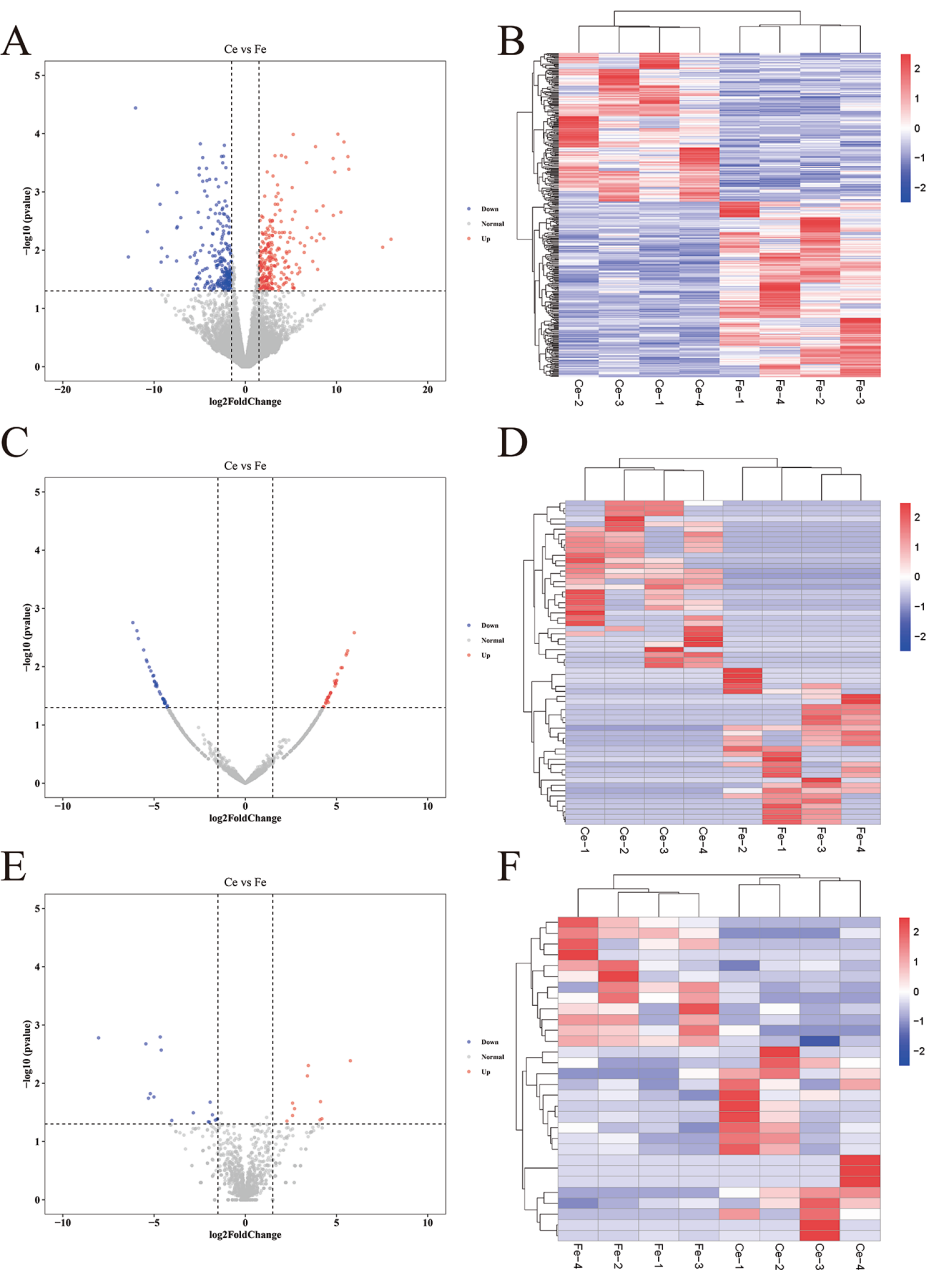
Volcano plots and heatmaps of DE ncRNAs. (**A, B**) DE LncRNA analysis. (**C, D**) DE circRNA analysis. (**E, F**) DE miRNA analysis

When the fold change was ≥ 2 and the P value was ≤ 0.05, 403 DE lncRNAs were identified in skin samples of the Ce groups and Fe groups, among which 219 lncRNAs were upregulated and 184 were downregulated in the Fe group (Fig. 2A, B). Sixty-two DE circRNAs were identified in the samples of the Ce and Fe groups, with 30 circRNAs upregulated and 32 circRNAs downregulated in the Fe group (Fig. 2C, D). Similarly, 30 DE miRNAs were identified in the different groups. The expression of 12 and 8 DE miRNAs was upregulated and downregulated in the Fe group, respectively (Fig. 2E, F).

### Functional annotation of DE lncRNAs

**Fig. 3:**
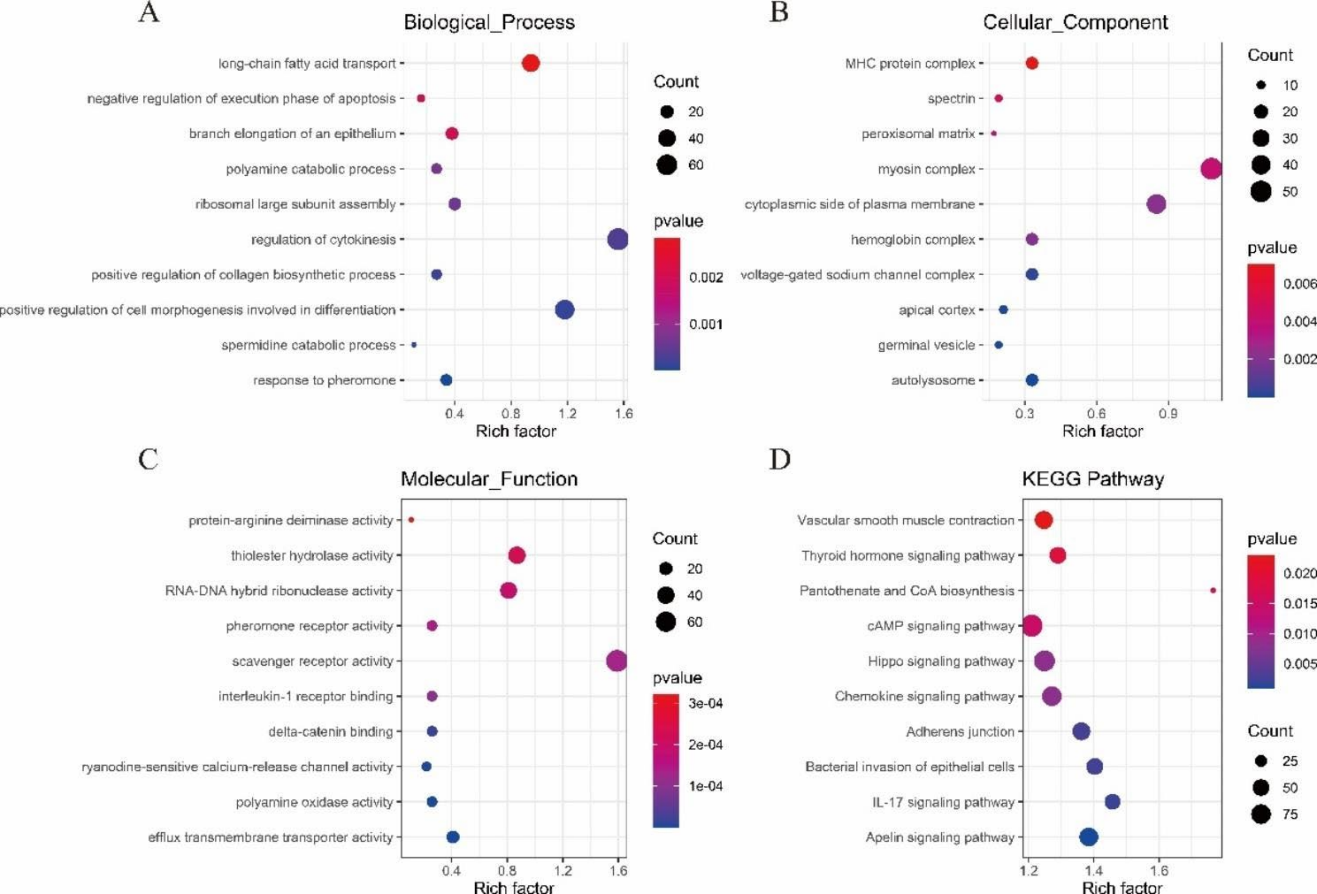
Functional annotation of DE lncRNA target genes. (**A**) Biological process. (**B**) Cellular component. (**C**) Molecular function. (**D**) KEGG pathways

The 12,049 target genes were predicted for 403 DE lncRNAs, with 10,861 target genes predicted in trans, and 1,907 target genes predicted in cis. It is worth noting that 28 genes were predicted in both trans and cis. We performed functional annotation and enrichment analysis of DE lncRNA target genes. The top 10 GO terms and pathways with the highest enrichment were plotted as bubble maps (P < 0.05). Based on GO analysis, in the biological processes (BP) category, target genes were mainly enriched in the positive regulation of collagen biosynthetic process (GO:0032967), branch elongation of an epithelium (GO:1,900,118), and negative regulation of execution phase of apoptosis (GO:0015909) (Fig. 3A). In the classification of cellular component (CC) category, the target genes were mainly enriched in hemoglobin by autolysosome (GO:0044754), hemoglobin by hemoglobin complex (GO:0005833), hemoglobin by hemoglobin by autolysosome (GO:0044754), hemoglobin complex (GO:0005833), and MHC protein complex (GO:0042611) (Fig. 3B). In the molecular function (MF) category, target genes were mainly enriched in efflux transmembrane transporter activity (GO:0015562), pheromone receptor activity (GO:0016503), and thiolester hydrolase activity (GO:0016790) (Fig. 3C). Similarly, KEGG annotation analysis showed that DE lncRNA target genes were mainly enriched in bacterial invasion of epithelial cells (ko05100), pantothenate and CoA biosynthesis (ko00770), and vascular smooth muscle contraction(ko04270) (Fig. 3D).

### Functional annotation of DE circRNA host genes

**Fig. 4:**
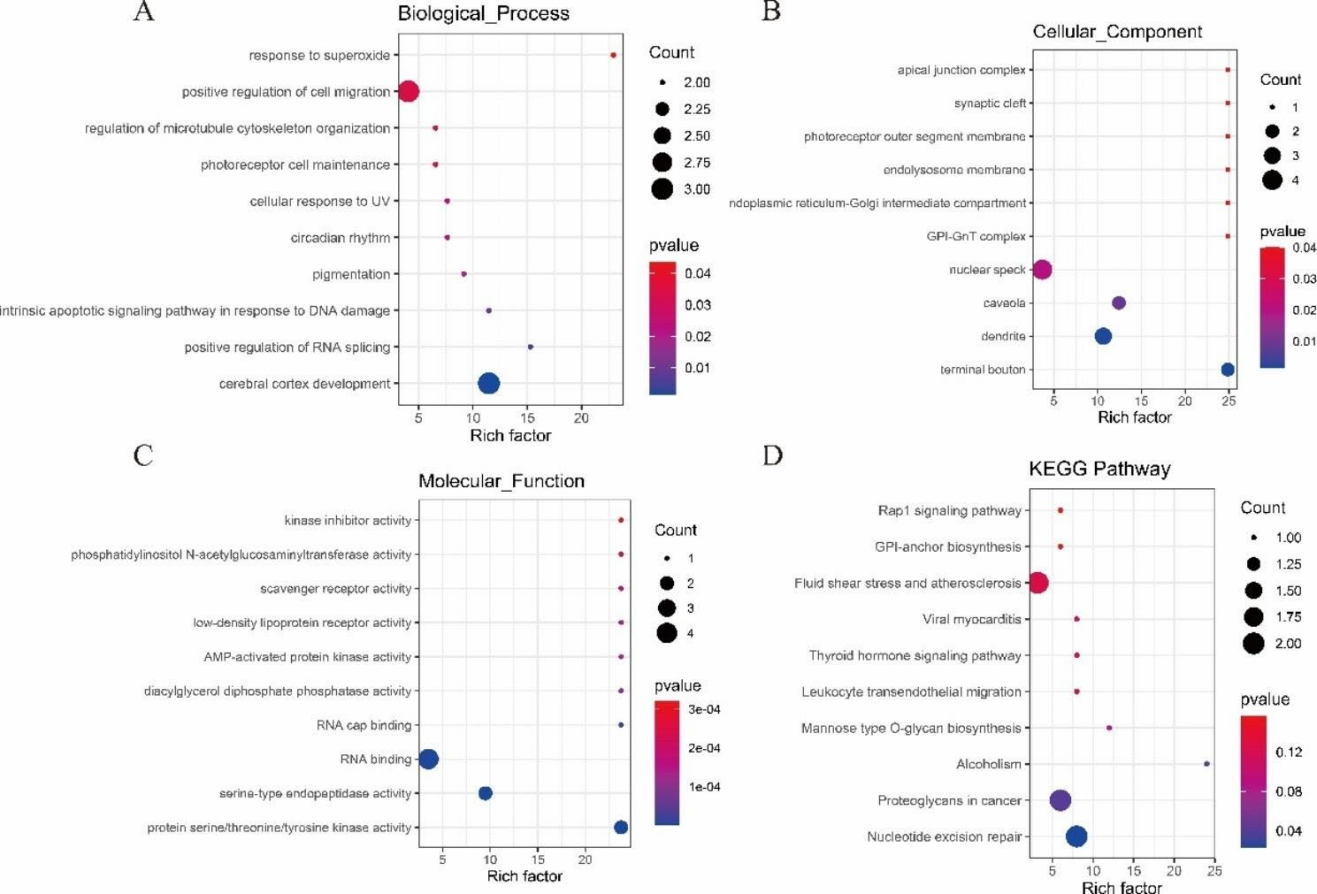
Functional annotation of DE circRNA target genes. (**A**) Biological process. (**B**) Cellular component. (**C**) Molecular function. (**D**). KEGG pathways

The 62 DE circRNAs were derived from 61 genes. Based on GO analysis, in the BP category, DE circRNA host genes were mainly enriched in cerebral cortex development (GO:0021987), positive regulation of RNA splicing (GO:0033120), and pigmentation (GO:0043473) (Fig. 4A). In the CC category, host genes were mainly enriched in terminal bouton (GO:0043195), dendrite (GO:0030425), and caveola (GO:0005901) (Fig. 4B). In the MF category, the host genes were mainly enriched in serine-type endopeptidase activity (GO:0004252), RNA binding (GO:0003723), and RNA cap binding (GO:0000339) (Fig. 4C). Similarly, KEGG annotation analysis showed that host genes were mainly enriched in nucleotide excision repair (ko03420), proteoglycans in cancer (ko05205), and alcoholism (ko05034) (Fig. 4D).

### Functional annotation of DE miRNAs

**Fig. 5:**
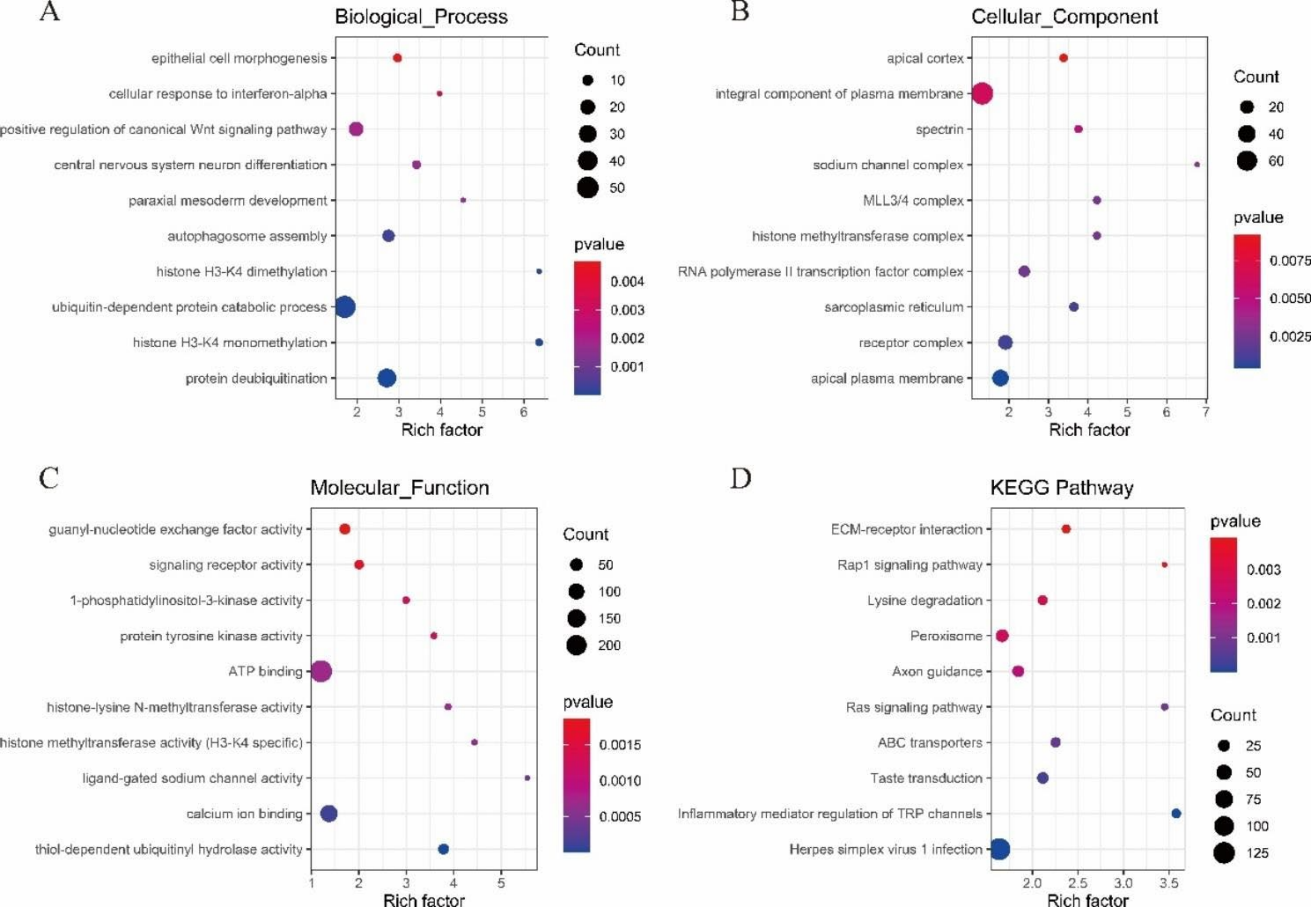
Functional annotation of DE miRNA target genes. (**A**) Biological process. (**B**) Cellular component. (**C**) Molecular function. (**D**). KEGG pathways

The 30 DE miRNAs predicted 3,970 target genes. Based on GO analysis, in the BP category, the target genes were mainly enriched in the protein deubiquitination (GO:0016579), ubiquitin-dependent protein catabolic process (GO:0006511), and positive regulation of the canonical Wnt signalling pathway (GO:0090263) (Fig. 5A). In the CC category, the target genes were mainly enriched in apical plasma membrane (GO:0016324), receptor complex (GO:0043235), and sarcoplasmic reticulum (GO:0016529) (Fig. 5B). In the MF category, target genes were mainly enriched in thiol-dependent ubiquitinyl hydrolase activity (GO:0036459), calcium ion binding (GO:0005509), and ligand-gated sodium channel activity (GO:0015280) (Fig. 5C). Similarly, KEGG annotation analysis showed that DE miRNA target genes were mainly enriched in inflammatory mediator regulation of TRP channels (ko04750), peroxisome (ko04146), and ECM-receptor interaction (ko04512) (Fig. 5D).

### Combined analysis of ncRNAs and coding genes

**Fig. 6:**
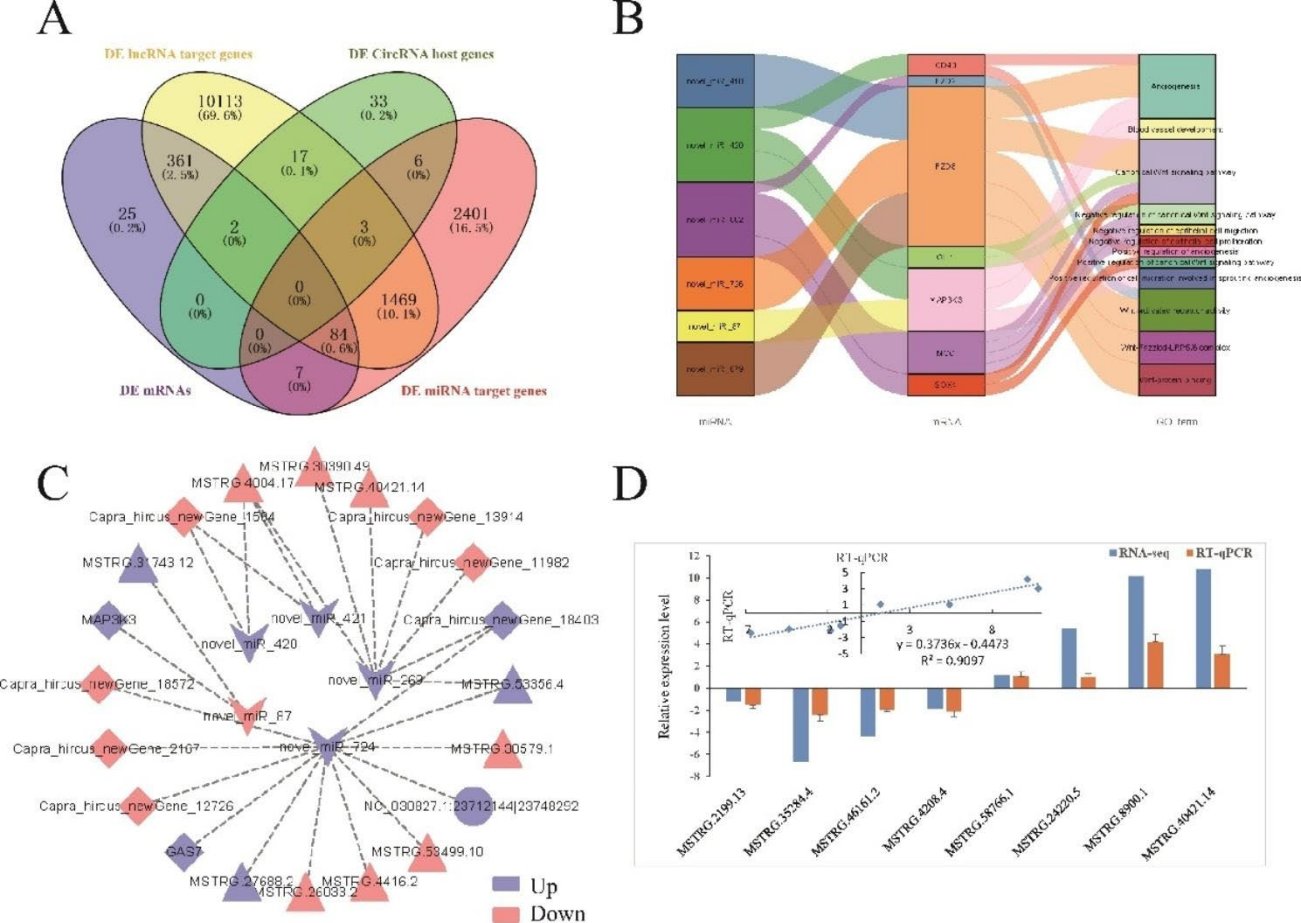
Combined analysis of coding RNA and noncoding RNA. (**A**) Venn diagrams of coding genes and ncRNAs. (**B**) Sankey diagram of DE miRNA target gene GO annotation. (**C**) The ceRNA network diagram. The diamond represents mRNA, triangle represents lncRNA, circle represents circRNA, and concave quadrilateral represents miRNA. (**D**) Validation of lncRNA by qRT-PCR.

To further understand the role of lncRNA, circRNA and miRNA in hair follicle development and cashmere growth, we conducted a joint analysis of ncRNA and coding RNA. In a previous study, 479 DE mRNAs were identified in the skin samples of the Fe and Ce groups of Jiangnan cashmere goats [1]. These DE mRNAs intersected with the target genes of DE lncRNAs and DE miRNAs, and the host genes of DE circRNAs in this study. A total of 396 DE lncRNAs had 447 predicted DE target genes, 2 DE circRNAs had 2 predicted DE host genes, and 17 DE miRNAs had 91 predicted DE target genes (Fig. 6A). The information of DE ncRNA and DE target genes is summarized in Table S3.

Combined with GO functional annotation analysis, we identified some DE target genes of DE lncRNAs annotated to epidermal and epithelial cell-related GO terms (Table S4). The number of *CXADR*, *MMP12* and *BCL9L* targeted lncRNAs ranked in the top three, with 19, 17 and 10, respectively. The number of GO terms with *KLF4*, *TGFB1I1* and *WNT5A* annotations ranked in the top three, which were 7, 3 and 3, respectively. In addition, the target genes of DE miRNA were annotated to the GO terms associated with skin, hair follicles, and angiogenesis and development. Six DE miRNAs (novel_miR_420, novel_miR_87, novel_miR_410, novel_miR_726, novel_miR_879, and novel_miR_662) had 7 predicted target genes (*CD40*, *MAP3K3*, *FZD8*, *SOX4*, *GLI1*, *MCC*, and *FZD2*) related to skin, hair follicle and angiogenesis and development (Fig. 6B).

Based on KEGG analysis, we summarized the information of DE lncRNA and DE miRNA target genes annotated to hair follicle development and hair growth-related pathways (Table S5). Among them, the number of target genes annotated to the MAPK signalling pathway (ko04016) was the largest.

### Competing endogenous RNA (ceRNA) network analysis

In this study, candidate ceRNA relationship pairs were obtained by targeting miRNA relationships. CeRNAs can regulate the expression of each other by competitively binding to miRNAs. Therefore, based on miRNA target prediction, we constructed a ceRNA network with 9 DE mRNAs, 5 DE miRNAs, 10 DE lncRNAs and 1 circRNA. It is worth noting that novel_miR_724 has the most most targeted (Fig. 6C).

### Validation of RNA-seq results by qRT-PCR

We randomly selected 8 DE lncRNAs (MSTRG.2199.13, MSTRG.35284.4, MSTRG.46161.2, MSTRG.4208.4, MSTRG.58766.1, MSTRG. MSTRG.24220.5, MSTRG.8900.1, and MSTRG.40421.14) for qRT-PCR analysis to verify the transcriptome sequencing results. According to Fig. 6D, the qRT-PCR detection results showed a consistent trend with the RNA-seq data, indicating that transcriptome sequencing data were reliable and could be used for subsequent tests.

## Discussion

The skin of mammals is the tissue that comes into direct contact with the environment. Skin has the functions of protection, sensation, secretion, excretion and respiration [23, 24]. Cashmere goat hair follicles are an important accessory organ of the skin and are divided into primary hair follicles and secondary hair follicles [25]. Changes in the microenvironment and gene expression profile of skin tissues affect the growth and development of hair follicles, thereby affecting mammalian hair growth [26]. At present, the study of the noncoding RNA involved in cashmere fineness traits is not comprehensive. Therefore, the study of ncRNA expression profiles can help to elucidate the molecular mechanism by which ncRNAs regulate the fineness of cashmere goats. In this study, transcriptome sequencing revealed that a large number of lncRNAs, miRNAs and circRNAs were enriched in the skin tissues of cashmere goats, and their expression patterns showed different levels of abundance (Fig. 1). We found that fewer circRNAs and miRNAs were identified in the skin tissue of cashmere goats, and the overall expression level of circRNAs in the skin tissue was low. Moreover, the number of DE miRNAs was lower in the Ce and Fe groups. Therefore, our analysis of circRNA and miRNA is limited.

The skin is divided into epidermis, dermis and subcutaneous tissue. The epidermis is composed of tightly arranged epithelial cells [27–29]. To further understand the function of ncRNAs, we functionally annotated the target genes of ncRNAs. We found a large number of DE lncRNA target genes are annotated to GO terms related to differentiation, development, and migration of epidermal or epithelial cells, including GO:0009913, GO:0008544, GO:0045606, GO:0035313, GO:0030855, GO:0050673, GO:0010669, GO:0060429, GO:0045198, GO:0060638, GO:0002009, GO:0016331, GO:0030857, GO:0010633, GO:0050680, GO:0060054, GO:0010718, and GO:0010632) (Table S3). Among them, MSTRG.42054.1 have three target genes, namely, CD40, BCL9L and MMP12. MSTRG.18602.3 have target genes such as MAP3K3, NCSTN, and TGFB1I1. It is worth noting that these target genes are differentially expressed in the skin tissues of coarse and fine groups of cashmere goats [1]. Among them, CD40 protein is widely expressed in hematopoietic cells as well as endothelial cells and is involved in immune defense function in vivo [30, 31]. The *TGFB1I1* gene regulates the activity of androgen receptors and is involved in the regulation of Wnt and TGFB signaling pathways [32]. Dermal papilla cells (DPC) located in the hairball part of the hair follicle express androgen receptors and key enzymes in androgen metabolism. Studies have shown that DPC in different body parts have different responses to androgens, thus leading to different hair growth conditions [33, 34].

The epidermis in the skin tissue has no blood supply, and the dermal papilla, located in the hairball part of the hair follicle, is connected to blood vessels. Therefore, the main source of nutrients for hair follicles is the dermal papilla. The size of the hairball part of the hair follicle is related to the hair thickness [35–37]. We also focused on DE lncRNA and DE miRNA target genes, including *CD40*, *MAP3K3*, *KLF4*, and *SOX18*, which were associated with vascular development (GO:0001568) and endothelial cell migration (GO:0043534, GO:0043537, GO:0043536, GO:0090050, and GO:0043535).

These results indicate that ncRNAs play an important role in skin and are involved in regulating hair growth.

In this study, the analysis of DE lncRNAs found that MSTRG.11813.4, MSTRG.28026.4, and MSTRG.26322.32 shared a common target gene *COL5A1*, which was annotated to skin development (GO: 0043588). The common target gene of MSTRG.49210.1, MSTRG.2199.13, MSTRG.34920.1, and MSTRG.25997.1 was *PTCH2*, which was annotated to hair cycle and development (GO: 0042633). We noted that the relative expression level of MSTRG.2199.13 was maesured by qRT-PCR, and the results were consistent with the trend of the RNA-seq results (Fig. 6D). The common target gene of MSTRG.6557.1, MSTRG.53893.2, MSTRG.27055.1 and MSTRG.51287.1 is SOX18, which is also involved in hair follicle development (GO:0001942). In our previous study, it was found that *COL5A1*, *PTCH2* and *SOX18* were differentially expressed in the Fe and Ce groups of Jiangnan cashmere goats [1]. These results indicated that the expression levels of *COL5A1*, *PTCH2* and *SOX18* in skin were regulated by lncRNAs. In addition, keratin intermediate filament and keratin association protein are the two main proteins in cashmere fibres. lncRNA analysis revealed that seven keratin family genes, including *KRT7*, *KRT36*, *LOC102188618*, *LOC102185150*, *LOC100861174*, *LOC108636556*, and *LOC102168573*, can target a total of 64 targeted DE lncRNAs. Activation of WNT signalling pathways in the skin is essential, and these pathways are related to normal hair follicle development, hair follicle distribution in the matrix, and hair phenotype [38]. Therefore, DE miRNA target gene annotation has attracted our attention in the GO term (GO:0090090, GO:0060070, GO:0090263, GO:0042813, GO:0017147, and GO:1,990,851) associated with the Wnt signalling pathway.

Different types of ncRNAs can synergistically play important regulatory functions at the transcriptional and posttranscriptional levels [21, 39]. The ceRNA mechanism based on microRNA response elements can explain the regulatory complexity of ncRNAs and coding RNAs to a certain extent. CeRNA-mediated gene regulation is an emerging area of research that will greatly increase our understanding of hair follicle development and hair growth [40–42]. Currently, through high-throughput sequencing and bioinformatics analysis, it has been reported that ceRNA networks with chi-miR-221-5p, chi-miR-214-3p, chi-miR-331-5p and chi-miR-17-5p as response elements play a regulatory role in the hair follicles of cashmere goats [5, 22]. In our study, some ceRNA networks were obtained. However, miRNA involvement in the network is novel (Fig. 6C). Therefore, their influence on the fineness of cashmere still needs further study.

## Conclusion

In this study, the changes in ncRNA expression profiles in skin samples of Jiangnan cashmere goats with different levels of fineness were identified. Combined with the results of previous studies, the regulatory roles of ncRNA and coding RNA in the fineness traits of cashmere were analysed. The results provide experimental data for interpreting hair follicle development and hair growth. In future studies, we will elucidate the molecular mechanisms by which ncRNAs regulate cashmere fineness at the molecular and cellular levels.

### Methods

#### Experimental animals

In this study, the experimental farm was the Baihutai cashmere goat breeding centre in Aksu Prefecture, Xinjiang. Eight 12-month-old cashmere goat ewes were selected as experimental animals. We divided the experimental animals into the Fe group (n = 4) and Ce group (n = 4), and their average fibre diameters were 13.64 ± 0.04 μm and 15.31 ± 0.04 μm, respectively. We collected skin tissue from the left shoulder blade of eight Jiangnan cashmere goats. The feeding conditions of the experimental animals were basically the same before and after the experiment. From May to October for grazing feeding, from November to April for grazing plus supplementary feeding.

### Sequencing of ncRNA

The RNA of skin tissue from 8 southern Xinjiang cashmere goats was extracted using TRIzol and and RNA extraction kit. Chain-specific and small RNA libraries were constructed for RNA of 8 samples. The constructed library was inspected by the Qsep-400 method. Eight chain-specific libraries and eight small RNA libraries were sequenced on an Illumina NovaSeq 6000.

The Base Quality Score was used to evaluate the raw sequencing data. The Capra_hircus reference genome (Capra hircus ARS 1.97) was used to compare lncRNA data with HISAT2 [43], and StringTie was used to compare read pairs. LncRNAs were identified by four methods: CPC analysis [44], CNCI analysis [45], CPAT analysis [46], and Pfam protein domain [47] analysis. Circular RNA prediction was performed using CIRI [48] and find_circ [49] software. Using Bowtie [49] software, clean reads of miRNA were mapped to reference genome. In addition, reads were compared in the miRBase (v22) database to identify known miRNAs. Novel miRNAs were predicted using miRDeep2 [50] software.

### Analysis of ncRNA expression levels

FPKM was used as a measure of lncRNA expression levels [51]. Statistics on circRNA expression were standardized using SRPBM methods. The miRNA expression was statistically normalized by the TPM algorithm [52]. Using fold change ≥ 2 and P value ≤ 0.05 as the standard, the edgeR package was used to identify out DE ncRNAs in the Fe and Ce groups, including DE lncRNAs, DE circRNAs and DE miRNAs.

### Prediction of ncRNA target genes

In lncRNA analysis, Perl scripts were used to identify cis target genes of DE lncRNA. The LncTar [53] target gene prediction tool was used to predict the trans target gene of DE lncRNAs based on the complementary sequence between DE lncRNAs and mRNAs. In the miRNA analysis, we used miRanda [48] and TargetScan [48] to predict the target genes of DE miRNAs. In addition, circRNAs contains multiple miRNA binding sites. Therefore, miRNA target gene prediction can be used to identify circRNA binding to miRNA. Finally, Cytoscape 3.6.1 software was used to visualize the ncRNA and target gene networks.

### Functional annotation of noncoding RNAs

To explain the function of ncRNAs, we annotated the function of lncRNA and miRNA target genes. Functional annotation of circRNA host genes was performed. BLAST software was used to compare genes with the NR [54], Swiss-Prot [55], GO [56], and KEGG [57] databases. The functional annotation of target genes was enriched and analysed. The bubble map was drawn by the ggplot2 package for the first 10 items or the combination with the lowest significant Q value.

### Verify of transcriptome sequencing results

To determine the reliability of the sequencing data, eight DE lncRNAs were randomly selected to verify their expression levels by qRT-PCR. Primers for DE lncRNA and *GAPGH* were designed with Primer 5.0 (Table S6). After transcriptome sequencing was complete, the RNA returned by the company was used as a template. The qRT-PCR procedure for lncRNA was performed in the Roche LightCycler 480 II fluoroquantitative PCR instrument with reference to previous experiments [1]. The qRT-PCR results were analysed by the 2^−ΔΔCt^ method.

### Electronic supplementary material

Below is the link to the electronic supplementary material.

**Supplementary Material 1: Figure S1**. Statistical analysis of noncoding RNA of 8 samples. **Table S1**. Quality control and reference genome alignment of lncRNA and circRNA sequencing. **Table S2**. Quality control and reference genome alignment of miRNA sequencing. **Table S3**. Summary of DE ncRNA and DE target mRNA. **Table S4**. Summary of GO annotation of non-coding RNAs target genes associated with epidermal epithelial cells. **Table S5**. KEGG annotation results of non-coding RNA target gene. **Table S6**. Primer information of lncRNA.

## Data Availability

The original sequencing data of chain-specific libraries and small RNA libraries are stored in the SRA database (Submission ID: PRJNA778726. Download address: https://www.ncbi.nlm.nih.gov/bioproject/PRJNA778726).
