# Supplementary material for: Regulation of cashmere fineness traits by noncoding RNA in Jiangnan cashmere goats
Source: BMC Genomics. 2023 Oct 11;24:604. doi: 10.1186/s12864-023-09531-x (PMC10566132; doi:10.1186/s12864-023-09531-x)
Supplement: Supplementary file 1 — Supplementary Material 1: Figure S1. Statistical analysis of noncoding RNA of 8 samples. Table S1. Quality control and reference genome alignment of lncRNA and circRNA sequencing. Table S2. Quality control and reference genome alignment of miRNA sequencing. Table S3. Summary of DE ncRNA and DE target mRNA. Table S4. Summary of GO annotation of non-coding RNAs target genes associated with epidermal epithelial cells. Table S5. KEGG annotation results of non-coding RNA target gene. Table S6. Primer information of lncRNA. [file 12864_2023_9531_MOESM1_ESM.pdf]

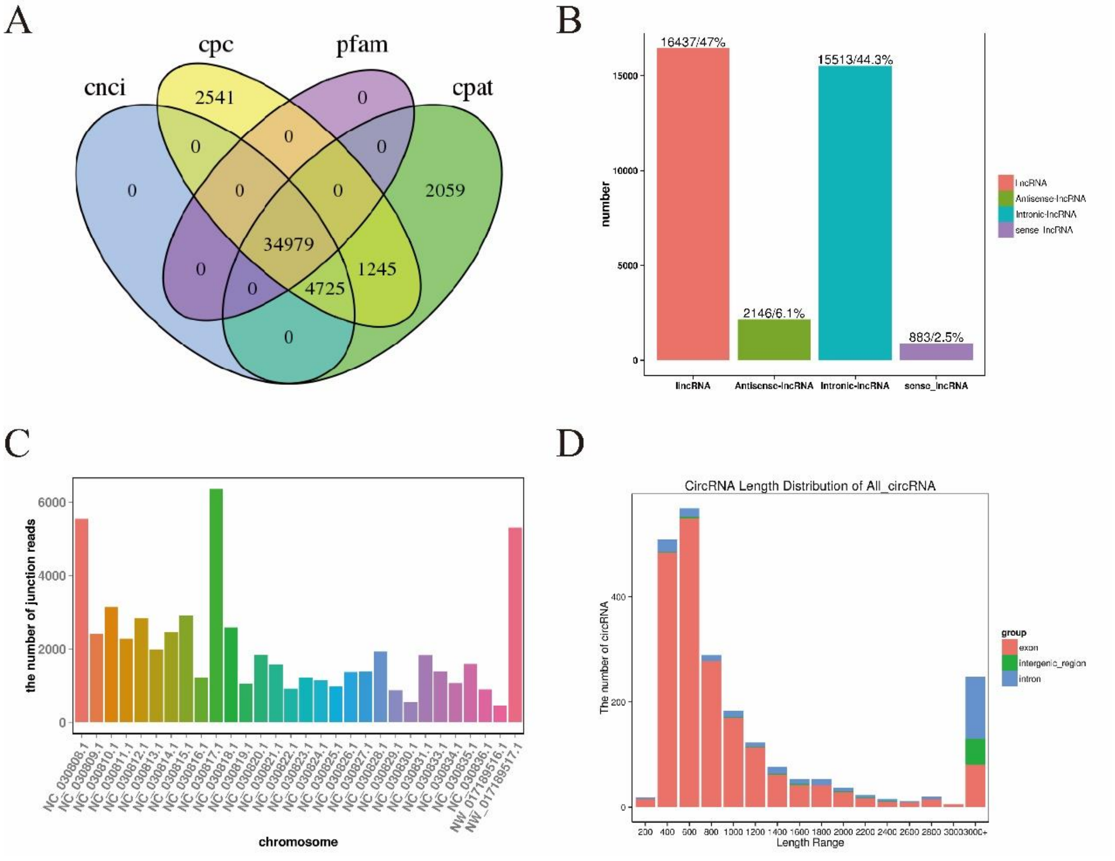

Figure S1. Statistical analysis of noncoding RNA of 8 samples. (A) Venn diagram for prediction of lncRNA. (B) Histogram of lncRNA classification. (C) Distribution histograms of CircRNA reads on different chromosomes. (D) Length distribution histogram of CircRNA.

Table S1. Quality control and reference genome alignment of lncRNA and circRNA sequencing

Samples	Clean Reads	Q30(%)	Mapped_Reads	Mapping ratio(%)
Ce-1	72,834,310	95.37	68,742,561	94.38%
Ce-2	74,438,410	95.15	70,220,158	94.33%
Ce-3	84,380,248	95.14	80,047,193	94.86%
Ce-4	74,761,636	95.65	70,419,223	94.19%
Fe-1	74,116,322	95.58	70,038,566	94.50%
Fe-2	78,545,638	95.03	74,231,526	94.51%
Fe-3	84,696,486	95.21	80,090,317	94.56%
Fe-4	77,443,290	95.14	73,391,963	94.77%

Table S2. Quality control and reference genome alignment of miRNA sequencing

Samples	Clean_Reads	Q30(%)	Mapped_Reads	Mapping ratio(%)
Ce-1	10,293,393	96.97	8,322,748	80.86%
Ce-2	11,864,022	96.93	9,334,693	78.68%
Ce-3	15,433,160	96.80	12,543,271	81.27%
Ce-4	14,287,618	96.22	11,765,882	82.35%
Fe-1	12,249,724	96.37	9,768,615	79.75%
Fe-2	12,908,762	96.90	10,812,528	83.76%
Fe-3	19,159,289	96.76	15,695,470	81.92%
Fe-4	18,127,238	96.53	14,575,385	80.41%

Table S3. Summary of DE ncRNA and DE target mRNA

DE ncRNA	Regulated	P value	log2Fold Change	Target mRNA (Regulated, P value, log2Fold Change)
MSTRG.10193.1	down	0.0017	-2.483	POC5 (up, 0.0476, 0.1785), TRAPPC13 (up, 0.0458, 0.1645), Capra_hircus_newGene_5067 (down, 0.0193, -0.1314), PTMS (down, 0.0402, -0.1617) PMM1 (down, 0.0377, -0.2179)
MSTRG.10266.2	up	0.0073	4.412	LOC102168852 (up, 0.0003, 0.3332), Capra_hircus_newGene_11982 (up, 0.0073, 0.3031), LOC102173111 (up, 0.0403, 0.2312), LOC102184901 (up, 0.0268, 0.2155)
MSTRG.10266.3	down	0.0275	-2.081	LOC102177708 (down, 0.0034, -0.3300), NET1 (down, 0.0475, -0.1192)
MSTRG.10287.9	up	0.0000	6.367	MYO3B (up, 0.0075, 0.3008), NELL2 (up, 0.0426, 0.2278), MRPS18B (up, 0.0189, 0.2197), MTHFD1 (up, 0.0114, 0.1881), C8H9orf72 (up, 0.0236, 0.1854), MRPS27 (up, 0.0254, 0.1649), MEIS3 (down, 0.0218, -0.2535), PRKD1 (down, 0.0013, -0.3107)
MSTRG.10297.1	up	0.0370	1.304	PNPT1 (up, 0.0173, 0.1880), TNFRSF18 (up, 0.0027, 0.3213), GRM2 (up, 0.0089, 0.2625), APLN (up, 0.0178, 0.2470), ITGA2 (up, 0.0262, 0.2254)

DE ncRNA	Regulated	P value	log2Fold Change	Target mRNA (Regulated, P value, log2Fold Change)
MSTRG.10693.1	up	0.0160	1.851	SLC5A6 (up, 0.001, 0.2799), TARM1 (up, 0.0096, 0.2777), Capra_hircus_newGene_17456 (up, 0.0337, 0.2401), LOC102172474 (up, 0.0379, 0.2348), PUS7 (up, 0.013, 0.2169), CDC40 (up, 0.0031, 0.2084), BCLAF1 (up, 0.0065, 0.1472), ZBTB6 (down, 0.0223, -0.1686), SLC15A4 (down, 0.0329, -0.1808), UTP14A (down, 0.0054, -0.1862), SOX4 (down, 0.0000, -1.1673)
MSTRG.10816.1	up	0.0287	2.090	TLR1 (up, 0.007, 0.2740), WRN (up, 0.0188, 0.1520), ATP13A4 (up, 0.0000, 0.5377), Capra_hircus_newGene_18572 (up, 0.0001, 0.4124), Capra_hircus_newGene_22856 (up, 0.0029, 0.3335), UROS (up, 0.0032, 0.3268), C1H3orf33 (up, 0.0077, 0.3012), CD200 (up, 0.0069, 0.2948), LOC102174470 (up, 0.0021, 0.2683), STAT4 (up, 0.0143, 0.2454), LOC102186466 (up, 0.0416, 0.2137), DCK (up, 0.034, 0.1895), PREB (up, 0.0229, 0.1666), CASP3 (up, 0.0416, 0.1591), RASA1 (up, 0.0378, 0.1485), BCL9L (down, 0.0444, -0.1451), AKAP17A (down, 0.0305, -0.1924), CARD10 (down, 0.0396, -0.1942), CD247 (down, 0.0151, -0.2586)
MSTRG.11.1	down	0.0465	-1.220	TLR1 (up, 0.007, 0.2740), WRN (up, 0.0188, 0.1520), ATP13A4 (up, 0.0000, 0.5377), C1H3orf33 (up, 0.0077, 0.3012), C14H8orf82 (up, 0.0302, 0.2351), MRPS18B (up, 0.0189, 0.2197), MTHFD1 (up, 0.0114, 0.1881), SNURF (up, 0.0418, 0.1668), CDCA7L (up, 0.015, 0.1647), TRAPPC13 (up, 0.0458, 0.1645), TMEM87A (up, 0.0164, 0.1548), ASB3 (up, 0.0331, 0.1443), Capra_hircus_newGene_5067 (down, 0.0193, -0.1314), PITX2 (down, 0.0453, -0.1976), AWAT1 (down, 0.0158, -0.2730)
MSTRG.1127.2	up	0.0300	1.791	FAM206A (up, 0.0444, 0.1890), Capra_hircus_newGene_5769 (up, 0.0088, 0.2831), TARM1 (up, 0.0096, 0.2777), Capra_hircus_newGene_12726 (up, 0.0159, 0.2591), DTD2 (up, 0.0089, 0.2513), TAF1D (up, 0.0177, 0.2439), GLMN (up, 0.0137, 0.2399), GRK2 (down, 0.0118, -0.1519), AIP (down, 0.0216, -0.1957), SLC25A19 (down, 0.049, -0.1966), Capra_hircus_newGene_11118 (down, 0.0467, -0.2228),
MSTRG.11466.14	down	0.0047	-3.038	AKR1B1 (down, 0.0117, -0.2830), LOC108634363 (down, 0.0361, -0.1015), CD248 (down, 0.0042, -0.3239)
MSTRG.11603.1	up	0.0412	2.470	BBOX1 (up, 0.0069, 0.2964)
MSTRG.11745.1	up	0.0094	1.850	FAM206A (up, 0.0444, 0.1890), Capra_hircus_newGene_9422 (up, 0.0058, 0.3034), GRM2 (up, 0.0089, 0.2625), APLN (up, 0.0178, 0.2470), ASMTL (down, 0.0037, -0.2855)
MSTRG.1176.8	up	0.0028	2.697	ZNRF2 (up, 0.0382, 0.1620), ZNF260 (up, 0.0000, 0.4445), Capra_hircus_newGene_9422 (up, 0.0058, 0.3034), TARM1 (up, 0.0096, 0.2777), Capra_hircus_newGene_1952 (up, 0.0029, 0.2627), FGD6 (up, 0.0034, 0.2506), Capra_hircus_newGene_17456 (up, 0.0337, 0.2401), TARS2 (up, 0.0062, 0.2366), FASTKD2 (up, 0.0031, 0.2242), CDC40 (up, 0.0031, 0.2084), NUP54 (up, 0.0119, 0.1988), IDH3A (up, 0.0434, 0.1643),

DE ncRNA	Regulated	P value	log2Fold Change	Target mRNA (Regulated, P value, log2Fold Change)
				ASMTL (down, 0.0037, -0.2855), ENPEP (down, 0.0054, -0.2921), SOX4 (down, 0.0000, -1.1673)
MSTRG.11778.1	down	0.0237	-1.853	TLR1 (up, 0.007, 0.2740), POU2F1 (up, 0.0325, 0.1270), ZNF260 (up, 0.0000, 0.4445), RAB37 (up, 0.013, 0.2690), LOC102183952 (up, 0.0381, 0.2228), PLEKHH2 (up, 0.0076, 0.2063), DHX36 (up, 0.0096, 0.1475), BCLAF1 (up, 0.0065, 0.1472), FYTTD1 (up, 0.0129, 0.1452), MATR3 (up, 0.0168, 0.1024)
MSTRG.11813.4	up	0.0006	5.185	PNPT1 (up, 0.0173, 0.1880), MARCH7 (up, 0.0025, 0.1770), TMEM221 (up, 0.0000, 0.3318), KRT7 (up, 0.0017, 0.3227), Capra_hircus_newGene_4795 (up, 0.0034, 0.2777), GLI1 (up, 0.0045, 0.2678), Capra_hircus_newGene_5523 (up, 0.0105, 0.2529), PRSS12 (up, 0.0286, 0.2448), SP2 (down, 0.0328, -0.1892), COL5A1 (down, 0.0315, -0.2375)
MSTRG.11895.1	up	0.0423	1.909	CXCR2 (up, 0.0253, 0.2180), AMD1 (up, 0.0459, 0.1660), LOC102172005 (up, 0.0281, 0.0851)
MSTRG.12102.1	up	0.0021	3.288	WRN (up, 0.0188, 0.1520), ATP13A4 (up, 0.0000, 0.5377), UROS (up, 0.0032, 0.3268), CD200 (up, 0.0069, 0.2948), KY (up, 0.0109, 0.2881), SCN8A (up, 0.0164, 0.2530), LOC102186466 (up, 0.0416, 0.2137), TMEM123 (up, 0.0093, 0.1929), DCK (up, 0.034, 0.1895), PREB (up, 0.0229, 0.1666), CASP3 (up, 0.0416, 0.1591), SOX4 (down, 0.0000, -1.1673)
MSTRG.12462.1	down	0.0001	-4.184	LOC106503979 (down, 0.0184, -0.2280), CNR1 (down, 0.0117, -0.2250), DPP6 (up, 0.033, 0.2408), TRPM6 (down, 0.044, -0.2277)
MSTRG.1280.11	down	0.0109	-1.637	UGGT2 (up, 0.027, 0.2456), RPE (up, 0.0204, 0.1995), FZD8 (up, 0.0352, 0.1967), SHTN1 (up, 0.0417, 0.1655), IDS (down, 0.0246, -0.1402), MEF2D (down, 0.0383, -0.1429), PPP1R7 (down, 0.0455, -0.1478), ATP8B2 (down, 0.0088, -0.1796), DYRK1B (down, 0.0187, -0.1879), PITX2 (down, 0.0453, -0.1976), DDAH2 (down, 0.0099, -0.2543), AADACL3 (down, 0.0000, -0.4595)
MSTRG.12948.1	up	0.0448	1.726	Capra_hircus_newGene_11982 (up, 0.0073, 0.3031), LOC102184252 (up, 0.0069, 0.2972), LOC102173760 (up, 0.0397, 0.1705)
MSTRG.1312.2	up	0.0360	1.363	LOC102176218 (down, 0.0131, -0.2770), WRN (up, 0.0188, 0.1520), Capra_hircus_newGene_18572 (up, 0.0001, 0.4124), LOC108633201 (up, 0.0009, 0.3354), TNFSF4 (up, 0.0257, 0.2385), TSR3 (up, 0.0188, 0.2326), FASTKD2 (up, 0.0031, 0.2242), CDC40 (up, 0.0031, 0.2084), C1D (up, 0.0062, 0.1999), METTL17 (up, 0.0256, 0.1940), CCDC50 (up, 0.0212, 0.1801), CXADR (up, 0.009, 0.1649), MCC (up, 0.0197, 0.1551), SRSF3 (up, 0.0396, 0.1062), ENGASE (down, 0.0116, -0.2122), THSD7A (down, 0.0294, -0.2382), LOC102174081 (down, 0.0163, -0.2513), SNX24 (down, 0.0081, -0.2668)

DE ncRNA	Regulated	P value	log2Fold Change	Target mRNA (Regulated, P value, log2Fold Change)
MSTRG.13301.2	down	0.0006	-3.759	LOC106503979 (down, 0.0184, -0.2280), FGD6 (up, 0.0034, 0.2506), SRXN1 (up, 0.0278, 0.2185), IDH3A (up, 0.0434, 0.1643), FHL5 (down, 0.032, -0.2352), YPEL1 (down, 0.0203, -0.2455)
MSTRG.13409.8	up	0.0287	2.853	FAM111B (up, 0.0007, 0.3640), CD40 (up, 0.0437, 0.2030), LOC108638522 (up, 0.0374, 0.1840), UROS (up, 0.0032, 0.3268), MMP12 (up, 0.0016, 0.2779), PTGS2 (up, 0.0081, 0.2594), SLC16A1 (up, 0.0401, 0.2321), KCNJ15 (up, 0.0286, 0.2298), PREB (up, 0.0229, 0.1666)
MSTRG.13429.1	down	0.0000	-4.844	ATP13A4 (up, 0.0000, 0.5377), Capra_hircus_newGene_18572 (up, 0.0001, 0.4124), TARM1 (up, 0.0096, 0.2777), Capra_hircus_newGene_1952 (up, 0.0029, 0.2627), FGD6 (up, 0.0034, 0.2506), Capra_hircus_newGene_17456 (up, 0.0337, 0.2401), TSR3 (up, 0.0188, 0.2326), FASTKD2 (up, 0.0031, 0.2242), CDC40 (up, 0.0031, 0.2084), CXADR (up, 0.009, 0.1649), PPP1R7 (down, 0.0455, -0.1478), LOC106503362 (down, 0.0208, -0.2331), SOX4 (down, 0.0000, -1.1673)
MSTRG.13439.1	up	0.0056	2.723	FAM111B (up, 0.0007, 0.3640), ZNF605 (up, 0.0085, 0.2370), Capra_hircus_newGene_22856 (up, 0.0029, 0.3335), UROS (up, 0.0032, 0.3268), C1H3orf33 (up, 0.0077, 0.3012), CD200 (up, 0.0069, 0.2948), LOC102174470 (up, 0.0021, 0.2683), CD48 (up, 0.0242, 0.2537), STAT4 (up, 0.0143, 0.2454), DCK (up, 0.034, 0.1895), PREB (up, 0.0229, 0.1666), CASP3 (up, 0.0416, 0.1591), RASA1 (up, 0.0378, 0.1485), BCL9L (down, 0.0444, -0.1451), CARD10 (down, 0.0396, -0.1942), CD247 (down, 0.0151, -0.2586)
MSTRG.13640.5	up	0.0002	3.945	MMP13 (up, 0.048, 0.1680), Capra_hircus_newGene_4795 (up, 0.0034, 0.2777), LOC108637962 (up, 0.0491, 0.1324), ITGA2 (up, 0.0262, 0.2254)
MSTRG.13931.1	up	0.0435	2.235	Capra_hircus_newGene_12726 (up, 0.0159, 0.2591), LOC102179921 (down, 0.0066, -0.2763)
MSTRG.14092.1	down	0.0266	-1.692	TPPP3 (down, 0.0084, -0.2747), RFX2 (down, 0.0003, -0.4097)
MSTRG.14521.1	up	0.0439	1.765	FAM206A (up, 0.0444, 0.1890), ZNF260 (up, 0.0000, 0.4445), RBM3 (up, 0.0002, 0.3306), Capra_hircus_newGene_9422 (up, 0.0058, 0.3034), MAT1A (up, 0.0042, 0.2817), LOC106503901 (up, 0.0144, 0.2708), RAB37 (up, 0.013, 0.2690), Capra_hircus_newGene_12726 (up, 0.0159, 0.2591), TAF1D (up, 0.0177, 0.2439), TSTD3 (up, 0.0406, 0.2313), RASSF8 (up, 0.0095, 0.2249), FSD1L (up, 0.0364, 0.2032), NUP54 (up, 0.0119, 0.1988), TRMT13 (up, 0.0348, 0.1971), CEP89 (down, 0.0356, -0.2204), PPP2R2B (down, 0.0363, -0.2234), ASMTL (down, 0.0037, -0.2855)
MSTRG.14664.1	up	0.0101	1.964	TLR1 (up, 0.007, 0.2740), ATP13A4 (up, 0.0000, 0.5377), UROS (up, 0.0032, 0.3268), C1H3orf33 (up, 0.0077, 0.3012), CD200 (up, 0.0069, 0.2948), CD48 (up, 0.0242, 0.2537), LOC102169889 (up, 0.0335, 0.2391), LOC102186466 (up, 0.0416, 0.2137), PLEKHH2 (up, 0.0076, 0.2063),

DE ncRNA	Regulated	P value	log2Fold Change	Target mRNA (Regulated, P value, log2Fold Change)
				TMEM123 (up, 0.0093, 0.1929), DCK (up, 0.034, 0.1895), PREB (up, 0.0229, 0.1666), CASP3 (up, 0.0416, 0.1591), BCL9L (down, 0.0444, -0.1451), AKAP17A (down, 0.0305, -0.1924)
MSTRG.1498.1	up	0.0448	2.156	Capra_hircus_newGene_2167 (up, 0.0015, 0.3595), Capra_hircus_newGene_22856 (up, 0.0029, 0.3335), KCND2 (up, 0.0141, 0.2751), RASA1 (up, 0.0378, 0.1485), DCTN2 (down, 0.0161, -0.1403), CARD10 (down, 0.0396, -0.1942), FZD2 (down, 0.0263, -0.2244)
MSTRG.15346.1	up	0.0321	1.729	LOC108635404 (up, 0.0002, 0.3433), LOC102172005 (up, 0.0281, 0.0851)
MSTRG.15362.3	up	0.0205	2.417	HS3ST1 (up, 0.0332, 0.1818), LOC102173760 (up, 0.0397, 0.1705), MATR3 (up, 0.0168, 0.1024), DNASE1L2 (down, 0.0235, -0.2394)
MSTRG.15398.40	up	0.0185	2.919	FAM206A (up, 0.0444, 0.1890), MAT1A (up, 0.0042, 0.2817), Capra_hircus_newGene_12726 (up, 0.0159, 0.2591), LOC106503969 (up, 0.0224, 0.2105), ASMTL (down, 0.0037, -0.2855)
MSTRG.15491.5	up	0.0454	2.647	LOC108634682 (up, 0.0499, 0.1580), LOC106503943 (up, 0.0485, 0.1846)
MSTRG.15771.7	up	0.0205	2.118	ZNF605 (up, 0.0085, 0.2370), RNF217 (down, 0.0289, -0.1620), LOC102168852 (up, 0.0003, 0.3332), ARHGEF1 (down, 0.041, -0.1163), CD247 (down, 0.0151, -0.2586)
MSTRG.16128.1	down	0.0321	-1.211	CABYR (up, 0.0000, 0.5116), Capra_hircus_newGene_1952 (up, 0.0029, 0.2627), RBBP8 (up, 0.0116, 0.2518), Capra_hircus_newGene_17456 (up, 0.0337, 0.2401), C25H16orf59 (up, 0.0398, 0.2287), WDTC1 (down, 0.0393, -0.1113), TSC1 (down, 0.0098, -0.1441), PPP1R7 (down, 0.0455, -0.1478), CTU1 (down, 0.0105, -0.1511), RUBCN (down, 0.0015, -0.1962), APBA1 (down, 0.0249, -0.2197), MARCH2 (down, 0.0155, -0.2434), ZBTB47 (down, 0.0011, -0.2435), YPEL1 (down, 0.0203, -0.2455), C28H10orf10 (down, 0.0283, -0.2478), PRKD1 (down, 0.0013, -0.3107), SEPTIN6 (down, 0.0016, -0.3371)
MSTRG.16139.1	up	0.0378	1.327	Capra_hircus_newGene_22856 (up, 0.0029, 0.3335), UROS (up, 0.0032, 0.3268), C1H3orf33 (up, 0.0077, 0.3012), CD200 (up, 0.0069, 0.2948), DCK (up, 0.034, 0.1895), RASA1 (up, 0.0378, 0.1485), FZD2 (down, 0.0263, -0.2244)
MSTRG.16280.1	up	0.0347	1.272	LOC108638522 (up, 0.0374, 0.1840), ZNRF2 (up, 0.0382, 0.1620), WRN (up, 0.0188, 0.1520), ZNF260 (up, 0.0000, 0.4445), Capra_hircus_newGene_21230 (up, 0.0007, 0.3532), SCN8A (up, 0.0164, 0.2530), TNFSF4 (up, 0.0257, 0.2385), FASTKD2 (up, 0.0031, 0.2242), CDC40 (up, 0.0031, 0.2084), PLEKHH2 (up, 0.0076, 0.2063), ASMTL (down, 0.0037, -0.2855), SOX4 (down, 0.0000, -1.1673)
MSTRG.16539.2	up	0.0178	1.760	MMP13 (up, 0.048, 0.1680), LOC108635404 (up, 0.0002, 0.3433), LOC108635390 (up, 0.0014, 0.3178), LOC102176870 (up, 0.0088, 0.2564), LOC102172005 (up, 0.0281, 0.0851), CERCAM (down, 0.0179, -0.2276)

DE ncRNA	Regulated	P value	log2Fold Change	Target mRNA (Regulated, P value, log2Fold Change)
MSTRG.16644.1	down	0.0075	-2.492	CCDC22 (down, 0.0389, -0.1870), Capra_hircus_newGene_17456 (up, 0.0337, 0.2401), BCAP31 (down, 0.0218, -0.1273), PNPLA6 (down, 0.0213, -0.1405), SLC9A3R2 (down, 0.0483, -0.1615), SLC15A4 (down, 0.0329, -0.1808), MRPL47 (down, 0.0424, -0.1918), ARFIP1 (down, 0.0054, -0.2133), CLCC1 (down, 0.0063, -0.2214), HIC1 (down, 0.0303, -0.2448), LOC102189713 (down, 0.0178, -0.2676), MXD4 (down, 0.0019, -0.2706), PRKCDBP (down, 0.0011, -0.3262), MRGPRF (down, 0.0001, -0.4224)
MSTRG.16858.1	down	0.0035	-2.645	EHD2 (down, 0.0092, -0.2020), CCDC22 (down, 0.0389, -0.1870), Capra_hircus_newGene_1952 (up, 0.0029, 0.2627), FGD6 (up, 0.0034, 0.2506), Capra_hircus_newGene_17456 (up, 0.0337, 0.2401), PNPLA6 (down, 0.0213, -0.1405), TSC1 (down, 0.0098, -0.1441), MRPL47 (down, 0.0424, -0.1918), CLCC1 (down, 0.0063, -0.2214), LOC102189713 (down, 0.0178, -0.2676), CDH13 (down, 0.0049, -0.2895), MRGPRF (down, 0.0001, -0.4224)
MSTRG.17073.1	down	0.0009	-3.256	AKR1B1 (down, 0.0117, -0.2830), PPAT (up, 0.0255, 0.2475), CDCA7L (up, 0.015, 0.1647), LOC108634363 (down, 0.0361, -0.1015), PMM1 (down, 0.0377, -0.2179), OTOGL (down, 0.0094, -0.2432)
MSTRG.17114.1	up	0.0297	2.422	RPS5 (down, 0.0327, -0.1550), LOC108635404 (up, 0.0002, 0.3433), LOC108635390 (up, 0.0014, 0.3178), Capra_hircus_newGene_2169 (up, 0.0211, 0.2286), DDX52 (up, 0.0099, 0.2227), RPE (up, 0.0204, 0.1995), IDS (down, 0.0246, -0.1402), DYRK1B (down, 0.0187, -0.1879), SLC5A8 (down, 0.0181, -0.2358)
MSTRG.17227.2	up	0.0328	2.203	LOC102168852 (up, 0.0003, 0.3332), LOC102184252 (up, 0.0069, 0.2972), CD200 (up, 0.0069, 0.2948), LOC102174470 (up, 0.0021, 0.2683), STAT4 (up, 0.0143, 0.2454), LOC102169889 (up, 0.0335, 0.2391), Capra_hircus_newGene_15766 (up, 0.0481, 0.2098), PREB (up, 0.0229, 0.1666), CASP3 (up, 0.0416, 0.1591), BCL9L (down, 0.0444, -0.1451), AKAP17A (down, 0.0305, -0.1924)
MSTRG.17363.2	up	0.0332	2.281	FAM111B (up, 0.0007, 0.3640), C1H3orf33 (up, 0.0077, 0.3012), NEK6 (up, 0.0273, 0.2275), PEX12 (up, 0.032, 0.2129), CCDC50 (up, 0.0212, 0.1801), Capra_hircus_newGene_21088 (down, 0.0183, -0.1713), SIDT2 (down, 0.0011, -0.2037), LOC106503362 (down, 0.0208, -0.2331)
MSTRG.17924.3	up	0.0000	5.935	Capra_hircus_newGene_14773 (up, 0.0472, 0.0905)
MSTRG.18252.1	down	0.0232	-1.924	LOC100861174 (down, 0.0467, -0.2150), CMYA5 (up, 0.0425, 0.1700), WRN (up, 0.0188, 0.1520), PPAT (up, 0.0255, 0.2475), DIO2 (up, 0.0195, 0.2441), DDX52 (up, 0.0099, 0.2227), METTL17 (up, 0.0256, 0.1940), SLC31A1 (up, 0.0102, 0.1874), KDM4C (up, 0.0411, 0.1565), MCC (up, 0.0197, 0.1551), SRSF3 (up, 0.0396, 0.1062), PMM1 (down, 0.0377, -0.2179), LOC102174081 (down, 0.0163, -0.2513)

DE ncRNA	Regulated	P value	log2Fold Change	Target mRNA (Regulated, P value, log2Fold Change)
MSTRG.18321.5	down	0.0448	-2.410	LOC102185150 (down, 0.0323, -0.1983), FHL5 (down, 0.032, -0.2352)
MSTRG.18451.4	down	0.0000	-9.577	COG1 (down, 0.0003, -0.3120), Capra_hircus_newGene_1952 (up, 0.0029, 0.2627), FGD6 (up, 0.0034, 0.2506), TSR3 (up, 0.0188, 0.2326), FASTKD2 (up, 0.0031, 0.2242), CDC40 (up, 0.0031, 0.2084), CXADR (up, 0.009, 0.1649), PPP1R7 (down, 0.0455, -0.1478), ENGASE (down, 0.0116, -0.2122), LOC106503362 (down, 0.0208, -0.2331), YPEL1 (down, 0.0203, -0.2455), SOX4 (down, 0.0000, -1.1673)
MSTRG.1860.4	down	0.0351	-1.932	KLHL29 (up, 0.0013, 0.2747), CA4 (up, 0.0203, 0.2598), LOC102182782 (up, 0.0312, 0.2428), POC5 (up, 0.0476, 0.1785), Capra_hircus_newGene_21088 (down, 0.0183, -0.1713)
MSTRG.18602.3	down	0.0387	-1.805	ADAM22 (down, 0.0158, -0.2070), FUCA1 (down, 0.0231, -0.2070), WDTC1 (down, 0.0393, -0.1113), NCSTN (down, 0.0307, -0.1114), OS9 (down, 0.037, -0.1297), AMBRA1 (down, 0.0198, -0.1326), ALKBH5 (down, 0.0214, -0.1488), PPM1A (down, 0.0219, -0.1521), MAP3K3 (down, 0.0342, -0.1613), TOX2 (down, 0.0444, -0.1819), TEF (down, 0.0243, -0.1869), PTRF (down, 0.0451, -0.1989), C28H10orf10 (down, 0.0283, -0.2478), TGFB1I1 (down, 0.0171, -0.2526), TSPAN33 (down, 0.0138, -0.2583), STC2 (down, 0.0053, -0.3048), SEPTIN6 (down, 0.0016, -0.3371)
MSTRG.18755.1	up	0.0418	1.944	LOC102178853 (up, 0.0085, 0.2790), CXCR2 (up, 0.0253, 0.2180), ZNRF2 (up, 0.0382, 0.1620), Capra_hircus_newGene_21230 (up, 0.0007, 0.3532), SCN8A (up, 0.0164, 0.2530), RRH (up, 0.0277, 0.2351), DDX52 (up, 0.0099, 0.2227), Capra_hircus_newGene_10897 (up, 0.044, 0.2049), NT5C2 (up, 0.0483, 0.1682), ENPP4 (down, 0.0217, -0.2271), CERCAM (down, 0.0179, -0.2276), SNX24 (down, 0.0081, -0.2668), TIE1 (down, 0.0065, -0.3025)
MSTRG.18859.1	up	0.0395	1.278	PLEKHH2 (up, 0.0076, 0.2063), LOC106503943 (up, 0.0485, 0.1846)
MSTRG.1904.1	down	0.0200	-2.191	RNASEH2C (down, 0.0152, -0.2724)
MSTRG.19133.1	down	0.0407	-2.448	ATP8B2 (down, 0.0088, -0.1796), DDAH2 (down, 0.0099, -0.2543)
MSTRG.19158.1	up	0.0053	1.910	ZNRF2 (up, 0.0382, 0.1620), WRN (up, 0.0188, 0.1520), ATP13A4 (up, 0.0000, 0.5377), Capra_hircus_newGene_18572 (up, 0.0001, 0.4124), UROS (up, 0.0032, 0.3268), Capra_hircus_newGene_1952 (up, 0.0029, 0.2627), SCN8A (up, 0.0164, 0.2530), FASTKD2 (up, 0.0031, 0.2242), CDC40 (up, 0.0031, 0.2084), DCK (up, 0.034, 0.1895), PREB (up, 0.0229, 0.1666), CXADR (up, 0.009, 0.1649), PPP1R7 (down, 0.0455, -0.1478), STMN3 (down, 0.0379, -0.1576), GDA (down, 0.0318, -0.2011), SNX24 (down, 0.0081, -0.2668), SOX4 (down, 0.0000, -1.1673)

DE ncRNA	Regu lated	P value	log2Fold Change	Target mRNA (Regulated, P value, log2Fold Change)
MSTRG.19 779.1	down	0.0084	-1.670	ZNF165 (up, 0.0284, 0.2438), PITX2 (down, 0.0453, -0.1976), SNRPN (down, 0.016, -0.1981), TPPP3 (down, 0.0084, -0.2747), RFX2 (down, 0.0003, -0.4097)
MSTRG.19 794.8	up	0.0056	1.962	FAM206A (up, 0.0444, 0.1890), Capra_hircus_newGene_9422 (up, 0.0058, 0.3034), MAT1A (up, 0.0042, 0.2817), Capra_hircus_newGene_12726 (up, 0.0159, 0.2591), DTD2 (up, 0.0089, 0.2513), TAF1D (up, 0.0177, 0.2439), TSTD3 (up, 0.0406, 0.2313)
MSTRG.19 868.7	up	0.0142	1.848	RALGPS2 (up, 0.0216, 0.1380), LOC102174170 (up, 0.0065, 0.3036), Capra_hircus_newGene_9735 (up, 0.0153, 0.2735), MTHFD1 (up, 0.0114, 0.1881), RET (up, 0.0156, 0.1862), MRPS27 (up, 0.0254, 0.1649), ALG2 (down, 0.034, -0.1464), PRKD1 (down, 0.0013, -0.3107), GAS1 (down, 0.0002, -0.3759)
MSTRG.20 09.1	down	0.0278	-1.379	LOC102177708 (down, 0.0034, -0.3300), UGGT2 (up, 0.027, 0.2456), ATP8B2 (down, 0.0088, -0.1796)
MSTRG.20 743.2	down	0.0400	-1.722	SSR2 (down, 0.035, -0.1160), Capra_hircus_newGene_15790 (up, 0.0496, 0.2215), KLHL21 (down, 0.0117, -0.1713), RIMS3 (down, 0.0428, -0.2172), Capra_hircus_newGene_1595 (down, 0.0044, -0.2963)
MSTRG.20 859.1	up	0.0075	1.930	SLC5A6 (up, 0.001, 0.2799), KCND2 (up, 0.0141, 0.2751), NELL2 (up, 0.0426, 0.2278), MRPS18B (up, 0.0189, 0.2197), C8H9orf72 (up, 0.0236, 0.1854), ARHGEF1 (down, 0.041, -0.1163), GAS7 (down, 0.009, -0.1735), UTP14A (down, 0.0054, -0.1862), MTRR (down, 0.049, -0.1905), MRGPRF (down, 0.0001, -0.4224)
MSTRG.20 864.1	up	0.0200	1.803	MMP13 (up, 0.048, 0.1680), LOC108635404 (up, 0.0002, 0.3433), LOC108635390 (up, 0.0014, 0.3178), EPPK1 (up, 0.012, 0.2178), STMN3 (down, 0.0379, -0.1576), CERCAM (down, 0.0179, -0.2276), LSAMP (down, 0.0286, -0.2352), SIGLEC1 (down, 0.0113, -0.2555), LOC108638594 (down, 0.0155, -0.2654)
MSTRG.20 971.1	down	0.0254	-1.723	LOC102177708 (down, 0.0034, -0.3300), RNF217 (down, 0.0289, -0.1620), FZD8 (up, 0.0352, 0.1967), C8H9orf72 (up, 0.0236, 0.1854), ATP8B2 (down, 0.0088, -0.1796), PITX2 (down, 0.0453, -0.1976), PRKD1 (down, 0.0013, -0.3107)
MSTRG.21 044.1	down	0.0235	-2.423	LOC102185150 (down, 0.0323, -0.1983)
MSTRG.21 163.1	up	0.0156	1.591	ZNRF2 (up, 0.0382, 0.1620), Capra_hircus_newGene_9422 (up, 0.0058, 0.3034), MAT1A (up, 0.0042, 0.2817), TARM1 (up, 0.0096, 0.2777), FGD6 (up, 0.0034, 0.2506), TAF1D (up, 0.0177, 0.2439), Capra_hircus_newGene_17456 (up, 0.0337, 0.2401), GLMN (up, 0.0137, 0.2399), Capra_hircus_newGene_12245 (up, 0.0185, 0.2382), LOC106503969 (up, 0.0224, 0.2105), CDC40 (up, 0.0031, 0.2084), FSD1L (up, 0.0364, 0.2032), NUP54 (up, 0.0119, 0.1988), TRMT13 (up, 0.0348,

DE ncRNA	Regulated	P value	log2Fold Change	Target mRNA (Regulated, P value, log2Fold Change)
				0.1971), ARFIP1 (down, 0.0054, -0.2133), CLCC1 (down, 0.0063, -0.2214), ASMTL (down, 0.0037, -0.2855), SOX4 (down, 0.0000, -1.1673)
MSTRG.21516.2	up	0.0414	1.143	MYO3B (up, 0.0075, 0.3008), SLC5A6 (up, 0.001, 0.2799), Capra_hircus_newGene_9735 (up, 0.0153, 0.2735), MRPS18B (up, 0.0189, 0.2197), MTHFD1 (up, 0.0114, 0.1881), C8H9orf72 (up, 0.0236, 0.1854), TMEM87A (up, 0.0164, 0.1548), ZBTB6 (down, 0.0223, -0.1686), AWAT1 (down, 0.0158, -0.2730)
MSTRG.21578.2	up	0.0433	2.013	PNPT1 (up, 0.0173, 0.1880), MARCH7 (up, 0.0025, 0.1770), TMEM221 (up, 0.0000, 0.3318), LOC108635390 (up, 0.0014, 0.3178), Capra_hircus_newGene_4795 (up, 0.0034, 0.2777), RRH (up, 0.0277, 0.2351), ITGA2 (up, 0.0262, 0.2254), EPPK1 (up, 0.012, 0.2178), KCNA5 (down, 0.0262, -0.1884), CERCAM (down, 0.0179, -0.2276), THSD7A (down, 0.0294, -0.2382), TIE1 (down, 0.0065, -0.3025)
MSTRG.21662.3	down	0.0119	-2.376	ZNF423 (down, 0.026, -0.2370), ADAM22 (down, 0.0158, -0.2070), ADIPOR2 (down, 0.0441, -0.1419), SLC9A3R2 (down, 0.0483, -0.1615), KLHL21 (down, 0.0117, -0.1713), MRPL47 (down, 0.0424, -0.1918), PTRF (down, 0.0451, -0.1989), GFAP (down, 0.0251, -0.2006), BICDL2 (down, 0.0357, -0.2026), FBXL22 (down, 0.0168, -0.2050), ARFIP1 (down, 0.0054, -0.2133), RIMS3 (down, 0.0428, -0.2172), RAB3IL1 (down, 0.0492, -0.2194), LHX6 (down, 0.0316, -0.2381), HIC1 (down, 0.0303, -0.2448), LOC102189713 (down, 0.0178, -0.2676), MXD4 (down, 0.0019, -0.2706), ANGPTL1 (down, 0.0101, -0.2857), CDH13 (down, 0.0049, -0.2895), SERTM1 (down, 0.0056, -0.3107)
MSTRG.21713.12	down	0.0357	-1.628	ZNF605 (up, 0.0085, 0.2370), CANT1 (down, 0.0341, -0.1450), SSR2 (down, 0.035, -0.1160), Capra_hircus_newGene_5769 (up, 0.0088, 0.2831), NCSTN (down, 0.0307, -0.1114), BCAP31 (down, 0.0218, -0.1273), ADIPOR2 (down, 0.0441, -0.1419), GRK2 (down, 0.0118, -0.1519), PPM1A (down, 0.0219, -0.1521), KLHL21 (down, 0.0117, -0.1713), TOX2 (down, 0.0444, -0.1819), MRPL47 (down, 0.0424, -0.1918), GFAP (down, 0.0251, -0.2006), ZBTB47 (down, 0.0011, -0.2435), HIC1 (down, 0.0303, -0.2448), SEPN1 (down, 0.0023, -0.2478), MEX3B (down, 0.0251, -0.2489), TSPAN33 (down, 0.0138, -0.2583), MXD4 (down, 0.0019, -0.2706), STC2 (down, 0.0053, -0.3048), SERTM1 (down, 0.0056, -0.3107), SPON2 (down, 0.0011, -0.3677), GAS1 (down, 0.0002, -0.3759), MRGPRF (down, 0.0001, -0.4224), SOX4 (down, 0.0000, -1.1673)
MSTRG.21713.8	down	0.0487	-1.768	LOC102168573 (up, 0.0023, 0.2800), LOC102174170 (up, 0.0065, 0.3036), ZNF165 (up, 0.0284, 0.2438), TPPP (down, 0.0373, -0.2333)
MSTRG.2199.13	down	0.0195	-1.402	TLR1 (up, 0.007, 0.2740), C14H8orf82 (up, 0.0302, 0.2351), PTCH2 (up, 0.0088, 0.1971), MTHFD1 (up, 0.0114, 0.1881), SNURF (up, 0.0418, 0.1668), CDCA7L (up, 0.015, 0.1647), TRAPPC13 (up, 0.0458, 0.1645), TMEM87A (up, 0.0164, 0.1548), ASB3 (up, 0.0331, 0.1443),

DE ncRNA	Regulated	P value	log2Fold Change	Target mRNA (Regulated, P value, log2Fold Change)
				Capra_hircus_newGene_5067 (down, 0.0193, -0.1314), PTMS (down, 0.0402, -0.1617), PMM1 (down, 0.0377, -0.2179), AWAT1 (down, 0.0158, -0.2730), Capra_hircus_newGene_3416 (down, 0.0015, -0.3259), DZIP1L (down, 0.0009, -0.3689), RFX2 (down, 0.0003, -0.4097)
MSTRG.22069.1	up	0.0169	2.006	PNPT1 (up, 0.0173, 0.1880), LOC108635390 (up, 0.0014, 0.3178), GRM2 (up, 0.0089, 0.2625), RRH (up, 0.0277, 0.2351), DDX52 (up, 0.0099, 0.2227), C1D (up, 0.0062, 0.1999), RPE (up, 0.0204, 0.1995), KCNA5 (down, 0.0262, -0.1884), ENPP4 (down, 0.0217, -0.2271), CERCAM (down, 0.0179, -0.2276), THSD7A (down, 0.0294, -0.2382), OTOGL (down, 0.0094, -0.2432), TIE1 (down, 0.0065, -0.3025), DZIP1L (down, 0.0009, -0.3689)
MSTRG.22083.13	up	0.0094	2.248	LOC102184252 (up, 0.0069, 0.2972), MMP12 (up, 0.0016, 0.2779), LOC102174470 (up, 0.0021, 0.2683), LOC102169889 (up, 0.0335, 0.2391), PREB (up, 0.0229, 0.1666)
MSTRG.22301.1	up	0.0463	2.378	FAM111B (up, 0.0007, 0.3640), MMP12 (up, 0.0016, 0.2779), LOC102169889 (up, 0.0335, 0.2391), KCNJ15 (up, 0.0286, 0.2298), PREB (up, 0.0229, 0.1666)
MSTRG.22307.3	down	0.0014	-4.998	UGGT2 (up, 0.027, 0.2456), KCNA1 (up, 0.0459, 0.2129), TXNDC5 (up, 0.0176, 0.1726), ATP8B2 (down, 0.0088, -0.1796), CORIN (down, 0.01, -0.2553), MN1 (down, 0.0145, -0.2759)
MSTRG.22324.25	up	0.0086	2.806	FMO4 (up, 0.006, 0.3088), LOC102174170 (up, 0.0065, 0.3036), CROT (up, 0.0482, 0.2166), CIDEA (down, 0.0015, -0.3405)
MSTRG.22357.2	down	0.0333	-1.534	TRAPPC13 (up, 0.0458, 0.1645), PTMS (down, 0.0402, -0.1617)
MSTRG.22989.3	up	0.0392	2.265	SGPP1 (up, 0.0111, 0.2210), SLIT1 (up, 0.0482, 0.1882), Capra_hircus_newGene_14773 (up, 0.0472, 0.0905)
MSTRG.22991.7	up	0.0237	1.399	IRF2BPL (up, 0.0029, 0.3350), SGPP1 (up, 0.0111, 0.2210), CMYA5 (up, 0.0425, 0.1700), KCND2 (up, 0.0141, 0.2751), NEK6 (up, 0.0273, 0.2275), CCDC50 (up, 0.0212, 0.1801), SIDT2 (down, 0.0011, -0.2037), Capra_hircus_newGene_3630 (down, 0.0351, -0.2138),
MSTRG.23143.8	up	0.0374	2.576	LOC108638522 (up, 0.0374, 0.1840), Capra_hircus_newGene_5523 (up, 0.0105, 0.2529), PLEKHH2 (up, 0.0076, 0.2063)
MSTRG.23282.9	down	0.0493	-2.076	SIPA1L2 (down, 0.0443, -0.1743), CAV1 (down, 0.0218, -0.1919), FHL5 (down, 0.032, -0.2352), LOC102189713 (down, 0.0178, -0.2676), ANGPTL1 (down, 0.0101, -0.2857)
MSTRG.23434.10	down	0.0316	-1.516	COG1 (down, 0.0003, -0.3120), CNR1 (down, 0.0117, -0.2250), SLC35G1 (up, 0.0253, 0.2343), C25H16orf59 (up, 0.0398, 0.2287), TXNDC5 (up, 0.0176, 0.1726), YPEL1 (down, 0.0203, -0.2455), CLMN (down, 0.0039, -0.3259)

DE ncRNA	Regulated	P value	log2Fold Change	Target mRNA (Regulated, P value, log2Fold Change)
MSTRG.23593.17	up	0.0000	15.531	FAM111B (up, 0.0007, 0.3640), CD40 (up, 0.0437, 0.2030), MMP12 (up, 0.0016, 0.2779), PTGS2 (up, 0.0081, 0.2594), SPTA1 (up, 0.0153, 0.2057), CHST2 (up, 0.0049, 0.1866), HS3ST1 (up, 0.0332, 0.1818)
MSTRG.23680.2	up	0.0001	3.991	FAM111B (up, 0.0007, 0.3640), ZNF605 (up, 0.0085, 0.2370), Capra_hircus_newGene_18572 (up, 0.0001, 0.4124), Capra_hircus_newGene_22856 (up, 0.0029, 0.3335), UROS (up, 0.0032, 0.3268), LOC102184252 (up, 0.0069, 0.2972), CD200 (up, 0.0069, 0.2948), MMP12 (up, 0.0016, 0.2779), LOC102174470 (up, 0.0021, 0.2683), STAT4 (up, 0.0143, 0.2454), FASTKD2 (up, 0.0031, 0.2242), UAP1 (up, 0.0394, 0.2023), DCK (up, 0.034, 0.1895), PREB (up, 0.0229, 0.1666), MATR3 (up, 0.0168, 0.1024), BCL9L (down, 0.0444, -0.1451)
MSTRG.23897.21	down	0.0457	-2.377	FUCA1 (down, 0.0231, -0.2070), CANT1 (down, 0.0341, -0.1450), SSR2 (down, 0.035, -0.1160), CAPNS1 (down, 0.0452, -0.1094), BCAP31 (down, 0.0218, -0.1273), SEPN1 (down, 0.0023, -0.2478), GPR153 (down, 0.0185, -0.2531), TSPAN33 (down, 0.0138, -0.2583), STC2 (down, 0.0053, -0.3048)
MSTRG.24042.9	up	0.0343	1.762	ZNF605 (up, 0.0085, 0.2370), DCK (up, 0.034, 0.1895), DCTN2 (down, 0.0161, -0.1403), GAS7 (down, 0.009, -0.1735), SLC7A7 (down, 0.0239, -0.2352), THBS2 (down, 0.008, -0.2837)
MSTRG.24220.5	up	0.0000	11.351	LOC102168573 (up, 0.0023, 0.2800), Capra_hircus_newGene_11982 (up, 0.0073, 0.3031), Capra_hircus_newGene_9735 (up, 0.0153, 0.2735), LOC102172474 (up, 0.0379, 0.2348), MTHFD1 (up, 0.0114, 0.1881), BCLAF1 (up, 0.0065, 0.1472), ZBTB6 (down, 0.0223, -0.1686), LOC102179921 (down, 0.0066, -0.2763)
MSTRG.24398.1	down	0.0123	-1.422	TLR1 (up, 0.007, 0.2740), WRN (up, 0.0188, 0.1520), POU2F1 (up, 0.0325, 0.1270), ATP13A4 (up, 0.0000, 0.5377), Capra_hircus_newGene_18572 (up, 0.0001, 0.4124), FASTKD2 (up, 0.0031, 0.2242), LOC102183952 (up, 0.0381, 0.2228), CDC40 (up, 0.0031, 0.2084), DHX36 (up, 0.0096, 0.1475), BCLAF1 (up, 0.0065, 0.1472), FYTTD1 (up, 0.0129, 0.1452), MATR3 (up, 0.0168, 0.1024)
MSTRG.24585.1	up	0.0425	1.209	LOC108638522 (up, 0.0374, 0.1840), ZNRF2 (up, 0.0382, 0.1620), WRN (up, 0.0188, 0.1520), Capra_hircus_newGene_18572 (up, 0.0001, 0.4124), Capra_hircus_newGene_21230 (up, 0.0007, 0.3532), UROS (up, 0.0032, 0.3268), SCN8A (up, 0.0164, 0.2530), TSR3 (up, 0.0188, 0.2326), FASTKD2 (up, 0.0031, 0.2242), CDC40 (up, 0.0031, 0.2084), DDX47 (up, 0.0314, 0.1675), CXADR (up, 0.009, 0.1649), SNX24 (down, 0.0081, -0.2668), SOX4 (down, 0.0000, -1.1673)
MSTRG.24679.39	up	0.0066	4.765	MAT1A (up, 0.0042, 0.2817), LOC106503943 (up, 0.0485, 0.1846), Capra_hircus_newGene_6219 (up, 0.0388, 0.1808), MCC (up, 0.0197, 0.1551)

DE ncRNA	Regulated	P value	log2Fold Change	Target mRNA (Regulated, P value, log2Fold Change)
MSTRG.24731.3	down	0.0077	-4.210	CANT1 (down, 0.0341, -0.1450), FZD8 (up, 0.0352, 0.1967), MEF2D (down, 0.0383, -0.1429), ALKBH5 (down, 0.0214, -0.1488), Capra_hircus_newGene_8638 (down, 0.0074, -0.1663), GPR153 (down, 0.0185, -0.2531), DDAH2 (down, 0.0099, -0.2543)
MSTRG.24920.1	down	0.0120	-1.549	VWA2 (down, 0.0389, -0.2320), ZNF165 (up, 0.0284, 0.2438), MARCKSL1 (up, 0.0218, 0.1985), E2F3 (up, 0.0218, 0.1952), KIF13A (down, 0.0131, -0.1706), PITX2 (down, 0.0453, -0.1976), SNRPN (down, 0.016, -0.1981), MEIS3 (down, 0.0218, -0.2535), PTPRN2 (down, 0.0163, -0.2716), TPPP3 (down, 0.0084, -0.2747)
MSTRG.25096.9	up	0.0002	5.373	NELL2 (up, 0.0426, 0.2278)
MSTRG.25102.1	up	0.0383	1.484	FAM111B (up, 0.0007, 0.3640), CD40 (up, 0.0437, 0.2030), LOC102184252 (up, 0.0069, 0.2972), MMP12 (up, 0.0016, 0.2779), LOC102174470 (up, 0.0021, 0.2683), PTGS2 (up, 0.0081, 0.2594), SLC16A1 (up, 0.0401, 0.2321), KCNJ15 (up, 0.0286, 0.2298), UAP1 (up, 0.0394, 0.2023)
MSTRG.25231.2	down	0.0373	-2.370	LOC100861174 (down, 0.0467, -0.2150), LOC108634363 (down, 0.0361, -0.1015)
MSTRG.25997.1	up	0.0074	2.798	BIRC5 (up, 0.0088, 0.2550), RALGPS2 (up, 0.0216, 0.1380), KLHL29 (up, 0.0013, 0.2747), PTCH2 (up, 0.0088, 0.1971), MTHFD1 (up, 0.0114, 0.1881), MRPS27 (up, 0.0254, 0.1649), TMEM87A (up, 0.0164, 0.1548), PITX2 (down, 0.0453, -0.1976), SNRPN (down, 0.016, -0.1981), SIDT2 (down, 0.0011, -0.2037), LOC106503362 (down, 0.0208, -0.2331), MEIS3 (down, 0.0218, -0.2535), C2CD2L (down, 0.0163, -0.2549)
MSTRG.26033.2	up	0.0109	1.848	TLR1 (up, 0.007, 0.2740), LOC108638522 (up, 0.0374, 0.1840), WRN (up, 0.0188, 0.1520), Capra_hircus_newGene_18572 (up, 0.0001, 0.4124), Capra_hircus_newGene_21230 (up, 0.0007, 0.3532), SCN8A (up, 0.0164, 0.2530), PREB (up, 0.0229, 0.1666), CASP3 (up, 0.0416, 0.1591), MATR3 (up, 0.0168, 0.1024), DNASE1L2 (down, 0.0235, -0.2394)
MSTRG.26129.4	down	0.0308	-2.093	TMTC1 (down, 0.0149, -0.1880), CABYR (up, 0.0000, 0.5116), UGGT2 (up, 0.027, 0.2456), ZNF165 (up, 0.0284, 0.2438), NET1 (down, 0.0475, -0.1192), SP2 (down, 0.0328, -0.1892)
MSTRG.26212.1	up	0.0162	1.872	GLI1 (up, 0.0045, 0.2678), RPE (up, 0.0204, 0.1995), SHTN1 (up, 0.0417, 0.1655), STIM1 (down, 0.0186, -0.1401), PPP1R7 (down, 0.0455, -0.1478), PITX2 (down, 0.0453, -0.1976), MEIS3 (down, 0.0218, -0.2535), AADACL3 (down, 0.0000, -0.4595)
MSTRG.26212.2	down	0.0000	-9.343	LOC102177708 (down, 0.0034, -0.3300), ATP13A4 (up, 0.0000, 0.5377), C1H3orf33 (up, 0.0077, 0.3012), ZNF165 (up, 0.0284, 0.2438), C14H8orf82 (up, 0.0302, 0.2351), SHTN1 (up, 0.0417, 0.1655), TMEM87A (up, 0.0164, 0.1548), PPP1R7 (down, 0.0455, -0.1478), PITX2

DE ncRNA	Regu lated	P value	log2Fold Change	Target mRNA (Regulated, P value, log2Fold Change)
				(down, 0.0453, -0.1976), RNASEH2C (down, 0.0152, -0.2724), RFX2 (down, 0.0003, -0.4097), AADACL3 (down, 0.0000, -0.4595)
MSTRG.26 322.32	up	0.0022	4.310	Capra_hircus_newGene_4795 (up, 0.0034, 0.2777), KLHL29 (up, 0.0013, 0.2747), PRSS12 (up, 0.0286, 0.2448), LRP2 (up, 0.0105, 0.2375), PXYLP1 (up, 0.034, 0.2322), EPPK1 (up, 0.012, 0.2178), COL5A1 (down, 0.0315, -0.2375)
MSTRG.26 332.3	up	0.0077	3.578	STEAP2 (up, 0.0332, 0.2200), Capra_hircus_newGene_21365 (up, 0.0024, 0.3424), KLHL29 (up, 0.0013, 0.2747)
MSTRG.26 340.2	up	0.0061	2.523	UROS (up, 0.0032, 0.3268), LOC102184252 (up, 0.0069, 0.2972), CD200 (up, 0.0069, 0.2948), LOC102174470 (up, 0.0021, 0.2683), PVR (up, 0.0254, 0.2467), STAT4 (up, 0.0143, 0.2454), FASTKD2 (up, 0.0031, 0.2242), PREB (up, 0.0229, 0.1666), AKAP17A (down, 0.0305, -0.1924), CD247 (down, 0.0151, -0.2586)
MSTRG.26 353.4	up	0.0006	3.029	Capra_hircus_newGene_9735 (up, 0.0153, 0.2735), LOC102172474 (up, 0.0379, 0.2348), LOC102173111 (up, 0.0403, 0.2312), ZBTB6 (down, 0.0223, -0.1686), Capra_hircus_newGene_12918 (down, 0.0262, -0.2107)
MSTRG.26 5.11	up	0.0143	2.995	TMEM221 (up, 0.0000, 0.3318), Capra_hircus_newGene_4795 (up, 0.0034, 0.2777), ITGA2 (up, 0.0262, 0.2254), EPPK1 (up, 0.012, 0.2178), MLX (up, 0.0488, 0.1363), LOC108637962 (up, 0.0491, 0.1324)
MSTRG.26 566.9	up	0.0149	3.078	Capra_hircus_newGene_11982 (up, 0.0073, 0.3031), LOC102172474 (up, 0.0379, 0.2348), LOC102179921 (down, 0.0066, -0.2763)
MSTRG.26 665.1	down	0.0246	-2.035	LOC102185150 (down, 0.0323, -0.1983), TRPM6 (down, 0.044, -0.2277)
MSTRG.26 68.12	down	0.0207	-2.136	TMTC1 (down, 0.0149, -0.1880), CABYR (up, 0.0000, 0.5116), KRT7 (up, 0.0017, 0.3227), UGGT2 (up, 0.027, 0.2456), ZNF165 (up, 0.0284, 0.2438), LOC102179192 (up, 0.0383, 0.2335), C25H16orf59 (up, 0.0398, 0.2287), MARCKSL1 (up, 0.0218, 0.1985), TXNDC5 (up, 0.0176, 0.1726), IDH3A (up, 0.0434, 0.1643), CTU1 (down, 0.0105, -0.1511), KIF13A (down, 0.0131, -0.1706), SP2 (down, 0.0328, -0.1892)
MSTRG.26 900.1	down	0.0355	-1.939	LOC102177708 (down, 0.0034, -0.3300), MEF2D (down, 0.0383, -0.1429), LOC102185708 (down, 0.0452, -0.1771), ATP8B2 (down, 0.0088, -0.1796), CORIN (down, 0.01, -0.2553)
MSTRG.27 028.13	up	0.0000	4.846	MYO3B (up, 0.0075, 0.3008), Capra_hircus_newGene_9735 (up, 0.0153, 0.2735), MRPS18B (up, 0.0189, 0.2197), MTHFD1 (up, 0.0114, 0.1881), C8H9orf72 (up, 0.0236, 0.1854), MRPS27 (up, 0.0254, 0.1649), ZBTB6 (down, 0.0223, -0.1686), PRKD1 (down, 0.0013, -0.3107)
MSTRG.27 028.8	up	0.0040	2.829	ZNF260 (up, 0.0000, 0.4445), RBM3 (up, 0.0002, 0.3306), Capra_hircus_newGene_11982 (up, 0.0073, 0.3031), LOC106503901 (up, 0.0144, 0.2708), RASSF8 (up, 0.0095, 0.2249), NUP54 (up, 0.0119, 0.1988), ASB3 (up, 0.0331, 0.1443), LOC102179921 (down, 0.0066, -0.2763)

DE ncRNA	Regulated	P value	log2Fold Change	Target mRNA (Regulated, P value, log2Fold Change)
MSTRG.27029.1	down	0.0385	-1.458	EHD2 (down, 0.0092, -0.2020), CCDC22 (down, 0.0389, -0.1870), SLC4A7 (up, 0.009, 0.2394), NCSTN (down, 0.0307, -0.1114), MRPL47 (down, 0.0424, -0.1918), GFAP (down, 0.0251, -0.2006), MEX3B (down, 0.0251, -0.2489), CDKN1C (down, 0.0268, -0.2505), LOC102189713 (down, 0.0178, -0.2676), SERTM1 (down, 0.0056, -0.3107)
MSTRG.27055.1	down	0.0247	-1.492	SLC39A3 (down, 0.0162, -0.2300), SOX18 (down, 0.0463, -0.2230), EHD2 (down, 0.0092, -0.2020), CCDC22 (down, 0.0389, -0.1870), PNPLA6 (down, 0.0213, -0.1405), PLAC9 (down, 0.0092, -0.2905), CLMN (down, 0.0039, -0.3259)
MSTRG.27074.4	down	0.0415	-2.019	FAM169B (down, 0.047, -0.2005), TPPP3 (down, 0.0084, -0.2747)
MSTRG.27125.3	up	0.0140	2.662	TLR1 (up, 0.007, 0.2740), SGPP1 (up, 0.0111, 0.2210), ATP13A4 (up, 0.0000, 0.5377), ZNF260 (up, 0.0000, 0.4445), RBM3 (up, 0.0002, 0.3306), KLHL29 (up, 0.0013, 0.2747), ZNF165 (up, 0.0284, 0.2438), LOC102182782 (up, 0.0312, 0.2428), EID1 (up, 0.0235, 0.1828), Capra_hircus_newGene_5067 (down, 0.0193, -0.1314), RNASEH2C (down, 0.0152, -0.2724)
MSTRG.27382.1	up	0.0252	1.954	PNPT1 (up, 0.0173, 0.1880), GRM2 (up, 0.0089, 0.2625), HS6ST3 (up, 0.0216, 0.2028)
MSTRG.27601.1	up	0.0195	2.310	ATP13A4 (up, 0.0000, 0.5377), Capra_hircus_newGene_22856 (up, 0.0029, 0.3335), C1H3orf33 (up, 0.0077, 0.3012), KCND2 (up, 0.0141, 0.2751), LOC102191766 (up, 0.0171, 0.2697), PEX12 (up, 0.032, 0.2129), DCK (up, 0.034, 0.1895), CCDC50 (up, 0.0212, 0.1801), ARHGEF1 (down, 0.041, -0.1163), DCTN2 (down, 0.0161, -0.1403), BCL9L (down, 0.0444, -0.1451), CARD10 (down, 0.0396, -0.1942), SIDT2 (down, 0.0011, -0.2037), Capra_hircus_newGene_3630 (down, 0.0351, -0.2138), FZD2 (down, 0.0263, -0.2244), LOC106503362 (down, 0.0208, -0.2331), CD247 (down, 0.0151, -0.2586)
MSTRG.27606.14	up	0.0005	2.471	FAM111B (up, 0.0007, 0.3640), CXCR2 (up, 0.0253, 0.2180), UROS (up, 0.0032, 0.3268), KCNJ15 (up, 0.0286, 0.2298), LOC102172005 (up, 0.0281, 0.0851)
MSTRG.27629.1	up	0.0133	2.859	FMO4 (up, 0.006, 0.3088), Capra_hircus_newGene_9735 (up, 0.0153, 0.2735)
MSTRG.27688.2	down	0.0294	-1.630	CMYA5 (up, 0.0425, 0.1700), SLC5A6 (up, 0.001, 0.2799), KCND2 (up, 0.0141, 0.2751), NELL2 (up, 0.0426, 0.2278), MRPS18B (up, 0.0189, 0.2197), TMEM87A (up, 0.0164, 0.1548), DCTN2 (down, 0.0161, -0.1403), GAS7 (down, 0.009, -0.1735), UTP14A (down, 0.0054, -0.1862), SLC7A7 (down, 0.0239, -0.2352), DZIP1L (down, 0.0009, -0.3689)
MSTRG.27830.1	down	0.0327	-1.906	Capra_hircus_newGene_18403 (down, 0.0486, -0.2210), FARSB (up, 0.049, 0.1350), LOC102175781 (up, 0.0338, 0.2392), USP3 (up, 0.042, 0.1117)

DE ncRNA	Regulated	P value	log2Fold Change	Target mRNA (Regulated, P value, log2Fold Change)
MSTRG.2784.1	down	0.0043	-2.901	CNR1 (down, 0.0117, -0.2250)
MSTRG.27929.3	up	0.0107	2.564	STEAP2 (up, 0.0332, 0.2200), SPTA1 (up, 0.0153, 0.2057), CHST2 (up, 0.0049, 0.1866), HS3ST1 (up, 0.0332, 0.1818)
MSTRG.27970.1	down	0.0467	-1.533	CC2D1B (up, 0.0303, 0.1309), SIPA1L2 (down, 0.0443, -0.1743), EMILIN2 (down, 0.0412, -0.2227), C1QC (down, 0.041, -0.2304), VIM (down, 0.0049, -0.2610)
MSTRG.28026.3	down	0.0138	-2.529	SPAG1 (up, 0.003, 0.2879), TSR3 (up, 0.0188, 0.2326), SLC31A1 (up, 0.0102, 0.1874), CCDC50 (up, 0.0212, 0.1801), FAM13B (up, 0.0243, 0.1423), SRSF3 (up, 0.0396, 0.1062), LOC102174081 (down, 0.0163, -0.2513)
MSTRG.28026.4	up	0.0069	3.659	TMEM221 (up, 0.0000, 0.3318), Capra_hircus_newGene_4795 (up, 0.0034, 0.2777), PRSS12 (up, 0.0286, 0.2448), PXYLP1 (up, 0.034, 0.2322), MLX (up, 0.0488, 0.1363), COL5A1 (down, 0.0315, -0.2375)
MSTRG.28095.1	up	0.0000	5.871	WRN (up, 0.0188, 0.1520), ATP13A4 (up, 0.0000, 0.5377), LOC108635404 (up, 0.0002, 0.3433), LOC108635390 (up, 0.0014, 0.3178), TSR3 (up, 0.0188, 0.2326), FASTKD2 (up, 0.0031, 0.2242), DDX52 (up, 0.0099, 0.2227), C1D (up, 0.0062, 0.1999), DCK (up, 0.034, 0.1895), CXADR (up, 0.009, 0.1649), PPP1R7 (down, 0.0455, -0.1478), STMN3 (down, 0.0379, -0.1576), DYRK1B (down, 0.0187, -0.1879), CERCAM (down, 0.0179, -0.2276), LOC106503362 (down, 0.0208, -0.2331), SOX4 (down, 0.0000, -1.1673)
MSTRG.28215.24	down	0.0272	-2.052	NUCB1 (down, 0.0184, -0.2330), Capra_hircus_newGene_15790 (up, 0.0496, 0.2215), ADIPOR2 (down, 0.0441, -0.1419), SLC9A3R2 (down, 0.0483, -0.1615), PTRF (down, 0.0451, -0.1989), GFAP (down, 0.0251, -0.2006), BICDL2 (down, 0.0357, -0.2026), RAB3IL1 (down, 0.0492, -0.2194), FBLN5 (down, 0.0366, -0.2349), LHX6 (down, 0.0316, -0.2381)
MSTRG.28478.2	up	0.0276	2.409	TNFRSF18 (up, 0.0027, 0.3213), KY (up, 0.0109, 0.2881), Capra_hircus_newGene_6138 (up, 0.0057, 0.2692), APLN (up, 0.0178, 0.2470), CCDC66 (up, 0.0438, 0.2116), AMZ2 (up, 0.0447, 0.1973), TMEM123 (up, 0.0093, 0.1929), TRMT10C (up, 0.0383, 0.1858), SLC5A8 (down, 0.0181, -0.2358)
MSTRG.28589.3	down	0.0256	-1.935	CCDC22 (down, 0.0389, -0.1870), RNF217 (down, 0.0289, -0.1620), Capra_hircus_newGene_1952 (up, 0.0029, 0.2627), PVR (up, 0.0254, 0.2467), GMFB (up, 0.0287, 0.1858), PNPLA6 (down, 0.0213, -0.1405)
MSTRG.28656.4	down	0.0486	-2.149	FUCA1 (down, 0.0231, -0.2070), OS9 (down, 0.037, -0.1297), ADIPOR2 (down, 0.0441, -0.1419), MAP3K3 (down, 0.0342, -0.1613), TOX2 (down, 0.0444, -0.1819), TEF (down, 0.0243, -0.1869), BICDL2 (down, 0.0357, -0.2026), FBLN5 (down, 0.0366, -0.2349), GPR153 (down, 0.0185, -0.2531), TSPAN33 (down, 0.0138, -0.2583), STC2 (down, 0.0053, -0.3048)

DE ncRNA	Regulated	P value	log2Fold Change	Target mRNA (Regulated, P value, log2Fold Change)
MSTRG.2866.1	down	0.0076	-2.083	NME7 (up, 0.0047, 0.2694), PUS7 (up, 0.013, 0.2169), CDC40 (up, 0.0031, 0.2084), LOC102169288 (up, 0.03, 0.2069), CXADR (up, 0.009, 0.1649), CC2D1B (up, 0.0303, 0.1309), CLCC1 (down, 0.0063, -0.2214), GLT8D2 (down, 0.0368, -0.2283), VIM (down, 0.0049, -0.2610), PRKCDBP (down, 0.0011, -0.3262)
MSTRG.28754.1	down	0.0047	-2.307	TRIM9 (down, 0.0324, -0.2330), Capra_hircus_newGene_18572 (up, 0.0001, 0.4124), Capra_hircus_newGene_12502 (up, 0.0035, 0.2992), CDC40 (up, 0.0031, 0.2084), DDX47 (up, 0.0314, 0.1675), CXADR (up, 0.009, 0.1649), Capra_hircus_newGene_9779 (up, 0.0406, 0.1587), UTP14A (down, 0.0054, -0.1862), FBXL22 (down, 0.0168, -0.2050), ENGASE (down, 0.0116, -0.2122), CLCC1 (down, 0.0063, -0.2214), SNX24 (down, 0.0081, -0.2668), PRKCDBP (down, 0.0011, -0.3262)
MSTRG.28870.6	up	0.0351	1.236	ZNF605 (up, 0.0085, 0.2370), COPE (down, 0.0237, -0.2160), Capra_hircus_newGene_18572 (up, 0.0001, 0.4124), Capra_hircus_newGene_22856 (up, 0.0029, 0.3335), UROS (up, 0.0032, 0.3268), CD200 (up, 0.0069, 0.2948), LOC102174470 (up, 0.0021, 0.2683), CD48 (up, 0.0242, 0.2537), SCN8A (up, 0.0164, 0.2530), STAT4 (up, 0.0143, 0.2454), SLC4A7 (up, 0.009, 0.2394), DCK (up, 0.034, 0.1895), PREB (up, 0.0229, 0.1666), CASP3 (up, 0.0416, 0.1591), RASA1 (up, 0.0378, 0.1485), SEPN1 (down, 0.0023, -0.2478), SOX4 (down, 0.0000, -1.1673)
MSTRG.29051.31	up	0.0328	1.781	MRPS18B (up, 0.0189, 0.2197), TBX1 (up, 0.0236, 0.2136), C8H9orf72 (up, 0.0236, 0.1854), PLEC (down, 0.0334, -0.1588), ZBTB6 (down, 0.0223, -0.1686), GAS7 (down, 0.009, -0.1735), RUBCN (down, 0.0015, -0.1962), PRKD1 (down, 0.0013, -0.3107)
MSTRG.29114.1	down	0.0037	-4.592	LOC102176218 (down, 0.0131, -0.2770), HSPA2 (down, 0.033, -0.2230), LOC100861174 (down, 0.0467, -0.2150), PUS7 (up, 0.013, 0.2169), METTL17 (up, 0.0256, 0.1940), LOC108636556 (down, 0.0465, -0.2001), LOC102187755 (down, 0.0004, -0.3721)
MSTRG.29417.1	down	0.0182	-2.396	COG1 (down, 0.0003, -0.3120), BIRC5 (up, 0.0088, 0.2550), CNR1 (down, 0.0117, -0.2250), SPAG1 (up, 0.003, 0.2879), DPP6 (up, 0.033, 0.2408), SLC35G1 (up, 0.0253, 0.2343), E2F3 (up, 0.0218, 0.1952), RET (up, 0.0156, 0.1862), SNRPN (down, 0.016, -0.1981), TRPM6 (down, 0.044, -0.2277), PRUNE2 (down, 0.025, -0.2336), YPEL1 (down, 0.0203, -0.2455), RYR2 (down, 0.0101, -0.2808), RHOB (down, 0.0039, -0.3015)
MSTRG.2959.2	down	0.0401	-1.888	CMYA5 (up, 0.0425, 0.1700), KCND2 (up, 0.0141, 0.2751), LOC102191766 (up, 0.0171, 0.2697), NEK6 (up, 0.0273, 0.2275), DCTN2 (down, 0.0161, -0.1403), CARD10 (down, 0.0396, -0.1942), CFP (down, 0.0253, -0.2026)
MSTRG.2959.3	up	0.0000	4.365	ATP13A4 (up, 0.0000, 0.5377), Capra_hircus_newGene_18572 (up, 0.0001, 0.4124), C1H3orf33 (up, 0.0077, 0.3012), KCND2 (up, 0.0141,

DE ncRNA	Regulated	P value	log2Fold Change	Target mRNA (Regulated, P value, log2Fold Change)
				0.2751), LOC102191766 (up, 0.0171, 0.2697), FASTKD2 (up, 0.0031, 0.2242), PEX12 (up, 0.032, 0.2129), DCK (up, 0.034, 0.1895), CCDC50 (up, 0.0212, 0.1801), ARHGEF1 (down, 0.041, -0.1163), PPP1R7 (down, 0.0455, -0.1478), SIDT2 (down, 0.0011, -0.2037), Capra_hircus_newGene_3630 (down, 0.0351, -0.2138), LOC106503362 (down, 0.0208, -0.2331), CD247 (down, 0.0151, -0.2586)
MSTRG.29777.1	down	0.0023	-2.767	LOC106503979 (down, 0.0184, -0.2280), CNR1 (down, 0.0117, -0.2250), FGD6 (up, 0.0034, 0.2506), DPP6 (up, 0.033, 0.2408), IDH3A (up, 0.0434, 0.1643), TRPM6 (down, 0.044, -0.2277), YPEL1 (down, 0.0203, -0.2455)
MSTRG.30256.4	up	0.0050	3.116	ZNF260 (up, 0.0000, 0.4445), RBM3 (up, 0.0002, 0.3306), LOC106503901 (up, 0.0144, 0.2708), RAB37 (up, 0.013, 0.2690), TARS2 (up, 0.0062, 0.2366), RASSF8 (up, 0.0095, 0.2249), PLEKHH2 (up, 0.0076, 0.2063), SLC25A13 (up, 0.0276, 0.2062), FSD1L (up, 0.0364, 0.2032), NUP54 (up, 0.0119, 0.1988), EID1 (up, 0.0235, 0.1828), CDCA7L (up, 0.015, 0.1647), DHX36 (up, 0.0096, 0.1475), ASB3 (up, 0.0331, 0.1443), TOX2 (down, 0.0444, -0.1819), PPP2R2B (down, 0.0363, -0.2234), RNASEH2C (down, 0.0152, -0.2724), LOC102179921 (down, 0.0066, -0.2763)
MSTRG.30390.48	down	0.0166	-2.454	LOC102168573 (up, 0.0023, 0.2800), RALGPS2 (up, 0.0216, 0.1380), KRT7 (up, 0.0017, 0.3227), LOC102174170 (up, 0.0065, 0.3036), DTD2 (up, 0.0089, 0.2513), LOC102169288 (up, 0.03, 0.2069), NUP54 (up, 0.0119, 0.1988), MTHFD1 (up, 0.0114, 0.1881), RET (up, 0.0156, 0.1862), MRPS27 (up, 0.0254, 0.1649)
MSTRG.30390.49	up	0.0000	7.400	FAM206A (up, 0.0444, 0.1890), ZNF260 (up, 0.0000, 0.4445), RBM3 (up, 0.0002, 0.3306), Capra_hircus_newGene_11982 (up, 0.0073, 0.3031), MAT1A (up, 0.0042, 0.2817), DTD2 (up, 0.0089, 0.2513), TAF1D (up, 0.0177, 0.2439), TARS2 (up, 0.0062, 0.2366), PLEKHH2 (up, 0.0076, 0.2063), FSD1L (up, 0.0364, 0.2032), NUP54 (up, 0.0119, 0.1988), TRMT13 (up, 0.0348, 0.1971), ASB3 (up, 0.0331, 0.1443), LOC102179921 (down, 0.0066, -0.2763)
MSTRG.30494.1	up	0.0039	2.551	ZNF605 (up, 0.0085, 0.2370), ATP13A4 (up, 0.0000, 0.5377), Capra_hircus_newGene_18572 (up, 0.0001, 0.4124), Capra_hircus_newGene_22856 (up, 0.0029, 0.3335), UROS (up, 0.0032, 0.3268), C1H3orf33 (up, 0.0077, 0.3012), CD200 (up, 0.0069, 0.2948), KCND2 (up, 0.0141, 0.2751), LOC102174470 (up, 0.0021, 0.2683), STAT4 (up, 0.0143, 0.2454), DCK (up, 0.034, 0.1895), PREB (up, 0.0229, 0.1666), TMEM87A (up, 0.0164, 0.1548), ARHGEF1 (down, 0.041, -0.1163), DCTN2 (down, 0.0161, -0.1403), PPP1R7 (down, 0.0455, -0.1478), CARD10 (down, 0.0396, -0.1942), CD247 (down, 0.0151, -0.2586), AADACL3 (down, 0.0000, -0.4595), SOX4 (down, 0.0000, -1.1673)

DE ncRNA	Regulated	P value	log2Fold Change	Target mRNA (Regulated, P value, log2Fold Change)
MSTRG.3054.13	up	0.0111	2.241	MMP13 (up, 0.048, 0.1680), Capra_hircus_newGene_4795 (up, 0.0034, 0.2777), ITGA2 (up, 0.0262, 0.2254), LOC108637962 (up, 0.0491, 0.1324)
MSTRG.30579.1	up	0.0063	2.990	ZNF605 (up, 0.0085, 0.2370), WRN (up, 0.0188, 0.1520), ATP13A4 (up, 0.0000, 0.5377), Capra_hircus_newGene_18572 (up, 0.0001, 0.4124), Capra_hircus_newGene_2167 (up, 0.0015, 0.3595), Capra_hircus_newGene_22856 (up, 0.0029, 0.3335), UROS (up, 0.0032, 0.3268), C1H3orf33 (up, 0.0077, 0.3012), KCND2 (up, 0.0141, 0.2751), LOC102191766 (up, 0.0171, 0.2697), DCK (up, 0.034, 0.1895), RASA1 (up, 0.0378, 0.1485), ARHGEF1 (down, 0.041, -0.1163), DCTN2 (down, 0.0161, -0.1403), PPP1R7 (down, 0.0455, -0.1478), CARD10 (down, 0.0396, -0.1942), CD247 (down, 0.0151, -0.2586), DZIP1L (down, 0.0009, -0.3689), AADACL3 (down, 0.0000, -0.4595)
MSTRG.30697.1	up	0.0369	1.389	COPE (down, 0.0237, -0.2160), Capra_hircus_newGene_22856 (up, 0.0029, 0.3335), UROS (up, 0.0032, 0.3268), SCN8A (up, 0.0164, 0.2530), SLC4A7 (up, 0.009, 0.2394), DCK (up, 0.034, 0.1895), LOC102172005 (up, 0.0281, 0.0851), STMN3 (down, 0.0379, -0.1576), HSPA12B (down, 0.0426, -0.2022)
MSTRG.30716.2	up	0.0470	1.550	PNPT1 (up, 0.0173, 0.1880), MARCH7 (up, 0.0025, 0.1770), TMEM221 (up, 0.0000, 0.3318), Capra_hircus_newGene_5523 (up, 0.0105, 0.2529), BZW2 (up, 0.017, 0.2133), LDAH (up, 0.0313, 0.1579), MLX (up, 0.0488, 0.1363), THSD7A (down, 0.0294, -0.2382), SCPEP1 (down, 0.0054, -0.2938), RHOB (down, 0.0039, -0.3015)
MSTRG.31258.3	up	0.0231	2.527	CXCR2 (up, 0.0253, 0.2180), FABP5 (up, 0.0455, 0.2080), SPTA1 (up, 0.0153, 0.2057), LOC102172005 (up, 0.0281, 0.0851)
MSTRG.31459.1	down	0.0258	-1.780	COG1 (down, 0.0003, -0.3120), BIRC5 (up, 0.0088, 0.2550), SLC35G1 (up, 0.0253, 0.2343), ABCE1 (up, 0.0424, 0.1445)
MSTRG.31743.12	down	0.0235	-2.451	FUCA1 (down, 0.0231, -0.2070), CANT1 (down, 0.0341, -0.1450), Capra_hircus_newGene_5769 (up, 0.0088, 0.2831), CAPNS1 (down, 0.0452, -0.1094), CALM1 (down, 0.0373, -0.1231), GRK2 (down, 0.0118, -0.1519), PPM1A (down, 0.0219, -0.1521), MAP3K3 (down, 0.0342, -0.1613), TOX2 (down, 0.0444, -0.1819), MEX3B (down, 0.0251, -0.2489), GPR153 (down, 0.0185, -0.2531), STC2 (down, 0.0053, -0.3048)
MSTRG.32131.2	down	0.0093	-1.969	PLEC (down, 0.0334, -0.1588), FHL5 (down, 0.032, -0.2352), YPEL1 (down, 0.0203, -0.2455)
MSTRG.3250.1	down	0.0321	-2.548	IRF2BPL (up, 0.0029, 0.3350), Capra_hircus_newGene_18403 (down, 0.0486, -0.2210), Capra_hircus_newGene_9779 (up, 0.0406, 0.1587), UTP6 (up, 0.0467, 0.1493), ENGASE (down, 0.0116, -0.2122)
MSTRG.33107.2	up	0.0075	2.957	LOC108635404 (up, 0.0002, 0.3433), LOC108633201 (up, 0.0009, 0.3354), Capra_hircus_newGene_1504 (up, 0.0118, 0.2767), CA4 (up, 0.0203, 0.2598), LOC102176870 (up, 0.0088, 0.2564), DDX52 (up, 0.0099,

DE ncRNA	Regu lated	P value	log2Fold Change	Target mRNA (Regulated, P value, log2Fold Change)
				0.2227), FAM13B (up, 0.0243, 0.1423), STMN3 (down, 0.0379, -0.1576), CERCAM (down, 0.0179, -0.2276), LSAMP (down, 0.0286, -0.2352)
MSTRG.33 372.1	down	0.0256	-2.820	CNR1 (down, 0.0117, -0.2250), SHC3 (down, 0.0455, -0.2059), THAP8 (down, 0.0472, -0.2128), TRPM6 (down, 0.044, -0.2277), PRUNE2 (down, 0.025, -0.2336)
MSTRG.33 407.1	up	0.0263	1.435	LOC108638522 (up, 0.0374, 0.1840), LOC102169889 (up, 0.0335, 0.2391), PLEKHH2 (up, 0.0076, 0.2063)
MSTRG.33 425.1	up	0.0042	2.348	ZNF605 (up, 0.0085, 0.2370), Capra_hircus_newGene_22856 (up, 0.0029, 0.3335), UROS (up, 0.0032, 0.3268), LOC102184252 (up, 0.0069, 0.2972), CD200 (up, 0.0069, 0.2948), MMP12 (up, 0.0016, 0.2779), LOC102174470 (up, 0.0021, 0.2683), CD48 (up, 0.0242, 0.2537), PVR (up, 0.0254, 0.2467), STAT4 (up, 0.0143, 0.2454), LOC102169889 (up, 0.0335, 0.2391), UAP1 (up, 0.0394, 0.2023), PREB (up, 0.0229, 0.1666), CASP3 (up, 0.0416, 0.1591), BCL9L (down, 0.0444, -0.1451), AKAP17A (down, 0.0305, -0.1924), CD247 (down, 0.0151, -0.2586)
MSTRG.33 462.1	up	0.0281	2.201	LOC108634682 (up, 0.0499, 0.1580), LOC102179921 (down, 0.0066, -0.2763)
MSTRG.33 620.4	down	0.0028	-3.631	WRN (up, 0.0188, 0.1520), ATP13A4 (up, 0.0000, 0.5377), C1H3orf33 (up, 0.0077, 0.3012), FASTKD2 (up, 0.0031, 0.2242), C1D (up, 0.0062, 0.1999), RPE (up, 0.0204, 0.1995), DCK (up, 0.034, 0.1895), TMEM87A (up, 0.0164, 0.1548), PPP1R7 (down, 0.0455, -0.1478), AIP (down, 0.0216, -0.1957), PITX2 (down, 0.0453, -0.1976), LOC106503362 (down, 0.0208, -0.2331), AADACL3 (down, 0.0000, -0.4595), SOX4 (down, 0.0000, -1.1673)
MSTRG.33 96.1	up	0.0053	1.980	FAM206A (up, 0.0444, 0.1890), ZNF260 (up, 0.0000, 0.4445), RAB37 (up, 0.013, 0.2690), DTD2 (up, 0.0089, 0.2513), GLMN (up, 0.0137, 0.2399), C7H5orf45 (up, 0.0301, 0.2384), TARS2 (up, 0.0062, 0.2366), RASSF8 (up, 0.0095, 0.2249), FSD1L (up, 0.0364, 0.2032), NUP54 (up, 0.0119, 0.1988), MARCKSL1 (up, 0.0218, 0.1985), TRMT13 (up, 0.0348, 0.1971), CDCA7L (up, 0.015, 0.1647), ASB3 (up, 0.0331, 0.1443), SLC25A19 (down, 0.049, -0.1966), CEP89 (down, 0.0356, -0.2204), PPP2R2B (down, 0.0363, -0.2234), LOC102179921 (down, 0.0066, -0.2763)
MSTRG.34 031.1	down	0.0001	-5.678	COG1 (down, 0.0003, -0.3120), LOC106503979 (down, 0.0184, -0.2280), CNR1 (down, 0.0117, -0.2250), DPP6 (up, 0.033, 0.2408), ABCE1 (up, 0.0424, 0.1445)
MSTRG.34 056.1	down	0.0005	-3.523	CMYA5 (up, 0.0425, 0.1700), WRN (up, 0.0188, 0.1520), ATP13A4 (up, 0.0000, 0.5377), KCND2 (up, 0.0141, 0.2751), PPAT (up, 0.0255, 0.2475), DDX52 (up, 0.0099, 0.2227), DCK (up, 0.034, 0.1895), TRAPPC13 (up, 0.0458, 0.1645), CFP (down, 0.0253, -0.2026), PMM1 (down, 0.0377, -0.2179), SLC7A7 (down, 0.0239, -0.2352), OTOGL (down, 0.0094, -0.2432), CD248 (down, 0.0042, -0.3239), DZIP1L (down, 0.0009, -0.3689)

DE ncRNA	Regulated	P value	log2Fold Change	Target mRNA (Regulated, P value, log2Fold Change)
MSTRG.34104.1	up	0.0014	3.445	CXCR2 (up, 0.0253, 0.2180), WRN (up, 0.0188, 0.1520), LOC108635404 (up, 0.0002, 0.3433), Capra_hircus_newGene_22856 (up, 0.0029, 0.3335), UROS (up, 0.0032, 0.3268), SCN8A (up, 0.0164, 0.2530), DDX52 (up, 0.0099, 0.2227), DCK (up, 0.034, 0.1895), LOC102172005 (up, 0.0281, 0.0851), STMN3 (down, 0.0379, -0.1576)
MSTRG.34512.7	up	0.0106	2.527	MARCH7 (up, 0.0025, 0.1770), LOC108635404 (up, 0.0002, 0.3433), TMEM221 (up, 0.0000, 0.3318), LOC108635390 (up, 0.0014, 0.3178), SPAG1 (up, 0.003, 0.2879), KLHL29 (up, 0.0013, 0.2747), DDX52 (up, 0.0099, 0.2227), FAM13B (up, 0.0243, 0.1423), MLX (up, 0.0488, 0.1363), CERCAM (down, 0.0179, -0.2276), LSAMP (down, 0.0286, -0.2352), LOC108638594 (down, 0.0155, -0.2654)
MSTRG.3479.2	down	0.0024	-3.116	RALGPS2 (up, 0.0216, 0.1380), ZNF260 (up, 0.0000, 0.4445), LOC102174170 (up, 0.0065, 0.3036), CMC4 (up, 0.0124, 0.2814), KLHL29 (up, 0.0013, 0.2747), ZNF165 (up, 0.0284, 0.2438), DPP6 (up, 0.033, 0.2408), RET (up, 0.0156, 0.1862), LOC108634770 (down, 0.037, -0.2143), SCPEP1 (down, 0.0054, -0.2938)
MSTRG.34920.1	up	0.0216	2.343	BIRC5 (up, 0.0088, 0.2550), LRP2 (up, 0.0105, 0.2375), PXYLP1 (up, 0.034, 0.2322), SMOC2 (up, 0.0285, 0.2199), PIK3CB (up, 0.0082, 0.1974), PTCH2 (up, 0.0088, 0.1971), WNT5A (up, 0.0247, 0.1768), MEIS3 (down, 0.0218, -0.2535), C2CD2L (down, 0.0163, -0.2549), SCPEP1 (down, 0.0054, -0.2938)
MSTRG.35080.2	down	0.0005	-2.984	SGPP1 (up, 0.0111, 0.2210), ATP13A4 (up, 0.0000, 0.5377), C1H3orf33 (up, 0.0077, 0.3012), KLHL29 (up, 0.0013, 0.2747), LOC102176870 (up, 0.0088, 0.2564), Capra_hircus_newGene_5067 (down, 0.0193, -0.1314), PITX2 (down, 0.0453, -0.1976), RNASEH2C (down, 0.0152, -0.2724), RFX2 (down, 0.0003, -0.4097)
MSTRG.35211.17	up	0.0071	1.849	ATP13A4 (up, 0.0000, 0.5377), CD200 (up, 0.0069, 0.2948), KY (up, 0.0109, 0.2881), CD48 (up, 0.0242, 0.2537), LOC102186466 (up, 0.0416, 0.2137), Capra_hircus_newGene_10897 (up, 0.044, 0.2049), TMEM123 (up, 0.0093, 0.1929), DCK (up, 0.034, 0.1895), PREB (up, 0.0229, 0.1666), CASP3 (up, 0.0416, 0.1591), AKAP17A (down, 0.0305, -0.1924), THBS2 (down, 0.008, -0.2837)
MSTRG.35259.1	down	0.0366	-2.874	SLC25A19 (down, 0.049, -0.1966), GDA (down, 0.0318, -0.2011), CD248 (down, 0.0042, -0.3239), ANAPC13 (down, 0.0167, -0.2574)
MSTRG.35284.4	down	0.0000	-11.310	LOC102177708 (down, 0.0034, -0.3300), ATP13A4 (up, 0.0000, 0.5377), UGGT2 (up, 0.027, 0.2456), ZNF165 (up, 0.0284, 0.2438), C14H8orf82 (up, 0.0302, 0.2351), SHTN1 (up, 0.0417, 0.1655), NET1 (down, 0.0475, -0.1192), ATP8B2 (down, 0.0088, -0.1796), PITX2 (down, 0.0453, -0.1976), RNASEH2C (down, 0.0152, -0.2724), RFX2 (down, 0.0003, -0.4097), AADACL3 (down, 0.0000, -0.4595)

DE ncRNA	Regulated	P value	log2Fold Change	Target mRNA (Regulated, P value, log2Fold Change)
MSTRG.35421.1	down	0.0282	-1.747	LOC102177708 (down, 0.0034, -0.3300), MEF2D (down, 0.0383, -0.1429), LOC102185708 (down, 0.0452, -0.1771), FZD2 (down, 0.0263, -0.2244), DDAH2 (down, 0.0099, -0.2543)
MSTRG.35591.10	down	0.0500	-2.155	CCDC22 (down, 0.0389, -0.1870), Capra_hircus_newGene_1952 (up, 0.0029, 0.2627), PNPLA6 (down, 0.0213, -0.1405), CAV1 (down, 0.0218, -0.1919), FHL5 (down, 0.032, -0.2352), YPEL1 (down, 0.0203, -0.2455)
MSTRG.35656.1	down	0.0367	-2.335	LOC102185150 (down, 0.0323, -0.1983)
MSTRG.35664.1	up	0.0092	2.193	FAM206A (up, 0.0444, 0.1890), RBM3 (up, 0.0002, 0.3306), Capra_hircus_newGene_9422 (up, 0.0058, 0.3034), MAT1A (up, 0.0042, 0.2817), TARM1 (up, 0.0096, 0.2777), DTD2 (up, 0.0089, 0.2513), TAF1D (up, 0.0177, 0.2439), TARS2 (up, 0.0062, 0.2366), TSTD3 (up, 0.0406, 0.2313), LOC106503969 (up, 0.0224, 0.2105), FSD1L (up, 0.0364, 0.2032), NUP54 (up, 0.0119, 0.1988), TRMT13 (up, 0.0348, 0.1971), LOC106503943 (up, 0.0485, 0.1846), ASMTL (down, 0.0037, -0.2855)
MSTRG.35666.11	up	0.0172	3.509	LOC108634682 (up, 0.0499, 0.1580)
MSTRG.35666.3	up	0.0000	4.980	LOC102168573 (up, 0.0023, 0.2800), FAM206A (up, 0.0444, 0.1890), KRT7 (up, 0.0017, 0.3227), FMO4 (up, 0.006, 0.3088), DTD2 (up, 0.0089, 0.2513), TARS2 (up, 0.0062, 0.2366), SLC25A13 (up, 0.0276, 0.2062), FSD1L (up, 0.0364, 0.2032), NUP54 (up, 0.0119, 0.1988)
MSTRG.35724.1	up	0.0477	1.185	TLR1 (up, 0.007, 0.2740), Capra_hircus_newGene_10117 (up, 0.0368, 0.2150), ATP13A4 (up, 0.0000, 0.5377), UROS (up, 0.0032, 0.3268), C1H3orf33 (up, 0.0077, 0.3012), CD200 (up, 0.0069, 0.2948), CD48 (up, 0.0242, 0.2537), LOC102169889 (up, 0.0335, 0.2391), LOC102186466 (up, 0.0416, 0.2137), PLEKHH2 (up, 0.0076, 0.2063), TMEM123 (up, 0.0093, 0.1929), PREB (up, 0.0229, 0.1666), CASP3 (up, 0.0416, 0.1591), BCL9L (down, 0.0444, -0.1451), AKAP17A (down, 0.0305, -0.1924)
MSTRG.36094.1	down	0.0302	-1.788	TLR1 (up, 0.007, 0.2740), MYO3B (up, 0.0075, 0.3008), SLC5A6 (up, 0.001, 0.2799), MRPS18B (up, 0.0189, 0.2197), MTHFD1 (up, 0.0114, 0.1881), C8H9orf72 (up, 0.0236, 0.1854), SNURF (up, 0.0418, 0.1668), MRPS27 (up, 0.0254, 0.1649), TMEM87A (up, 0.0164, 0.1548), BCLAF1 (up, 0.0065, 0.1472), AWAT1 (down, 0.0158, -0.2730)
MSTRG.36276.1	up	0.0306	1.743	FAM206A (up, 0.0444, 0.1890), TARM1 (up, 0.0096, 0.2777), GLMN (up, 0.0137, 0.2399), TRMT13 (up, 0.0348, 0.1971), TRMT10C (up, 0.0383, 0.1858), CDCA7L (up, 0.015, 0.1647), ASB3 (up, 0.0331, 0.1443), GRK2 (down, 0.0118, -0.1519), AIP (down, 0.0216, -0.1957), SLC25A19 (down, 0.049, -0.1966), LOC102179921 (down, 0.0066, -0.2763)
MSTRG.36438.1	up	0.0159	2.742	Capra_hircus_newGene_18572 (up, 0.0001, 0.4124), SLC5A6 (up, 0.001, 0.2799), Capra_hircus_newGene_17456 (up, 0.0337, 0.2401), FASTKD2 (up, 0.0031, 0.2242), PUS7 (up, 0.013, 0.2169), CDC40 (up, 0.0031,

DE ncRNA	Regu lated	P value	log2Fold Change	Target mRNA (Regulated, P value, log2Fold Change)
				0.2084), NUP54 (up, 0.0119, 0.1988), METTL17 (up, 0.0256, 0.1940), DDX47 (up, 0.0314, 0.1675), CXADR (up, 0.009, 0.1649), BCLAF1 (up, 0.0065, 0.1472), UTP14A (down, 0.0054, -0.1862), ENGASE (down, 0.0116, -0.2122), CLCC1 (down, 0.0063, -0.2214), SNX24 (down, 0.0081, -0.2668), SOX4 (down, 0.0000, -1.1673)
MSTRG.36464.1	down	0.0241	-1.287	COG1 (down, 0.0003, -0.3120), NEK6 (up, 0.0273, 0.2275), LOC102173997 (up, 0.0248, 0.2154), PEX12 (up, 0.032, 0.2129), CCDC50 (up, 0.0212, 0.1801), TMEM87A (up, 0.0164, 0.1548), ARHGEF1 (down, 0.041, -0.1163), SNRPN (down, 0.016, -0.1981), SIDT2 (down, 0.0011, -0.2037), LOC106503362 (down, 0.0208, -0.2331), TPPP3 (down, 0.0084, -0.2747)
MSTRG.36464.2	up	0.0446	1.295	NELL2 (up, 0.0426, 0.2278), SLIT1 (up, 0.0482, 0.1882), Capra_hircus_newGene_14773 (up, 0.0472, 0.0905)
MSTRG.36843.2	up	0.0351	1.707	AMZ2 (up, 0.0447, 0.1973), IDS (down, 0.0246, -0.1402), STMN3 (down, 0.0379, -0.1576), DYRK1B (down, 0.0187, -0.1879), SP2 (down, 0.0328, -0.1892), HSPA12B (down, 0.0426, -0.2022), RP2 (down, 0.0474, -0.2078), SLC5A8 (down, 0.0181, -0.2358), CDKN1C (down, 0.0268, -0.2505)
MSTRG.36911.4	up	0.0097	2.198	FAM111B (up, 0.0007, 0.3640), CXCR2 (up, 0.0253, 0.2180), CD40 (up, 0.0437, 0.2030), UROS (up, 0.0032, 0.3268), MMP12 (up, 0.0016, 0.2779), PTGS2 (up, 0.0081, 0.2594), KCNJ15 (up, 0.0286, 0.2298), SPTA1 (up, 0.0153, 0.2057), LOC102172005 (up, 0.0281, 0.0851)
MSTRG.37179.1	up	0.0488	2.434	IRF2BPL (up, 0.0029, 0.3350), ARFIP1 (down, 0.0054, -0.2133)
MSTRG.37574.4	up	0.0384	1.814	RNF217 (down, 0.0289, -0.1620), Capra_hircus_newGene_11982 (up, 0.0073, 0.3031), LOC102172474 (up, 0.0379, 0.2348), Capra_hircus_newGene_11916 (down, 0.035, -0.1415), KLF4 (down, 0.0257, -0.1428), PLEC (down, 0.0334, -0.1588), ZBTB6 (down, 0.0223, -0.1686)
MSTRG.37730.1	up	0.0372	2.661	Capra_hircus_newGene_11982 (up, 0.0073, 0.3031), NELL2 (up, 0.0426, 0.2278)
MSTRG.37758.22	up	0.0066	4.406	Capra_hircus_newGene_11982 (up, 0.0073, 0.3031), LOC102169889 (up, 0.0335, 0.2391), LOC106503943 (up, 0.0485, 0.1846), LOC102173760 (up, 0.0397, 0.1705)
MSTRG.37910.1	up	0.0336	2.131	LOC102168573 (up, 0.0023, 0.2800), RNF217 (down, 0.0289, -0.1620), DTD2 (up, 0.0089, 0.2513), LOC102172474 (up, 0.0379, 0.2348), KLF4 (down, 0.0257, -0.1428), ZBTB6 (down, 0.0223, -0.1686), RUBCN (down, 0.0015, -0.1962)
MSTRG.37929.1	up	0.0441	2.702	Capra_hircus_newGene_9735 (up, 0.0153, 0.2735), CROT (up, 0.0482, 0.2166), CIDEA (down, 0.0015, -0.3405)
MSTRG.37932.5	up	0.0178	1.734	MYO3B (up, 0.0075, 0.3008), CROT (up, 0.0482, 0.2166), MRPS27 (up, 0.0254, 0.1649), Capra_hircus_newGene_14773 (up, 0.0472, 0.0905)

DE ncRNA	Regulated	P value	log2Fold Change	Target mRNA (Regulated, P value, log2Fold Change)
MSTRG.37955.1	up	0.0096	2.542	LOC102188618 (up, 0.0315, 0.2370), LOC102191766 (up, 0.0171, 0.2697), MRPS18B (up, 0.0189, 0.2197), MTHFD1 (up, 0.0114, 0.1881), C8H9orf72 (up, 0.0236, 0.1854), OPA1 (up, 0.0123, 0.1729), TMEM87A (up, 0.0164, 0.1548), STIM1 (down, 0.0186, -0.1401), DCTN2 (down, 0.0161, -0.1403), GAS7 (down, 0.009, -0.1735), CFP (down, 0.0253, -0.2026), SLC7A7 (down, 0.0239, -0.2352), AADACL3 (down, 0.0000, -0.4595)
MSTRG.38161.1	up	0.0366	2.248	LOC102191766 (up, 0.0171, 0.2697), NELL2 (up, 0.0426, 0.2278), SLIT1 (up, 0.0482, 0.1882), DCTN2 (down, 0.0161, -0.1403)
MSTRG.38671.22	down	0.0200	-3.091	ADIPOR2 (down, 0.0441, -0.1419), SLC9A3R2 (down, 0.0483, -0.1615), PTRF (down, 0.0451, -0.1989), RAB3IL1 (down, 0.0492, -0.2194), FBLN5 (down, 0.0366, -0.2349)
MSTRG.38671.24	up	0.0288	2.644	LOC108634682 (up, 0.0499, 0.1580)
MSTRG.38671.28	up	0.0188	3.245	Capra_hircus_newGene_14773 (up, 0.0472, 0.0905)
MSTRG.38775.38	up	0.0469	1.178	ZNF605 (up, 0.0085, 0.2370), TRIM9 (down, 0.0324, -0.2330), COPE (down, 0.0237, -0.2160), Capra_hircus_newGene_18572 (up, 0.0001, 0.4124), Capra_hircus_newGene_2167 (up, 0.0015, 0.3595), Capra_hircus_newGene_22856 (up, 0.0029, 0.3335), KCND2 (up, 0.0141, 0.2751), LOC102174470 (up, 0.0021, 0.2683), STAT4 (up, 0.0143, 0.2454), DCK (up, 0.034, 0.1895), UTP14A (down, 0.0054, -0.1862), MRGPRF (down, 0.0001, -0.4224)
MSTRG.38812.22	down	0.0065	-2.129	FUCA1 (down, 0.0231, -0.2070), MEF2D (down, 0.0383, -0.1429), ALKBH5 (down, 0.0214, -0.1488), ATP8B2 (down, 0.0088, -0.1796), TOX2 (down, 0.0444, -0.1819), TEF (down, 0.0243, -0.1869), RBPMS2 (down, 0.0458, -0.2247), GPR153 (down, 0.0185, -0.2531), DDAH2 (down, 0.0099, -0.2543), CORIN (down, 0.01, -0.2553), STC2 (down, 0.0053, -0.3048)
MSTRG.38981.1	down	0.0110	-1.626	ZNRF2 (up, 0.0382, 0.1620), ATP13A4 (up, 0.0000, 0.5377), Capra_hircus_newGene_18572 (up, 0.0001, 0.4124), Capra_hircus_newGene_1952 (up, 0.0029, 0.2627), FGD6 (up, 0.0034, 0.2506), Capra_hircus_newGene_17456 (up, 0.0337, 0.2401), TSR3 (up, 0.0188, 0.2326), FASTKD2 (up, 0.0031, 0.2242), CDC40 (up, 0.0031, 0.2084), CXADR (up, 0.009, 0.1649), PPP1R7 (down, 0.0455, -0.1478), LOC106503362 (down, 0.0208, -0.2331), SNX24 (down, 0.0081, -0.2668), SOX4 (down, 0.0000, -1.1673)
MSTRG.39287.1	up	0.0000	6.332	PNPT1 (up, 0.0173, 0.1880), MARCH7 (up, 0.0025, 0.1770), TMEM221 (up, 0.0000, 0.3318), Capra_hircus_newGene_4795 (up, 0.0034, 0.2777), GRM2 (up, 0.0089, 0.2625), ITGA2 (up, 0.0262, 0.2254), C1D (up, 0.0062,

DE ncRNA	Regulated	P value	log2Fold Change	Target mRNA (Regulated, P value, log2Fold Change)
				0.1999), MLX (up, 0.0488, 0.1363), SP2 (down, 0.0328, -0.1892), ENPEP (down, 0.0054, -0.2921)
MSTRG.39377.36	up	0.0453	1.406	MMP13 (up, 0.048, 0.1680), IDS (down, 0.0246, -0.1402), STMN3 (down, 0.0379, -0.1576), DYRK1B (down, 0.0187, -0.1879), HSPA12B (down, 0.0426, -0.2022), RP2 (down, 0.0474, -0.2078), CERCAM (down, 0.0179, -0.2276)
MSTRG.39377.43	up	0.0479	2.757	LOC102178853 (up, 0.0085, 0.2790), CXCR2 (up, 0.0253, 0.2180), AMD1 (up, 0.0459, 0.1660), Capra_hircus_newGene_13914 (up, 0.0000, 0.4721), LOC108633201 (up, 0.0009, 0.3354), Capra_hircus_newGene_1504 (up, 0.0118, 0.2767)
MSTRG.39493.1	down	0.0156	-1.523	MARCKSL1 (up, 0.0218, 0.1985), E2F3 (up, 0.0218, 0.1952), Capra_hircus_newGene_11916 (down, 0.035, -0.1415), KIF13A (down, 0.0131, -0.1706), SNRPN (down, 0.016, -0.1981), PTPRN2 (down, 0.0163, -0.2716), TPPP3 (down, 0.0084, -0.2747)
MSTRG.39564.8	up	0.0443	2.855	Capra_hircus_newGene_9735 (up, 0.0153, 0.2735)
MSTRG.39780.1	up	0.0452	1.332	ZNF260 (up, 0.0000, 0.4445), RBM3 (up, 0.0002, 0.3306), MAT1A (up, 0.0042, 0.2817), LOC106503901 (up, 0.0144, 0.2708), RAB37 (up, 0.013, 0.2690), RASSF8 (up, 0.0095, 0.2249), PLEKHH2 (up, 0.0076, 0.2063), NUP54 (up, 0.0119, 0.1988), TRMT13 (up, 0.0348, 0.1971), NMD3 (up, 0.0038, 0.1877), CDCA7L (up, 0.015, 0.1647), DHX36 (up, 0.0096, 0.1475), ASB3 (up, 0.0331, 0.1443), CEP89 (down, 0.0356, -0.2204), PPP2R2B (down, 0.0363, -0.2234)
MSTRG.39798.3	up	0.0049	2.201	KRT7 (up, 0.0017, 0.3227), FMO4 (up, 0.006, 0.3088), DTD2 (up, 0.0089, 0.2513), TBX1 (up, 0.0236, 0.2136), PEX12 (up, 0.032, 0.2129), LOC102169288 (up, 0.03, 0.2069), ALG2 (down, 0.034, -0.1464), RUBCN (down, 0.0015, -0.1962), CIDEA (down, 0.0015, -0.3405)
MSTRG.4004.17	up	0.0007	4.874	Capra_hircus_newGene_13914 (up, 0.0000, 0.4721), LOC108633201 (up, 0.0009, 0.3354), TNFRSF18 (up, 0.0027, 0.3213), Capra_hircus_newGene_1504 (up, 0.0118, 0.2767), APLN (up, 0.0178, 0.2470), HS6ST3 (up, 0.0216, 0.2028), AMZ2 (up, 0.0447, 0.1973)
MSTRG.40093.2	down	0.0264	-1.664	Capra_hircus_newGene_12502 (up, 0.0035, 0.2992), FHL5 (down, 0.032, -0.2352)
MSTRG.40268.2	up	0.0166	2.129	TARS2 (up, 0.0062, 0.2366), RASSF8 (up, 0.0095, 0.2249), SLC25A13 (up, 0.0276, 0.2062), FSD1L (up, 0.0364, 0.2032), NUP54 (up, 0.0119, 0.1988)
MSTRG.40315.1	down	0.0215	-1.860	ATP13A4 (up, 0.0000, 0.5377), C1H3orf33 (up, 0.0077, 0.3012), CD200 (up, 0.0069, 0.2948), LOC102186466 (up, 0.0416, 0.2137), TRAPPC13 (up, 0.0458, 0.1645), CASP3 (up, 0.0416, 0.1591), BCL9L (down, 0.0444, -0.1451), PTMS (down, 0.0402, -0.1617), FZD2 (down, 0.0263, -0.2244)

DE ncRNA	Regulated	P value	log2Fold Change	Target mRNA (Regulated, P value, log2Fold Change)
MSTRG.40406.1	down	0.0230	-1.852	LOC102183952 (up, 0.0381, 0.2228), ABCE1 (up, 0.0424, 0.1445)
MSTRG.40421.13	down	0.0120	-1.771	CCDC22 (down, 0.0389, -0.1870), RNF217 (down, 0.0289, -0.1620), Capra_hircus_newGene_1952 (up, 0.0029, 0.2627), FGD6 (up, 0.0034, 0.2506), PVR (up, 0.0254, 0.2467), Capra_hircus_newGene_17456 (up, 0.0337, 0.2401), FASTKD2 (up, 0.0031, 0.2242), CDC40 (up, 0.0031, 0.2084), GMFB (up, 0.0287, 0.1858), PNPLA6 (down, 0.0213, -0.1405)
MSTRG.40421.14	up	0.0000	10.821	TLR1 (up, 0.007, 0.2740), ATP13A4 (up, 0.0000, 0.5377), Capra_hircus_newGene_11982 (up, 0.0073, 0.3031), FASTKD2 (up, 0.0031, 0.2242), CDC40 (up, 0.0031, 0.2084), PLEKHH2 (up, 0.0076, 0.2063), BCLAF1 (up, 0.0065, 0.1472), SOX4 (down, 0.0000, -1.1673)
MSTRG.40422.1	down	0.0347	-1.432	CAPNS1 (down, 0.0452, -0.1094), LOC102185708 (down, 0.0452, -0.1771), GPR153 (down, 0.0185, -0.2531), STC2 (down, 0.0053, -0.3048)
MSTRG.40437.9	up	0.0000	6.619	ATP13A4 (up, 0.0000, 0.5377), FASTKD2 (up, 0.0031, 0.2242), CDC40 (up, 0.0031, 0.2084), LOC102169288 (up, 0.03, 0.2069), MTHFD1 (up, 0.0114, 0.1881), CXADR (up, 0.009, 0.1649), MRPS27 (up, 0.0254, 0.1649), TMEM87A (up, 0.0164, 0.1548), PPP1R7 (down, 0.0455, -0.1478), ZBTB6 (down, 0.0223, -0.1686), RUBCN (down, 0.0015, -0.1962), PITX2 (down, 0.0453, -0.1976), Capra_hircus_newGene_12918 (down, 0.0262, -0.2107), LOC106503362 (down, 0.0208, -0.2331), PRKD1 (down, 0.0013, -0.3107), SOX4 (down, 0.0000, -1.1673)
MSTRG.40477.1	up	0.0126	2.490	IRF2BPL (up, 0.0029, 0.3350), TRIM9 (down, 0.0324, -0.2330), Capra_hircus_newGene_18572 (up, 0.0001, 0.4124), SLC5A6 (up, 0.001, 0.2799), CDC40 (up, 0.0031, 0.2084), DDX47 (up, 0.0314, 0.1675), CXADR (up, 0.009, 0.1649), UTP14A (down, 0.0054, -0.1862), ENGASE (down, 0.0116, -0.2122), SNX24 (down, 0.0081, -0.2668)
MSTRG.40629.2	down	0.0274	-1.609	CDC40 (up, 0.0031, 0.2084), PNPLA6 (down, 0.0213, -0.1405)
MSTRG.40738.3	up	0.0003	4.075	FAM111B (up, 0.0007, 0.3640), STEAP2 (up, 0.0332, 0.2200), CD40 (up, 0.0437, 0.2030), Capra_hircus_newGene_22856 (up, 0.0029, 0.3335), UROS (up, 0.0032, 0.3268), LOC102184252 (up, 0.0069, 0.2972), MMP12 (up, 0.0016, 0.2779), LOC102174470 (up, 0.0021, 0.2683), PTGS2 (up, 0.0081, 0.2594), STAT4 (up, 0.0143, 0.2454), KCNJ15 (up, 0.0286, 0.2298), UAP1 (up, 0.0394, 0.2023), CHST2 (up, 0.0049, 0.1866), HS3ST1 (up, 0.0332, 0.1818), PREB (up, 0.0229, 0.1666)
MSTRG.40775.1	down	0.0321	-1.770	LOC102168573 (up, 0.0023, 0.2800), LOC106503979 (down, 0.0184, -0.2280), FGD6 (up, 0.0034, 0.2506), SRXN1 (up, 0.0278, 0.2185)
MSTRG.41103.4	down	0.0009	-4.417	PAPD4 (up, 0.0085, 0.1790), LOC102182782 (up, 0.0312, 0.2428), DPP6 (up, 0.033, 0.2408), LOC102183952 (up, 0.0381, 0.2228), NMD3 (up, 0.0038, 0.1877), USP3 (up, 0.042, 0.1117), Capra_hircus_newGene_21088 (down, 0.0183, -0.1713)

DE ncRNA	Regulated	P value	log2Fold Change	Target mRNA (Regulated, P value, log2Fold Change)
MSTRG.41295.1	up	0.0409	1.288	TNFRSF18 (up, 0.0027, 0.3213), KY (up, 0.0109, 0.2881), Capra_hircus_newGene_1504 (up, 0.0118, 0.2767), Capra_hircus_newGene_6138 (up, 0.0057, 0.2692), APLN (up, 0.0178, 0.2470), AMZ2 (up, 0.0447, 0.1973)
MSTRG.41305.2	up	0.0220	2.700	LOC102191766 (up, 0.0171, 0.2697), Capra_hircus_newGene_2169 (up, 0.0211, 0.2286), CFP (down, 0.0253, -0.2026), Capra_hircus_newGene_3630 (down, 0.0351, -0.2138)
MSTRG.41396.10	down	0.0180	-2.111	NEK6 (up, 0.0273, 0.2275), LOC102173997 (up, 0.0248, 0.2154), PEX12 (up, 0.032, 0.2129), Capra_hircus_newGene_5067 (down, 0.0193, -0.1314), Capra_hircus_newGene_3416 (down, 0.0015, -0.3259)
MSTRG.41579.6	down	0.0115	-2.711	POU2F1 (up, 0.0325, 0.1270), DIO2 (up, 0.0195, 0.2441)
MSTRG.41706.1	down	0.0419	-2.564	LOC102185708 (down, 0.0452, -0.1771), MN1 (down, 0.0145, -0.2759)
MSTRG.41810.1	up	0.0333	1.339	ATP13A4 (up, 0.0000, 0.5377), KLHL29 (up, 0.0013, 0.2747), ZNF165 (up, 0.0284, 0.2438), TXNDC5 (up, 0.0176, 0.1726), PPP1R7 (down, 0.0455, -0.1478), PITX2 (down, 0.0453, -0.1976), SNRPN (down, 0.016, -0.1981), LOC106503362 (down, 0.0208, -0.2331), SOX4 (down, 0.0000, -1.1673)
MSTRG.41892.1	down	0.0024	-2.348	TRAPPC13 (up, 0.0458, 0.1645), PMM1 (down, 0.0377, -0.2179), STC2 (down, 0.0053, -0.3048)
MSTRG.42054.1	up	0.0201	2.561	FAM111B (up, 0.0007, 0.3640), CD40 (up, 0.0437, 0.2030), Capra_hircus_newGene_22856 (up, 0.0029, 0.3335), UROS (up, 0.0032, 0.3268), CD200 (up, 0.0069, 0.2948), MMP12 (up, 0.0016, 0.2779), LOC102174470 (up, 0.0021, 0.2683), PTGS2 (up, 0.0081, 0.2594), CD48 (up, 0.0242, 0.2537), STAT4 (up, 0.0143, 0.2454), SLC4A7 (up, 0.009, 0.2394), SLC16A1 (up, 0.0401, 0.2321), KCNJ15 (up, 0.0286, 0.2298), SPTA1 (up, 0.0153, 0.2057), PREB (up, 0.0229, 0.1666), BCL9L (down, 0.0444, -0.1451)
MSTRG.4208.4	down	0.0171	-1.851	FUCA1 (down, 0.0231, -0.2070), EHD2 (down, 0.0092, -0.2020), Capra_hircus_newGene_5769 (up, 0.0088, 0.2831), CAPNS1 (down, 0.0452, -0.1094), OS9 (down, 0.037, -0.1297), ALKBH5 (down, 0.0214, -0.1488), PPM1A (down, 0.0219, -0.1521), MAP3K3 (down, 0.0342, -0.1613), TOX2 (down, 0.0444, -0.1819), TEF (down, 0.0243, -0.1869), ZBTB47 (down, 0.0011, -0.2435), STC2 (down, 0.0053, -0.3048), SEPTIN6 (down, 0.0016, -0.3371)
MSTRG.42545.1	up	0.0016	3.185	FAM111B (up, 0.0007, 0.3640), CXCR2 (up, 0.0253, 0.2180), CD40 (up, 0.0437, 0.2030), UROS (up, 0.0032, 0.3268), PTGS2 (up, 0.0081, 0.2594), KCNJ15 (up, 0.0286, 0.2298), SPTA1 (up, 0.0153, 0.2057), LOC102172005 (up, 0.0281, 0.0851)

DE ncRNA	Regulated	P value	log2Fold Change	Target mRNA (Regulated, P value, log2Fold Change)
MSTRG.42720.20	down	0.0171	-1.399	HSPA2 (down, 0.033, -0.2230), NME7 (up, 0.0047, 0.2694), PUS7 (up, 0.013, 0.2169), METTL17 (up, 0.0256, 0.1940), UTP14A (down, 0.0054, -0.1862), GLT8D2 (down, 0.0368, -0.2283), OTOGL (down, 0.0094, -0.2432), DZIP1L (down, 0.0009, -0.3689), LOC102187755 (down, 0.0004, -0.3721)
MSTRG.42819.3	down	0.0084	-2.280	IRF2BPL (up, 0.0029, 0.3350), LOC100861174 (down, 0.0467, -0.2150), SPAG1 (up, 0.003, 0.2879), NME7 (up, 0.0047, 0.2694), METTL17 (up, 0.0256, 0.1940), USP3 (up, 0.042, 0.1117), SRSF3 (up, 0.0396, 0.1062), ENGASE (down, 0.0116, -0.2122), LOC102174081 (down, 0.0163, -0.2513)
MSTRG.42891.11	up	0.0429	1.351	MAT1A (up, 0.0042, 0.2817), LOC106503969 (up, 0.0224, 0.2105), LOC106503943 (up, 0.0485, 0.1846)
MSTRG.4315.36	up	0.0076	5.165	LOC108635404 (up, 0.0002, 0.3433), LOC108635390 (up, 0.0014, 0.3178), LOC102172005 (up, 0.0281, 0.0851), STMN3 (down, 0.0379, -0.1576), CERCAM (down, 0.0179, -0.2276)
MSTRG.43260.4	up	0.0497	1.431	Capra_hircus_newGene_9735 (up, 0.0153, 0.2735), LOC102172474 (up, 0.0379, 0.2348), Capra_hircus_newGene_12918 (down, 0.0262, -0.2107)
MSTRG.43331.19	down	0.0442	-1.846	COG1 (down, 0.0003, -0.3120), LOC102168852 (up, 0.0003, 0.3332), CCDC112 (up, 0.0468, 0.2230), FYTTD1 (up, 0.0129, 0.1452), ABCE1 (up, 0.0424, 0.1445)
MSTRG.4343.1	up	0.0077	2.337	NELL2 (up, 0.0426, 0.2278), MRPS18B (up, 0.0189, 0.2197), SLIT1 (up, 0.0482, 0.1882), Capra_hircus_newGene_14773 (up, 0.0472, 0.0905), STIM1 (down, 0.0186, -0.1401), MEIS3 (down, 0.0218, -0.2535)
MSTRG.43654.18	down	0.0269	-2.101	LOC106503362 (down, 0.0208, -0.2331)
MSTRG.43654.7	up	0.0136	3.265	Capra_hircus_newGene_1504 (up, 0.0118, 0.2767), AMZ2 (up, 0.0447, 0.1973), STMN3 (down, 0.0379, -0.1576), LOC106503362 (down, 0.0208, -0.2331)
MSTRG.43801.5	up	0.0000	7.704	MYO3B (up, 0.0075, 0.3008), NELL2 (up, 0.0426, 0.2278), MRPS18B (up, 0.0189, 0.2197), SLIT1 (up, 0.0482, 0.1882), Capra_hircus_newGene_14773 (up, 0.0472, 0.0905), SNX33 (down, 0.0234, -0.1527), MEIS3 (down, 0.0218, -0.2535)
MSTRG.43931.5	up	0.0134	2.058	IRF2BPL (up, 0.0029, 0.3350), LOC102176218 (down, 0.0131, -0.2770), TRIM9 (down, 0.0324, -0.2330), CMYA5 (up, 0.0425, 0.1700), WRN (up, 0.0188, 0.1520), Capra_hircus_newGene_18572 (up, 0.0001, 0.4124), SLC5A6 (up, 0.001, 0.2799), FASTKD2 (up, 0.0031, 0.2242), CDC40 (up, 0.0031, 0.2084), CCDC50 (up, 0.0212, 0.1801), DDX47 (up, 0.0314, 0.1675), CXADR (up, 0.009, 0.1649), UTP6 (up, 0.0467, 0.1493), SRSF3 (up, 0.0396, 0.1062), UTP14A (down, 0.0054, -0.1862), ENGASE (down, 0.0116, -0.2122), SNX24 (down, 0.0081, -0.2668), LOC102187755 (down, 0.0004, -0.3721)

DE ncRNA	Regu lated	P value	log2Fold Change	Target mRNA (Regulated, P value, log2Fold Change)
MSTRG.4416.2	up	0.0413	1.936	Capra_hircus_newGene_12726 (up, 0.0159, 0.2591), GLMN (up, 0.0137, 0.2399), TRMT13 (up, 0.0348, 0.1971), SLC25A19 (down, 0.049, -0.1966), LOC102179921 (down, 0.0066, -0.2763)
MSTRG.44267.4	down	0.0026	-1.937	TLR1 (up, 0.007, 0.2740), ATP13A4 (up, 0.0000, 0.5377), ZNF165 (up, 0.0284, 0.2438), C14H8orf82 (up, 0.0302, 0.2351), MTHFD1 (up, 0.0114, 0.1881), C8H9orf72 (up, 0.0236, 0.1854), TMEM87A (up, 0.0164, 0.1548), ASB3 (up, 0.0331, 0.1443), PPP1R7 (down, 0.0455, -0.1478), ZBTB6 (down, 0.0223, -0.1686), SLC25A19 (down, 0.049, -0.1966), PITX2 (down, 0.0453, -0.1976), RNASEH2C (down, 0.0152, -0.2724), LOC102179921 (down, 0.0066, -0.2763), SOX4 (down, 0.0000, -1.1673)
MSTRG.44435.11	up	0.0375	2.926	LOC102169889 (up, 0.0335, 0.2391), PLEKHH2 (up, 0.0076, 0.2063), LOC102173760 (up, 0.0397, 0.1705)
MSTRG.44524.4	up	0.0161	3.394	FMO4 (up, 0.006, 0.3088), CROT (up, 0.0482, 0.2166), PPP1R13B (down, 0.012, -0.2041), CIDEA (down, 0.0015, -0.3405)
MSTRG.4465.3	up	0.0110	2.561	Capra_hircus_newGene_9735 (up, 0.0153, 0.2735), C8H9orf72 (up, 0.0236, 0.1854), ZBTB6 (down, 0.0223, -0.1686), Capra_hircus_newGene_12918 (down, 0.0262, -0.2107)
MSTRG.44954.1	up	0.0402	1.483	MARCH7 (up, 0.0025, 0.1770), WRN (up, 0.0188, 0.1520), ATP13A4 (up, 0.0000, 0.5377), KLHL29 (up, 0.0013, 0.2747), NME7 (up, 0.0047, 0.2694), GLI1 (up, 0.0045, 0.2678), PPAT (up, 0.0255, 0.2475), C1D (up, 0.0062, 0.1999), PIK3CB (up, 0.0082, 0.1974), MTHFD1 (up, 0.0114, 0.1881), MRPS27 (up, 0.0254, 0.1649), OTOGL (down, 0.0094, -0.2432)
MSTRG.45196.1	down	0.0336	-1.903	RALGPS2 (up, 0.0216, 0.1380), Capra_hircus_newGene_9735 (up, 0.0153, 0.2735), TARS2 (up, 0.0062, 0.2366)
MSTRG.45203.1	down	0.0052	-3.179	TLR1 (up, 0.007, 0.2740), WRN (up, 0.0188, 0.1520), ATP13A4 (up, 0.0000, 0.5377), Capra_hircus_newGene_18572 (up, 0.0001, 0.4124), C1H3orf33 (up, 0.0077, 0.3012), SLC5A6 (up, 0.001, 0.2799), MRPS18B (up, 0.0189, 0.2197), CDC40 (up, 0.0031, 0.2084), DCK (up, 0.034, 0.1895), MTHFD1 (up, 0.0114, 0.1881), CDCA7L (up, 0.015, 0.1647), TMEM87A (up, 0.0164, 0.1548), BCLAF1 (up, 0.0065, 0.1472), UTP14A (down, 0.0054, -0.1862), SOX4 (down, 0.0000, -1.1673)
MSTRG.45882.2	down	0.0418	-2.837	LOC102177708 (down, 0.0034, -0.3300), FZD8 (up, 0.0352, 0.1967), SHTN1 (up, 0.0417, 0.1655)
MSTRG.46127.1	up	0.0430	2.035	CD200 (up, 0.0069, 0.2948), CD48 (up, 0.0242, 0.2537), LOC102186466 (up, 0.0416, 0.2137), TMEM123 (up, 0.0093, 0.1929), CASP3 (up, 0.0416, 0.1591), AKAP17A (down, 0.0305, -0.1924), THBS2 (down, 0.008, -0.2837)
MSTRG.46148.2	up	0.0293	1.630	MYL9 (down, 0.0263, -0.2260), LOC102172005 (up, 0.0281, 0.0851), STMN3 (down, 0.0379, -0.1576)
MSTRG.46161.2	down	0.0000	-10.732	KRT36 (down, 0.0001, -0.4450), ADAM22 (down, 0.0158, -0.2070), EHD2 (down, 0.0092, -0.2020), CCDC22 (down, 0.0389, -0.1870),

DE ncRNA	Regulated	P value	log2Fold Change	Target mRNA (Regulated, P value, log2Fold Change)
				Capra_hircus_newGene_1952 (up, 0.0029, 0.2627), Capra_hircus_newGene_17456 (up, 0.0337, 0.2401), PNPLA6 (down, 0.0213, -0.1405), TSC1 (down, 0.0098, -0.1441), SNX33 (down, 0.0234, -0.1527), SIPA1L2 (down, 0.0443, -0.1743), CAV1 (down, 0.0218, -0.1919), RGAG4 (down, 0.0143, -0.2037), ARFIP1 (down, 0.0054, -0.2133), CLCC1 (down, 0.0063, -0.2214), DACT1 (down, 0.0422, -0.2224), FHL5 (down, 0.032, -0.2352), CAMK4 (down, 0.0332, -0.2359), YPEL1 (down, 0.0203, -0.2455), LOC102189713 (down, 0.0178, -0.2676), MXD4 (down, 0.0019, -0.2706), ANGPTL1 (down, 0.0101, -0.2857), CDH13 (down, 0.0049, -0.2895), PRKCDBP (down, 0.0011, -0.3262), SEPTIN6 (down, 0.0016, -0.3371), MRGPRF (down, 0.0001, -0.4224)
MSTRG.46180.3	up	0.0416	2.861	STEAP2 (up, 0.0332, 0.2200), SPTA1 (up, 0.0153, 0.2057)
MSTRG.46255.1	up	0.0101	2.259	MMP13 (up, 0.048, 0.1680), LOC108635404 (up, 0.0002, 0.3433), LOC108635390 (up, 0.0014, 0.3178), LOC102176870 (up, 0.0088, 0.2564), STMN3 (down, 0.0379, -0.1576), CERCAM (down, 0.0179, -0.2276), LSAMP (down, 0.0286, -0.2352), LOC108638594 (down, 0.0155, -0.2654)
MSTRG.46267.1	up	0.0001	3.721	TLR1 (up, 0.007, 0.2740), Capra_hircus_newGene_11982 (up, 0.0073, 0.3031), CDC40 (up, 0.0031, 0.2084), PLEKHH2 (up, 0.0076, 0.2063), NUP54 (up, 0.0119, 0.1988), DHX36 (up, 0.0096, 0.1475), BCLAF1 (up, 0.0065, 0.1472), MATR3 (up, 0.0168, 0.1024)
MSTRG.46477.1	down	0.0146	-2.338	LOC102177708 (down, 0.0034, -0.3300), FZD8 (up, 0.0352, 0.1967), NET1 (down, 0.0475, -0.1192), LOC102185708 (down, 0.0452, -0.1771), ATP8B2 (down, 0.0088, -0.1796), CORIN (down, 0.01, -0.2553)
MSTRG.46598.1	up	0.0025	3.097	PNPT1 (up, 0.0173, 0.1880), MARCH7 (up, 0.0025, 0.1770), TMEM221 (up, 0.0000, 0.3318), Capra_hircus_newGene_9422 (up, 0.0058, 0.3034), GRM2 (up, 0.0089, 0.2625), RBBP8 (up, 0.0116, 0.2518), C7H5orf45 (up, 0.0301, 0.2384), RRH (up, 0.0277, 0.2351), TSR3 (up, 0.0188, 0.2326), C1D (up, 0.0062, 0.1999), CC2D1B (up, 0.0303, 0.1309), KCNA5 (down, 0.0262, -0.1884), SP2 (down, 0.0328, -0.1892), THSD7A (down, 0.0294, -0.2382), ASMTL (down, 0.0037, -0.2855), ENPEP (down, 0.0054, -0.2921), TIE1 (down, 0.0065, -0.3025)
MSTRG.46907.1	down	0.0377	-1.357	Capra_hircus_newGene_12502 (up, 0.0035, 0.2992), SIPA1L2 (down, 0.0443, -0.1743), CAV1 (down, 0.0218, -0.1919), CLCC1 (down, 0.0063, -0.2214), FOLR2 (down, 0.0427, -0.2274), FHL5 (down, 0.032, -0.2352), LOC102189713 (down, 0.0178, -0.2676), MXD4 (down, 0.0019, -0.2706), ANGPTL1 (down, 0.0101, -0.2857), PRKCDBP (down, 0.0011, -0.3262)
MSTRG.47028.7	down	0.0126	-3.422	LOC102177708 (down, 0.0034, -0.3300), MRPS18B (up, 0.0189, 0.2197), FAM169B (down, 0.047, -0.2005)
MSTRG.47199.1	down	0.0119	-3.337	LOC102177708 (down, 0.0034, -0.3300), Capra_hircus_newGene_10117 (up, 0.0368, 0.2150), RFX2 (down, 0.0003, -0.4097)

DE ncRNA	Regulated	P value	log2Fold Change	Target mRNA (Regulated, P value, log2Fold Change)
MSTRG.47214.1	down	0.0104	-2.536	LOC102177708 (down, 0.0034, -0.3300), FAM169B (down, 0.047, -0.2005)
MSTRG.47363.1	down	0.0193	-1.657	AKR1B1 (down, 0.0117, -0.2830), CMYA5 (up, 0.0425, 0.1700), RASA1 (up, 0.0378, 0.1485), PMM1 (down, 0.0377, -0.2179), CD248 (down, 0.0042, -0.3239)
MSTRG.47464.1	up	0.0000	8.539	CXCR2 (up, 0.0253, 0.2180), LOC108635404 (up, 0.0002, 0.3433), UROS (up, 0.0032, 0.3268), LOC108635390 (up, 0.0014, 0.3178), DDX52 (up, 0.0099, 0.2227), LOC102172005 (up, 0.0281, 0.0851), STMN3 (down, 0.0379, -0.1576), CERCAM (down, 0.0179, -0.2276)
MSTRG.47687.1	down	0.0490	-1.223	Capra_hircus_newGene_18403 (down, 0.0486, -0.2210), POU2F1 (up, 0.0325, 0.1270), LOC102183952 (up, 0.0381, 0.2228)
MSTRG.47788.3	up	0.0034	3.893	TNFRSF18 (up, 0.0027, 0.3213), Capra_hircus_newGene_9422 (up, 0.0058, 0.3034), MAT1A (up, 0.0042, 0.2817), TSTD3 (up, 0.0406, 0.2313), LOC106503969 (up, 0.0224, 0.2105), HS6ST3 (up, 0.0216, 0.2028), ASMTL (down, 0.0037, -0.2855)
MSTRG.47808.9	up	0.0180	3.363	KRT7 (up, 0.0017, 0.3227), GLI1 (up, 0.0045, 0.2678)
MSTRG.47935.2	down	0.0328	-2.757	LOC102183952 (up, 0.0381, 0.2228)
MSTRG.48011.1	down	0.0251	-1.579	TRIM9 (down, 0.0324, -0.2330), COPE (down, 0.0237, -0.2160), SSR2 (down, 0.035, -0.1160), Capra_hircus_newGene_2167 (up, 0.0015, 0.3595), Capra_hircus_newGene_10897 (up, 0.044, 0.2049), KLHL21 (down, 0.0117, -0.1713), FBXL22 (down, 0.0168, -0.2050), RIMS3 (down, 0.0428, -0.2172), FOLR2 (down, 0.0427, -0.2274), ACTA2 (down, 0.0133, -0.2594), Capra_hircus_newGene_1595 (down, 0.0044, -0.2963), MRGPRF (down, 0.0001, -0.4224)
MSTRG.48015.1	up	0.0122	1.648	KY (up, 0.0109, 0.2881), GRM2 (up, 0.0089, 0.2625), APLN (up, 0.0178, 0.2470), CCDC66 (up, 0.0438, 0.2116), RPE (up, 0.0204, 0.1995), AMZ2 (up, 0.0447, 0.1973), TRMT10C (up, 0.0383, 0.1858), IDS (down, 0.0246, -0.1402), SP2 (down, 0.0328, -0.1892), AIP (down, 0.0216, -0.1957), SLC5A8 (down, 0.0181, -0.2358), ASMTL (down, 0.0037, -0.2855)
MSTRG.48174.1	up	0.0206	2.220	WRN (up, 0.0188, 0.1520), ATP13A4 (up, 0.0000, 0.5377), CD200 (up, 0.0069, 0.2948), KY (up, 0.0109, 0.2881), GLMN (up, 0.0137, 0.2399), LOC102186466 (up, 0.0416, 0.2137), RPE (up, 0.0204, 0.1995), TMEM123 (up, 0.0093, 0.1929), DCK (up, 0.034, 0.1895), TRMT10C (up, 0.0383, 0.1858), CASP3 (up, 0.0416, 0.1591), AIP (down, 0.0216, -0.1957), SLC25A19 (down, 0.049, -0.1966), THBS2 (down, 0.008, -0.2837), SOX4 (down, 0.0000, -1.1673)
MSTRG.4827.1	up	0.0463	1.393	GLMN (up, 0.0137, 0.2399), PLAU (up, 0.0319, 0.2019), TRMT13 (up, 0.0348, 0.1971), LOC102173760 (up, 0.0397, 0.1705), BCLAF1 (up, 0.0065, 0.1472), DNASE1L2 (down, 0.0235, -0.2394)

DE ncRNA	Regulated	P value	log2Fold Change	Target mRNA (Regulated, P value, log2Fold Change)
MSTRG.48456.9	up	0.0138	2.772	FAM111B (up, 0.0007, 0.3640), CD40 (up, 0.0437, 0.2030), MMP12 (up, 0.0016, 0.2779), PTGS2 (up, 0.0081, 0.2594), SPTA1 (up, 0.0153, 0.2057), CHST2 (up, 0.0049, 0.1866), HS3ST1 (up, 0.0332, 0.1818)
MSTRG.4872.1	up	0.0079	2.359	MARCH7 (up, 0.0025, 0.1770), TMEM221 (up, 0.0000, 0.3318), Capra_hircus_newGene_4795 (up, 0.0034, 0.2777), GLI1 (up, 0.0045, 0.2678), ITGA2 (up, 0.0262, 0.2254), C1D (up, 0.0062, 0.1999), RPE (up, 0.0204, 0.1995), MLX (up, 0.0488, 0.1363)
MSTRG.4896.1	down	0.0439	-1.352	TARS2 (up, 0.0062, 0.2366)
MSTRG.49210.1	down	0.0342	-1.997	GLI1 (up, 0.0045, 0.2678), PIK3CB (up, 0.0082, 0.1974), PTCH2 (up, 0.0088, 0.1971), SHTN1 (up, 0.0417, 0.1655), RFX2 (down, 0.0003, -0.4097)
MSTRG.49274.2	down	0.0183	-3.006	ADIPOR2 (down, 0.0441, -0.1419), SLC9A3R2 (down, 0.0483, -0.1615), SIPA1L2 (down, 0.0443, -0.1743), FBXL22 (down, 0.0168, -0.2050), RAB3IL1 (down, 0.0492, -0.2194), DACT1 (down, 0.0422, -0.2224), FOLR2 (down, 0.0427, -0.2274), C1QC (down, 0.041, -0.2304), MXD4 (down, 0.0019, -0.2706), ANGPTL1 (down, 0.0101, -0.2857), PRKCDBP (down, 0.0011, -0.3262)
MSTRG.49414.1	up	0.0008	3.414	CXCR2 (up, 0.0253, 0.2180), LOC108635404 (up, 0.0002, 0.3433), LOC108633201 (up, 0.0009, 0.3354), UROS (up, 0.0032, 0.3268), LOC108635390 (up, 0.0014, 0.3178), Capra_hircus_newGene_1504 (up, 0.0118, 0.2767), DDX52 (up, 0.0099, 0.2227), LOC102172005 (up, 0.0281, 0.0851), STMN3 (down, 0.0379, -0.1576), CERCAM (down, 0.0179, -0.2276)
MSTRG.49464.7	down	0.0057	-2.596	BIRC5 (up, 0.0088, 0.2550), RALGPS2 (up, 0.0216, 0.1380), DPP6 (up, 0.033, 0.2408), USP3 (up, 0.042, 0.1117), LDAH (up, 0.0313, 0.1579)
MSTRG.4960.6	up	0.0189	2.107	RALGPS2 (up, 0.0216, 0.1380), ZNF260 (up, 0.0000, 0.4445), TARM1 (up, 0.0096, 0.2777), C7H5orf45 (up, 0.0301, 0.2384), TARS2 (up, 0.0062, 0.2366), UBOX5 (up, 0.0322, 0.2277), PUS7 (up, 0.013, 0.2169), CDC40 (up, 0.0031, 0.2084), LOC102169288 (up, 0.03, 0.2069), FSD1L (up, 0.0364, 0.2032), NUP54 (up, 0.0119, 0.1988), METTL17 (up, 0.0256, 0.1940), CEP89 (down, 0.0356, -0.2204), THSD7A (down, 0.0294, -0.2382), SOX4 (down, 0.0000, -1.1673)
MSTRG.50040.23	up	0.0063	5.237	LOC108634682 (up, 0.0499, 0.1580)
MSTRG.50083.1	up	0.0320	1.548	PNPT1 (up, 0.0173, 0.1880), TARM1 (up, 0.0096, 0.2777), RBBP8 (up, 0.0116, 0.2518), Capra_hircus_newGene_17456 (up, 0.0337, 0.2401), TBX1 (up, 0.0236, 0.2136), LOC102169288 (up, 0.03, 0.2069), CXADR (up, 0.009, 0.1649), CC2D1B (up, 0.0303, 0.1309), KCNA5 (down, 0.0262, -0.1884), SP2 (down, 0.0328, -0.1892), ARFIP1 (down, 0.0054, -0.2133), VIM (down, 0.0049, -0.2610), MXD4 (down, 0.0019, -0.2706), ENPEP

DE ncRNA	Regulated	P value	log2Fold Change	Target mRNA (Regulated, P value, log2Fold Change)
				(down, 0.0054, -0.2921), TIE1 (down, 0.0065, -0.3025), SPON2 (down, 0.0011, -0.3677), SOX4 (down, 0.0000, -1.1673)
MSTRG.50083.2	down	0.0155	-3.080	Capra_hircus_newGene_12502 (up, 0.0035, 0.2992), DDX47 (up, 0.0314, 0.1675), ENGASE (down, 0.0116, -0.2122), CLCC1 (down, 0.0063, -0.2214)
MSTRG.50312.3	down	0.0280	-2.971	TRAPPC13 (up, 0.0458, 0.1645)
MSTRG.50352.1	down	0.0227	-1.373	COG1 (down, 0.0003, -0.3120), LOC106503979 (down, 0.0184, -0.2280), DPP6 (up, 0.033, 0.2408), ABCE1 (up, 0.0424, 0.1445)
MSTRG.50449.5	down	0.0137	-2.422	LOC102177708 (down, 0.0034, -0.3300)
MSTRG.50489.4	down	0.0402	-2.252	VWA2 (down, 0.0389, -0.2320), CNR1 (down, 0.0117, -0.2250), SLC35G1 (up, 0.0253, 0.2343), LOC102179192 (up, 0.0383, 0.2335), C25H16orf59 (up, 0.0398, 0.2287), E2F3 (up, 0.0218, 0.1952), LOC102168757 (down, 0.0366, -0.2224), TRPM6 (down, 0.044, -0.2277), PRUNE2 (down, 0.025, -0.2336)
MSTRG.50548.9	up	0.0000	8.612	PNPT1 (up, 0.0173, 0.1880), LOC108635404 (up, 0.0002, 0.3433), LOC108633201 (up, 0.0009, 0.3354), TMEM221 (up, 0.0000, 0.3318), Capra_hircus_newGene_1504 (up, 0.0118, 0.2767), LOC102176870 (up, 0.0088, 0.2564), RRH (up, 0.0277, 0.2351), C1D (up, 0.0062, 0.1999), STMN3 (down, 0.0379, -0.1576), DYRK1B (down, 0.0187, -0.1879), SP2 (down, 0.0328, -0.1892), CERCAM (down, 0.0179, -0.2276), LSAMP (down, 0.0286, -0.2352)
MSTRG.50864.30	up	0.0476	2.444	CIDEA (down, 0.0015, -0.3405)
MSTRG.51287.1	down	0.0199	-1.329	SLC39A3 (down, 0.0162, -0.2300), SOX18 (down, 0.0463, -0.2230), RNF217 (down, 0.0289, -0.1620), CABYR (up, 0.0000, 0.5116), Capra_hircus_newGene_5769 (up, 0.0088, 0.2831), Capra_hircus_newGene_1952 (up, 0.0029, 0.2627), Capra_hircus_newGene_17456 (up, 0.0337, 0.2401), WDTC1 (down, 0.0393, -0.1113), AMBRA1 (down, 0.0198, -0.1326), PNPLA6 (down, 0.0213, -0.1405), TSC1 (down, 0.0098, -0.1441), PPP1R7 (down, 0.0455, -0.1478), CTU1 (down, 0.0105, -0.1511), RUBCN (down, 0.0015, -0.1962), APBA1 (down, 0.0249, -0.2197), MARCH2 (down, 0.0155, -0.2434), ZBTB47 (down, 0.0011, -0.2435), C28H10orf10 (down, 0.0283, -0.2478), PLAC9 (down, 0.0092, -0.2905), PRKD1 (down, 0.0013, -0.3107), SEPTIN6 (down, 0.0016, -0.3371), GAS1 (down, 0.0002, -0.3759)
MSTRG.51442.3	up	0.0448	2.113	Capra_hircus_newGene_12426 (down, 0.0271, -0.2190), NELL2 (up, 0.0426, 0.2278), MRPS18B (up, 0.0189, 0.2197)
MSTRG.51473.1	up	0.0304	2.335	PRSS12 (up, 0.0286, 0.2448), PXYLP1 (up, 0.034, 0.2322), PRRG4 (up, 0.0178, 0.2181)

DE ncRNA	Regu lated	P value	log2Fold Change	Target mRNA (Regulated, P value, log2Fold Change)
MSTRG.51 615.1	down	0.0320	-2.300	FUCA1 (down, 0.0231, -0.2070), CANT1 (down, 0.0341, -0.1450), SSR2 (down, 0.035, -0.1160), Capra_hircus_newGene_2167 (up, 0.0015, 0.3595), OS9 (down, 0.037, -0.1297), ADIPOR2 (down, 0.0441, -0.1419), TJP2 (down, 0.0315, -0.1445), KLHL21 (down, 0.0117, -0.1713), BICDL2 (down, 0.0357, -0.2026), RIMS3 (down, 0.0428, -0.2172), SEPN1 (down, 0.0023, -0.2478), GPR153 (down, 0.0185, -0.2531), TSPAN33 (down, 0.0138, -0.2583), TMEM119 (down, 0.0129, -0.2805)
MSTRG.51 681.1	down	0.0467	-1.853	BIRC5 (up, 0.0088, 0.2550), RALGPS2 (up, 0.0216, 0.1380), BZW2 (up, 0.017, 0.2133), LDAH (up, 0.0313, 0.1579), RYR2 (down, 0.0101, -0.2808), RHOB (down, 0.0039, -0.3015)
MSTRG.51 687.4	down	0.0022	-4.782	PAPD4 (up, 0.0085, 0.1790), PIK3CB (up, 0.0082, 0.1974), SLC31A1 (up, 0.0102, 0.1874), POC5 (up, 0.0476, 0.1785), TRAPPC13 (up, 0.0458, 0.1645), PTMS (down, 0.0402, -0.1617), PMM1 (down, 0.0377, -0.2179)
MSTRG.51 751.1	up	0.0307	1.431	KRT7 (up, 0.0017, 0.3227), GLI1 (up, 0.0045, 0.2678), DTD2 (up, 0.0089, 0.2513), SHTN1 (up, 0.0417, 0.1655), AIP (down, 0.0216, -0.1957)
MSTRG.52 0.1	up	0.0465	1.456	FAM111B (up, 0.0007, 0.3640), CD40 (up, 0.0437, 0.2030), MMP12 (up, 0.0016, 0.2779), LOC102174470 (up, 0.0021, 0.2683), PTGS2 (up, 0.0081, 0.2594), SLC16A1 (up, 0.0401, 0.2321), KCNJ15 (up, 0.0286, 0.2298), SPTA1 (up, 0.0153, 0.2057), UAP1 (up, 0.0394, 0.2023), HS3ST1 (up, 0.0332, 0.1818)
MSTRG.52 057.3	down	0.0393	-1.698	PAPD4 (up, 0.0085, 0.1790), LOC108633201 (up, 0.0009, 0.3354), DIO2 (up, 0.0195, 0.2441), TNFSF4 (up, 0.0257, 0.2385), NMD3 (up, 0.0038, 0.1877), SLC31A1 (up, 0.0102, 0.1874), BLOC1S6 (up, 0.0432, 0.1478), LOC102174081 (down, 0.0163, -0.2513)
MSTRG.52 628.9	up	0.0050	3.705	LOC108634682 (up, 0.0499, 0.1580), MAT1A (up, 0.0042, 0.2817), Capra_hircus_newGene_12726 (up, 0.0159, 0.2591), TAF1D (up, 0.0177, 0.2439), LOC106503943 (up, 0.0485, 0.1846)
MSTRG.52 657.1	down	0.0373	-2.568	ADAM22 (down, 0.0158, -0.2070), EHD2 (down, 0.0092, -0.2020), CCDC22 (down, 0.0389, -0.1870), TSC1 (down, 0.0098, -0.1441), CAV1 (down, 0.0218, -0.1919), PTRF (down, 0.0451, -0.1989), SDPR (down, 0.0411, -0.2303), LOC102189713 (down, 0.0178, -0.2676), CDH13 (down, 0.0049, -0.2895)
MSTRG.52 999.1	down	0.0373	-1.872	Capra_hircus_newGene_14774 (down, 0.037, -0.2305), FHL5 (down, 0.032, -0.2352), RYR2 (down, 0.0101, -0.2808)
MSTRG.53 02.2	up	0.0240	2.691	MARCH7 (up, 0.0025, 0.1770), TMEM221 (up, 0.0000, 0.3318), Capra_hircus_newGene_4795 (up, 0.0034, 0.2777), PXYLP1 (up, 0.034, 0.2322), Capra_hircus_newGene_11971 (up, 0.037, 0.2193), MLX (up, 0.0488, 0.1363), SCPEP1 (down, 0.0054, -0.2938)
MSTRG.53 210.1	up	0.0000	3.259	LOC102168573 (up, 0.0023, 0.2800), Capra_hircus_newGene_18403 (down, 0.0486, -0.2210), FMO4 (up, 0.006, 0.3088), LOC102174170 (up,

DE ncRNA	Regulated	P value	log2Fold Change	Target mRNA (Regulated, P value, log2Fold Change)
				0.0065, 0.3036), DTD2 (up, 0.0089, 0.2513), MTHFD1 (up, 0.0114, 0.1881), ZBTB6 (down, 0.0223, -0.1686), RUBCN (down, 0.0015, -0.1962)
MSTRG.53348.1	up	0.0461	1.646	FAM111B (up, 0.0007, 0.3640), CXCR2 (up, 0.0253, 0.2180), AMD1 (up, 0.0459, 0.1660), PTGS2 (up, 0.0081, 0.2594), KCNJ15 (up, 0.0286, 0.2298), SPTA1 (up, 0.0153, 0.2057), KDM4C (up, 0.0411, 0.1565), LOC102172005 (up, 0.0281, 0.0851)
MSTRG.53354.9	down	0.0311	-1.985	LOC102176218 (down, 0.0131, -0.2770), Capra_hircus_newGene_18403 (down, 0.0486, -0.2210), LOC100861174 (down, 0.0467, -0.2150), DIO2 (up, 0.0195, 0.2441), METTL17 (up, 0.0256, 0.1940), LOC108636556 (down, 0.0465, -0.2001), LOC102174081 (down, 0.0163, -0.2513)
MSTRG.53356.2	down	0.0006	-4.189	NUCB1 (down, 0.0184, -0.2330), Capra_hircus_newGene_15790 (up, 0.0496, 0.2215), ADIPOR2 (down, 0.0441, -0.1419), PPM1A (down, 0.0219, -0.1521), SLC9A3R2 (down, 0.0483, -0.1615), KLHL21 (down, 0.0117, -0.1713), PTRF (down, 0.0451, -0.1989), GFAP (down, 0.0251, -0.2006), BICDL2 (down, 0.0357, -0.2026), RIMS3 (down, 0.0428, -0.2172), RAB3IL1 (down, 0.0492, -0.2194), IGFBP4 (down, 0.0412, -0.2262), FBLN5 (down, 0.0366, -0.2349), LHX6 (down, 0.0316, -0.2381), HIC1 (down, 0.0303, -0.2448), MEX3B (down, 0.0251, -0.2489), SERTM1 (down, 0.0056, -0.3107)
MSTRG.53356.4	down	0.0155	-3.819	LOC102176218 (down, 0.0131, -0.2770), Capra_hircus_newGene_18403 (down, 0.0486, -0.2210), DIO2 (up, 0.0195, 0.2441), MGME1 (up, 0.0218, 0.2416), METTL17 (up, 0.0256, 0.1940), UTP6 (up, 0.0467, 0.1493), LOC108636556 (down, 0.0465, -0.2001), LOC102174081 (down, 0.0163, -0.2513)
MSTRG.53357.5	down	0.0158	-2.606	HSPA2 (down, 0.033, -0.2230), LOC100861174 (down, 0.0467, -0.2150), NME7 (up, 0.0047, 0.2694), C7H5orf45 (up, 0.0301, 0.2384), UBOX5 (up, 0.0322, 0.2277), PUS7 (up, 0.013, 0.2169), METTL17 (up, 0.0256, 0.1940), SRSF5 (up, 0.0445, 0.1865), MCC (up, 0.0197, 0.1551), LOC108636556 (down, 0.0465, -0.2001), LOC102174081 (down, 0.0163, -0.2513), LOC102187755 (down, 0.0004, -0.3721)
MSTRG.53499.10	up	0.0105	4.780	LOC108634682 (up, 0.0499, 0.1580), MAT1A (up, 0.0042, 0.2817), Capra_hircus_newGene_12726 (up, 0.0159, 0.2591), TAF1D (up, 0.0177, 0.2439), LOC106503943 (up, 0.0485, 0.1846)
MSTRG.53543.1	down	0.0041	-4.648	LOC102177708 (down, 0.0034, -0.3300), Capra_hircus_newGene_18572 (up, 0.0001, 0.4124), FZD8 (up, 0.0352, 0.1967), MEF2D (down, 0.0383, -0.1429), Capra_hircus_newGene_8638 (down, 0.0074, -0.1663), LOC102185708 (down, 0.0452, -0.1771), GPR153 (down, 0.0185, -0.2531), DDAH2 (down, 0.0099, -0.2543)
MSTRG.53628.3	up	0.0045	2.434	LOC108635404 (up, 0.0002, 0.3433), LOC108635390 (up, 0.0014, 0.3178), DDX52 (up, 0.0099, 0.2227), EPPK1 (up, 0.012, 0.2178), CERCAM

DE ncRNA	Regulated	P value	log2Fold Change	Target mRNA (Regulated, P value, log2Fold Change)
				(down, 0.0179, -0.2276), LSAMP (down, 0.0286, -0.2352), LOC108638594 (down, 0.0155, -0.2654)
MSTRG.53655.1	down	0.0296	-1.638	MARCKSL1 (up, 0.0218, 0.1985), PITX2 (down, 0.0453, -0.1976), FLNC (down, 0.0403, -0.2226), RFX2 (down, 0.0003, -0.4097)
MSTRG.538.2	up	0.0027	1.922	CXCR2 (up, 0.0253, 0.2180), WRN (up, 0.0188, 0.1520), ATP13A4 (up, 0.0000, 0.5377), LOC108635404 (up, 0.0002, 0.3433), UROS (up, 0.0032, 0.3268), LOC108635390 (up, 0.0014, 0.3178), KCND2 (up, 0.0141, 0.2751), DDX52 (up, 0.0099, 0.2227), DCK (up, 0.034, 0.1895), CCDC50 (up, 0.0212, 0.1801), SRSF3 (up, 0.0396, 0.1062), LOC102172005 (up, 0.0281, 0.0851), STMN3 (down, 0.0379, -0.1576), SIDT2 (down, 0.0011, -0.2037), CERCAM (down, 0.0179, -0.2276), LSAMP (down, 0.0286, -0.2352)
MSTRG.53873.4	up	0.0313	1.223	CXCR2 (up, 0.0253, 0.2180), ZNRF2 (up, 0.0382, 0.1620), WRN (up, 0.0188, 0.1520), Capra_hircus_newGene_18572 (up, 0.0001, 0.4124), Capra_hircus_newGene_21230 (up, 0.0007, 0.3532), LOC108635390 (up, 0.0014, 0.3178), SCN8A (up, 0.0164, 0.2530), RRH (up, 0.0277, 0.2351), TSR3 (up, 0.0188, 0.2326), DDX52 (up, 0.0099, 0.2227), Capra_hircus_newGene_10897 (up, 0.044, 0.2049), DCK (up, 0.034, 0.1895), NT5C2 (up, 0.0483, 0.1682), CXADR (up, 0.009, 0.1649), SRSF3 (up, 0.0396, 0.1062), STMN3 (down, 0.0379, -0.1576), FBXL22 (down, 0.0168, -0.2050), ENPP4 (down, 0.0217, -0.2271), CERCAM (down, 0.0179, -0.2276), TMEM35A (down, 0.0306, -0.2444), SNX24 (down, 0.0081, -0.2668), TIE1 (down, 0.0065, -0.3025), DZIP1L (down, 0.0009, -0.3689), SOX4 (down, 0.0000, -1.1673)
MSTRG.53880.3	up	0.0098	4.520	MMP13 (up, 0.048, 0.1680), LOC108635404 (up, 0.0002, 0.3433), CERCAM (down, 0.0179, -0.2276), ADAMTS15 (down, 0.0057, -0.3114)
MSTRG.53893.2	down	0.0463	-2.205	SOX18 (down, 0.0463, -0.2230), EHD2 (down, 0.0092, -0.2020), CCDC22 (down, 0.0389, -0.1870), CAPNS1 (down, 0.0452, -0.1094), BCAP31 (down, 0.0218, -0.1273), PNPLA6 (down, 0.0213, -0.1405)
MSTRG.54087.1	down	0.0017	-3.094	AKR1B1 (down, 0.0117, -0.2830), HSPA2 (down, 0.033, -0.2230), LOC100861174 (down, 0.0467, -0.2150), LOC108634363 (down, 0.0361, -0.1015)
MSTRG.54483.2	up	0.0049	3.774	STEAP2 (up, 0.0332, 0.2200), LOC102168852 (up, 0.0003, 0.3332), STAT4 (up, 0.0143, 0.2454), UAP1 (up, 0.0394, 0.2023), CHST2 (up, 0.0049, 0.1866)
MSTRG.54591.4	up	0.0127	2.583	FAM111B (up, 0.0007, 0.3640), STEAP2 (up, 0.0332, 0.2200), CD40 (up, 0.0437, 0.2030), Capra_hircus_newGene_22856 (up, 0.0029, 0.3335), MMP12 (up, 0.0016, 0.2779), LOC102174470 (up, 0.0021, 0.2683), PTGS2 (up, 0.0081, 0.2594), KCNJ15 (up, 0.0286, 0.2298), SPTA1 (up, 0.0153, 0.2057), UAP1 (up, 0.0394, 0.2023), CHST2 (up, 0.0049, 0.1866)

DE ncRNA	Regulated	P value	log2Fold Change	Target mRNA (Regulated, P value, log2Fold Change)
MSTRG.54655.1	down	0.0093	-4.181	AKR1B1 (down, 0.0117, -0.2830), RASA1 (up, 0.0378, 0.1485), TMEM119 (down, 0.0129, -0.2805), CD248 (down, 0.0042, -0.3239), DZIP1L (down, 0.0009, -0.3689)
MSTRG.54720.11	up	0.0122	3.243	MMP13 (up, 0.048, 0.1680), Capra_hircus_newGene_4795 (up, 0.0034, 0.2777), ITGA2 (up, 0.0262, 0.2254), MLX (up, 0.0488, 0.1363), LOC108637962 (up, 0.0491, 0.1324)
MSTRG.54804.1	down	0.0028	-1.816	TLR1 (up, 0.007, 0.2740), WRN (up, 0.0188, 0.1520), ATP13A4 (up, 0.0000, 0.5377), KLHL29 (up, 0.0013, 0.2747), ZNF165 (up, 0.0284, 0.2438), LOC102182782 (up, 0.0312, 0.2428), C1D (up, 0.0062, 0.1999), CCDC50 (up, 0.0212, 0.1801), PITX2 (down, 0.0453, -0.1976), LOC106503362 (down, 0.0208, -0.2331), RFX2 (down, 0.0003, -0.4097)
MSTRG.54862.1	up	0.0125	1.586	LOC102168573 (up, 0.0023, 0.2800), KRT7 (up, 0.0017, 0.3227), TARM1 (up, 0.0096, 0.2777), DTD2 (up, 0.0089, 0.2513), ZNF165 (up, 0.0284, 0.2438), LOC102169288 (up, 0.03, 0.2069), SP2 (down, 0.0328, -0.1892), AIP (down, 0.0216, -0.1957), RUBCN (down, 0.0015, -0.1962), PITX2 (down, 0.0453, -0.1976)
MSTRG.54930.5	down	0.0038	-2.007	LOC102177708 (down, 0.0034, -0.3300), ZNF165 (up, 0.0284, 0.2438), NET1 (down, 0.0475, -0.1192), ATP8B2 (down, 0.0088, -0.1796), PITX2 (down, 0.0453, -0.1976), RP2 (down, 0.0474, -0.2078)
MSTRG.55185.1	down	0.0052	-1.821	CNR1 (down, 0.0117, -0.2250), E2F3 (up, 0.0218, 0.1952), TXNDC5 (up, 0.0176, 0.1726), RASSF7 (down, 0.0413, -0.2277), YPEL1 (down, 0.0203, -0.2455)
MSTRG.55260.3	down	0.0098	-1.874	LOC102182782 (up, 0.0312, 0.2428), LOC102183952 (up, 0.0381, 0.2228), CCDC50 (up, 0.0212, 0.1801), Capra_hircus_newGene_21088 (down, 0.0183, -0.1713), SNRPN (down, 0.016, -0.1981), SIDT2 (down, 0.0011, -0.2037), LOC102182149 (down, 0.0116, -0.2094), LOC106503362 (down, 0.0208, -0.2331), TPPP3 (down, 0.0084, -0.2747)
MSTRG.55695.3	down	0.0017	-3.287	TPPP3 (down, 0.0084, -0.2747), Capra_hircus_newGene_19483 (down, 0.0094, -0.2813)
MSTRG.55778.2	up	0.0056	2.724	Capra_hircus_newGene_9735 (up, 0.0153, 0.2735), LOC102176870 (up, 0.0088, 0.2564), MRPS18B (up, 0.0189, 0.2197), TBX1 (up, 0.0236, 0.2136), LOC102169288 (up, 0.03, 0.2069), MTHFD1 (up, 0.0114, 0.1881), MRPS27 (up, 0.0254, 0.1649), CIDEA (down, 0.0015, -0.3405)
MSTRG.55802.1	down	0.0380	-1.523	KRT36 (down, 0.0001, -0.4450), FUCA1 (down, 0.0231, -0.2070), CANT1 (down, 0.0341, -0.1450), Capra_hircus_newGene_5074 (up, 0.0067, 0.3016), NCSTN (down, 0.0307, -0.1114), OS9 (down, 0.037, -0.1297), AMBRA1 (down, 0.0198, -0.1326), TSC1 (down, 0.0098, -0.1441), TJP2 (down, 0.0315, -0.1445), PPP1R7 (down, 0.0455, -0.1478), PPM1A (down, 0.0219, -0.1521), SNX33 (down, 0.0234, -0.1527), TOX2 (down, 0.0444, -0.1819), TEF (down, 0.0243, -0.1869), DYRK1B (down, 0.0187, -0.1879), KLHDC8B (down, 0.0443, -0.2272), ZBTB47 (down, 0.0011, -0.2435),

DE ncRNA	Regulated	P value	log2Fold Change	Target mRNA (Regulated, P value, log2Fold Change)
				TMEM35A (down, 0.0306, -0.2444), SEPN1 (down, 0.0023, -0.2478), TGFB1I1 (down, 0.0171, -0.2526), TSPAN33 (down, 0.0138, -0.2583), SPTB (down, 0.0032, -0.2967), SEPTIN6 (down, 0.0016, -0.3371), GAS1 (down, 0.0002, -0.3759), MRGPRF (down, 0.0001, -0.4224)
MSTRG.55880.4	down	0.0082	-3.325	LOC102177708 (down, 0.0034, -0.3300), FZD8 (up, 0.0352, 0.1967), MEF2D (down, 0.0383, -0.1429), Capra_hircus_newGene_8638 (down, 0.0074, -0.1663), LOC102185708 (down, 0.0452, -0.1771)
MSTRG.55972.3	down	0.0052	-3.297	LOC102186466 (up, 0.0416, 0.2137), RPE (up, 0.0204, 0.1995), TMEM123 (up, 0.0093, 0.1929), TRAPPC13 (up, 0.0458, 0.1645), Capra_hircus_newGene_8638 (down, 0.0074, -0.1663), CD248 (down, 0.0042, -0.3239)
MSTRG.5602.7	down	0.0394	-2.863	LOC102177708 (down, 0.0034, -0.3300), FAM169B (down, 0.047, -0.2005)
MSTRG.56135.1	down	0.0275	-1.731	AKR1B1 (down, 0.0117, -0.2830), WRN (up, 0.0188, 0.1520), PPAT (up, 0.0255, 0.2475), GLMN (up, 0.0137, 0.2399), PLAU (up, 0.0319, 0.2019), CDCA7L (up, 0.015, 0.1647), OTOGL (down, 0.0094, -0.2432)
MSTRG.56282.2	down	0.0057	-2.301	TRAPPC13 (up, 0.0458, 0.1645), CD248 (down, 0.0042, -0.3239)
MSTRG.56283.2	up	0.0104	2.003	RNF217 (down, 0.0289, -0.1620), LOC102168852 (up, 0.0003, 0.3332), Capra_hircus_newGene_11982 (up, 0.0073, 0.3031), PVR (up, 0.0254, 0.2467), C8H9orf72 (up, 0.0236, 0.1854), TMEM87A (up, 0.0164, 0.1548), ZBTB6 (down, 0.0223, -0.1686), AKAP17A (down, 0.0305, -0.1924)
MSTRG.56329.1	up	0.0104	1.758	FAM111B (up, 0.0007, 0.3640), UROS (up, 0.0032, 0.3268), LOC102184252 (up, 0.0069, 0.2972), MMP12 (up, 0.0016, 0.2779), LOC102174470 (up, 0.0021, 0.2683), STAT4 (up, 0.0143, 0.2454), LOC102169889 (up, 0.0335, 0.2391), KCNJ15 (up, 0.0286, 0.2298), FASTKD2 (up, 0.0031, 0.2242), UAP1 (up, 0.0394, 0.2023), PREB (up, 0.0229, 0.1666), MATR3 (up, 0.0168, 0.1024)
MSTRG.56717.1	up	0.0050	3.966	MMP13 (up, 0.048, 0.1680), Capra_hircus_newGene_4795 (up, 0.0034, 0.2777), ITGA2 (up, 0.0262, 0.2254), LOC108637962 (up, 0.0491, 0.1324)
MSTRG.56721.1	down	0.0356	-1.729	LOC102177708 (down, 0.0034, -0.3300), LOC102185708 (down, 0.0452, -0.1771)
MSTRG.56724.17	up	0.0000	6.676	NELL2 (up, 0.0426, 0.2278), SLIT1 (up, 0.0482, 0.1882), Capra_hircus_newGene_14773 (up, 0.0472, 0.0905)
MSTRG.56724.6	up	0.0087	2.187	Capra_hircus_newGene_12726 (up, 0.0159, 0.2591), DTD2 (up, 0.0089, 0.2513)
MSTRG.56794.1	down	0.0323	-1.356	BIRC5 (up, 0.0088, 0.2550), RALGPS2 (up, 0.0216, 0.1380), Capra_hircus_newGene_9735 (up, 0.0153, 0.2735), NME7 (up, 0.0047, 0.2694), CDC40 (up, 0.0031, 0.2084), LOC102169288 (up, 0.03, 0.2069), MTHFD1 (up, 0.0114, 0.1881), MRPS27 (up, 0.0254, 0.1649), CIDEA (down, 0.0015, -0.3405)

DE ncRNA	Regulated	P value	log2Fold Change	Target mRNA (Regulated, P value, log2Fold Change)
MSTRG.56952.8	up	0.0244	2.471	Capra_hircus_newGene_21365 (up, 0.0024, 0.3424), LOC102172005 (up, 0.0281, 0.0851), SIDT2 (down, 0.0011, -0.2037), Capra_hircus_newGene_3630 (down, 0.0351, -0.2138), LOC108638604 (down, 0.0214, -0.2443)
MSTRG.57117.11	up	0.0437	3.778	FAM111B (up, 0.0007, 0.3640), STEAP2 (up, 0.0332, 0.2200), CD40 (up, 0.0437, 0.2030), MMP12 (up, 0.0016, 0.2779), PTGS2 (up, 0.0081, 0.2594), SPTA1 (up, 0.0153, 0.2057), CHST2 (up, 0.0049, 0.1866), HS3ST1 (up, 0.0332, 0.1818)
MSTRG.57202.1	down	0.0206	-2.115	CCDC22 (down, 0.0389, -0.1870), ZNRF2 (up, 0.0382, 0.1620), Capra_hircus_newGene_1952 (up, 0.0029, 0.2627), FGD6 (up, 0.0034, 0.2506), Capra_hircus_newGene_17456 (up, 0.0337, 0.2401), PNPLA6 (down, 0.0213, -0.1405), MRPL47 (down, 0.0424, -0.1918), CLCC1 (down, 0.0063, -0.2214), FHL5 (down, 0.032, -0.2352), LOC102189713 (down, 0.0178, -0.2676)
MSTRG.57229.2	down	0.0282	-1.821	POU2F1 (up, 0.0325, 0.1270), PPAT (up, 0.0255, 0.2475), UBOX5 (up, 0.0322, 0.2277), METTL17 (up, 0.0256, 0.1940), CDCA7L (up, 0.015, 0.1647), BCLAF1 (up, 0.0065, 0.1472), BCAP31 (down, 0.0218, -0.1273)
MSTRG.57630.1	up	0.0011	3.449	COG1 (down, 0.0003, -0.3120), RNF217 (down, 0.0289, -0.1620), Capra_hircus_newGene_1952 (up, 0.0029, 0.2627), FASTKD2 (up, 0.0031, 0.2242), CDC40 (up, 0.0031, 0.2084), C8H9orf72 (up, 0.0236, 0.1854), PPP1R7 (down, 0.0455, -0.1478), ZBTB6 (down, 0.0223, -0.1686), RUBCN (down, 0.0015, -0.1962), PRKD1 (down, 0.0013, -0.3107), SOX4 (down, 0.0000, -1.1673)
MSTRG.57646.2	up	0.0207	2.508	MMP13 (up, 0.048, 0.1680), Capra_hircus_newGene_4795 (up, 0.0034, 0.2777), ITGA2 (up, 0.0262, 0.2254), LOC108637962 (up, 0.0491, 0.1324)
MSTRG.57692.5	down	0.0392	-3.257	LOC108634363 (down, 0.0361, -0.1015)
MSTRG.5778.26	down	0.0093	-3.894	LOC102177708 (down, 0.0034, -0.3300), FAM169B (down, 0.047, -0.2005)
MSTRG.57876.1	up	0.0050	4.258	Capra_hircus_newGene_14773 (up, 0.0472, 0.0905),
MSTRG.58065.1	down	0.0215	-1.555	ZNF260 (up, 0.0000, 0.4445), TARM1 (up, 0.0096, 0.2777), Capra_hircus_newGene_1952 (up, 0.0029, 0.2627), FGD6 (up, 0.0034, 0.2506), Capra_hircus_newGene_17456 (up, 0.0337, 0.2401), TARS2 (up, 0.0062, 0.2366), FASTKD2 (up, 0.0031, 0.2242), CDC40 (up, 0.0031, 0.2084), NUP54 (up, 0.0119, 0.1988), CXADR (up, 0.009, 0.1649), CLCC1 (down, 0.0063, -0.2214), SOX4 (down, 0.0000, -1.1673)
MSTRG.58081.25	up	0.0000	6.243	RBM3 (up, 0.0002, 0.3306), Capra_hircus_newGene_11982 (up, 0.0073, 0.3031), MAT1A (up, 0.0042, 0.2817), TAF1D (up, 0.0177, 0.2439), PLEKHH2 (up, 0.0076, 0.2063), NUP54 (up, 0.0119, 0.1988), TRMT13

DE ncRNA	Regulated	P value	log2Fold Change	Target mRNA (Regulated, P value, log2Fold Change)
				(up, 0.0348, 0.1971), LOC106503943 (up, 0.0485, 0.1846), LOC102179921 (down, 0.0066, -0.2763)
MSTRG.58272.19	up	0.0216	2.178	FAM111B (up, 0.0007, 0.3640), CD40 (up, 0.0437, 0.2030), MMP12 (up, 0.0016, 0.2779), PTGS2 (up, 0.0081, 0.2594), SPTA1 (up, 0.0153, 0.2057), CHST2 (up, 0.0049, 0.1866), HS3ST1 (up, 0.0332, 0.1818)
MSTRG.58494.1	up	0.0016	3.529	FAM111B (up, 0.0007, 0.3640), STEAP2 (up, 0.0332, 0.2200), CD40 (up, 0.0437, 0.2030), LOC102174470 (up, 0.0021, 0.2683), PTGS2 (up, 0.0081, 0.2594), SPTA1 (up, 0.0153, 0.2057), UAP1 (up, 0.0394, 0.2023), CHST2 (up, 0.0049, 0.1866), HS3ST1 (up, 0.0332, 0.1818)
MSTRG.58570.1	down	0.0098	-2.398	LOC102185150 (down, 0.0323, -0.1983)
MSTRG.58695.9	up	0.0242	3.953	FAM111B (up, 0.0007, 0.3640), CD40 (up, 0.0437, 0.2030), MMP12 (up, 0.0016, 0.2779), PTGS2 (up, 0.0081, 0.2594), KCNJ15 (up, 0.0286, 0.2298), SPTA1 (up, 0.0153, 0.2057), CHST2 (up, 0.0049, 0.1866), HS3ST1 (up, 0.0332, 0.1818)
MSTRG.58766.1	up	0.0207	1.456	LOC102168573 (up, 0.0023, 0.2800), MYO3B (up, 0.0075, 0.3008), MRPS18B (up, 0.0189, 0.2197), MTHFD1 (up, 0.0114, 0.1881), C8H9orf72 (up, 0.0236, 0.1854), SNURF (up, 0.0418, 0.1668), MRPS27 (up, 0.0254, 0.1649), TMEM87A (up, 0.0164, 0.1548), ZBTB6 (down, 0.0223, -0.1686), AWAT1 (down, 0.0158, -0.2730), PRKD1 (down, 0.0013, -0.3107)
MSTRG.58831.9	up	0.0358	2.963	LOC108638522 (up, 0.0374, 0.1840), ZNRF2 (up, 0.0382, 0.1620), Capra_hircus_newGene_21230 (up, 0.0007, 0.3532), LOC108633201 (up, 0.0009, 0.3354), TNFRSF18 (up, 0.0027, 0.3213), Capra_hircus_newGene_1504 (up, 0.0118, 0.2767), SCN8A (up, 0.0164, 0.2530)
MSTRG.58891.1	down	0.0244	-1.490	LOC106503979 (down, 0.0184, -0.2280), CCDC112 (up, 0.0468, 0.2230), LOC102183952 (up, 0.0381, 0.2228), SLC33A1 (up, 0.0454, 0.1647), MATR3 (up, 0.0168, 0.1024)
MSTRG.5971.1	up	0.0182	2.537	TMEM221 (up, 0.0000, 0.3318), GLI1 (up, 0.0045, 0.2678), GRM2 (up, 0.0089, 0.2625), C1D (up, 0.0062, 0.1999), RPE (up, 0.0204, 0.1995), TRMT10C (up, 0.0383, 0.1858), SHTN1 (up, 0.0417, 0.1655), AIP (down, 0.0216, -0.1957), SLC25A19 (down, 0.049, -0.1966), GDA (down, 0.0318, -0.2011), SLC5A8 (down, 0.0181, -0.2358)
MSTRG.6005.8	up	0.0001	4.496	MMP13 (up, 0.048, 0.1680), TMEM221 (up, 0.0000, 0.3318), Capra_hircus_newGene_4795 (up, 0.0034, 0.2777), ITGA2 (up, 0.0262, 0.2254), EPPK1 (up, 0.012, 0.2178), MLX (up, 0.0488, 0.1363), LOC108637962 (up, 0.0491, 0.1324), LSAMP (down, 0.0286, -0.2352)
MSTRG.6169.1	up	0.0359	2.023	Capra_hircus_newGene_11982 (up, 0.0073, 0.3031), LOC102179921 (down, 0.0066, -0.2763)

DE ncRNA	Regu lated	P value	log2Fold Change	Target mRNA (Regulated, P value, log2Fold Change)
MSTRG.6171.7	down	0.0058	-2.509	MARCH7 (up, 0.0025, 0.1770), PPAT (up, 0.0255, 0.2475), DIO2 (up, 0.0195, 0.2441), TNFSF4 (up, 0.0257, 0.2385), C7H5orf45 (up, 0.0301, 0.2384), UBOX5 (up, 0.0322, 0.2277), C1D (up, 0.0062, 0.1999), METTL17 (up, 0.0256, 0.1940), NMD3 (up, 0.0038, 0.1877), CDCA7L (up, 0.015, 0.1647), MCC (up, 0.0197, 0.1551), BLOC1S6 (up, 0.0432, 0.1478), CEP89 (down, 0.0356, -0.2204), THSD7A (down, 0.0294, -0.2382), OTOGL (down, 0.0094, -0.2432), LOC102174081 (down, 0.0163, -0.2513)
MSTRG.6325.25	up	0.0067	2.938	WRN (up, 0.0188, 0.1520), LOC108635404 (up, 0.0002, 0.3433), UROS (up, 0.0032, 0.3268), LOC108635390 (up, 0.0014, 0.3178), KY (up, 0.0109, 0.2881), SCN8A (up, 0.0164, 0.2530), DDX52 (up, 0.0099, 0.2227), RPE (up, 0.0204, 0.1995), AMZ2 (up, 0.0447, 0.1973), TMEM123 (up, 0.0093, 0.1929), DCK (up, 0.034, 0.1895), STMN3 (down, 0.0379, -0.1576), DYRK1B (down, 0.0187, -0.1879), HSPA12B (down, 0.0426, -0.2022), ENPP4 (down, 0.0217, -0.2271)
MSTRG.6485.5	down	0.0400	-3.070	PAPD4 (up, 0.0085, 0.1790), PPAT (up, 0.0255, 0.2475), PIK3CB (up, 0.0082, 0.1974), SLC31A1 (up, 0.0102, 0.1874), PMM1 (down, 0.0377, -0.2179)
MSTRG.6485.7	up	0.0464	4.109	CROT (up, 0.0482, 0.2166), Capra_hircus_newGene_14773 (up, 0.0472, 0.0905), ALG2 (down, 0.034, -0.1464)
MSTRG.6557.1	down	0.0032	-2.812	SOX18 (down, 0.0463, -0.2230), ADAM22 (down, 0.0158, -0.2070), EHD2 (down, 0.0092, -0.2020), SSR2 (down, 0.035, -0.1160), CAPNS1 (down, 0.0452, -0.1094), NCSTN (down, 0.0307, -0.1114), BCAP31 (down, 0.0218, -0.1273), OS9 (down, 0.037, -0.1297), ADIPOR2 (down, 0.0441, -0.1419), SLC9A3R2 (down, 0.0483, -0.1615), KLHL21 (down, 0.0117, -0.1713), MRPL47 (down, 0.0424, -0.1918), PTRF (down, 0.0451, -0.1989), GFAP (down, 0.0251, -0.2006), BICDL2 (down, 0.0357, -0.2026), RIMS3 (down, 0.0428, -0.2172), RAB3IL1 (down, 0.0492, -0.2194), DACT1 (down, 0.0422, -0.2224), C1QC (down, 0.041, -0.2304), FBLN5 (down, 0.0366, -0.2349), LHX6 (down, 0.0316, -0.2381), HIC1 (down, 0.0303, -0.2448), SEPN1 (down, 0.0023, -0.2478), MXD4 (down, 0.0019, -0.2706), ANGPTL1 (down, 0.0101, -0.2857), STC2 (down, 0.0053, -0.3048), SERTM1 (down, 0.0056, -0.3107), SPON2 (down, 0.0011, -0.3677), MRGPRF (down, 0.0001, -0.4224)
MSTRG.6630.1	up	0.0467	1.609	Capra_hircus_newGene_1504 (up, 0.0118, 0.2767), UGGT2 (up, 0.027, 0.2456), KCNA1 (up, 0.0459, 0.2129), STMN3 (down, 0.0379, -0.1576), DYRK1B (down, 0.0187, -0.1879)
MSTRG.6843.1	down	0.0447	-1.623	Capra_hircus_newGene_15790 (up, 0.0496, 0.2215), LYPD5 (down, 0.0426, -0.1948), UBC (down, 0.0496, -0.1962), IGFBP4 (down, 0.0412, -0.2262)

DE ncRNA	Regulated	P value	log2Fold Change	Target mRNA (Regulated, P value, log2Fold Change)
MSTRG.6909.1	up	0.0163	1.644	TLR1 (up, 0.007, 0.2740), ZNF260 (up, 0.0000, 0.4445), RAB37 (up, 0.013, 0.2690), MGME1 (up, 0.0218, 0.2416), CDC40 (up, 0.0031, 0.2084), PLEKHH2 (up, 0.0076, 0.2063), FSD1L (up, 0.0364, 0.2032), NUP54 (up, 0.0119, 0.1988), TRMT13 (up, 0.0348, 0.1971), CDCA7L (up, 0.015, 0.1647), DHX36 (up, 0.0096, 0.1475), BCLAF1 (up, 0.0065, 0.1472), MATR3 (up, 0.0168, 0.1024), CEP89 (down, 0.0356, -0.2204)
MSTRG.7125.2	down	0.0375	-2.187	SPAG1 (up, 0.003, 0.2879), LOC102182149 (down, 0.0116, -0.2094), LOC108638604 (down, 0.0214, -0.2443), SIGLEC1 (down, 0.0113, -0.2555)
MSTRG.7219.1	up	0.0134	1.592	LOC108633201 (up, 0.0009, 0.3354), TNFRSF18 (up, 0.0027, 0.3213), KY (up, 0.0109, 0.2881), Capra_hircus_newGene_1504 (up, 0.0118, 0.2767), LOC102176870 (up, 0.0088, 0.2564), APLN (up, 0.0178, 0.2470), TNFSF4 (up, 0.0257, 0.2385), HS6ST3 (up, 0.0216, 0.2028), C1D (up, 0.0062, 0.1999), AMZ2 (up, 0.0447, 0.1973), TMEM123 (up, 0.0093, 0.1929), ASMTL (down, 0.0037, -0.2855)
MSTRG.7420.8	down	0.0342	-2.899	ADIPOR2 (down, 0.0441, -0.1419), SLC9A3R2 (down, 0.0483, -0.1615), PTRF (down, 0.0451, -0.1989), BICDL2 (down, 0.0357, -0.2026), RIMS3 (down, 0.0428, -0.2172), RAB3IL1 (down, 0.0492, -0.2194), C1QC (down, 0.041, -0.2304), FBLN5 (down, 0.0366, -0.2349)
MSTRG.7420.9	down	0.0231	-2.856	CCDC22 (down, 0.0389, -0.1870), Capra_hircus_newGene_1952 (up, 0.0029, 0.2627), C25H16orf59 (up, 0.0398, 0.2287), PNPLA6 (down, 0.0213, -0.1405), YPEL1 (down, 0.0203, -0.2455), PLAC9 (down, 0.0092, -0.2905), CLMN (down, 0.0039, -0.3259)
MSTRG.7687.1	down	0.0381	-1.561	Capra_hircus_newGene_11916 (down, 0.035, -0.1415), KIF13A (down, 0.0131, -0.1706), ATP8B2 (down, 0.0088, -0.1796), MN1 (down, 0.0145, -0.2759)
MSTRG.7691.16	up	0.0058	2.385	BIRC5 (up, 0.0088, 0.2550), PNPT1 (up, 0.0173, 0.1880), MARCH7 (up, 0.0025, 0.1770), RALGPS2 (up, 0.0216, 0.1380), SPAG1 (up, 0.003, 0.2879), NME7 (up, 0.0047, 0.2694), TSR3 (up, 0.0188, 0.2326), CDC40 (up, 0.0031, 0.2084), C1D (up, 0.0062, 0.1999), METTL17 (up, 0.0256, 0.1940), CXADR (up, 0.009, 0.1649), LDAH (up, 0.0313, 0.1579), THSD7A (down, 0.0294, -0.2382), RHOB (down, 0.0039, -0.3015)
MSTRG.7811.1	down	0.0267	-1.476	CNR1 (down, 0.0117, -0.2250), SLC35G1 (up, 0.0253, 0.2343), E2F3 (up, 0.0218, 0.1952), LOC102185150 (down, 0.0323, -0.1983), TRPM6 (down, 0.044, -0.2277), PRUNE2 (down, 0.025, -0.2336), Capra_hircus_newGene_2969 (down, 0.0169, -0.2698), RYR2 (down, 0.0101, -0.2808)
MSTRG.810.1	down	0.0140	-2.609	LOC102168573 (up, 0.0023, 0.2800), RALGPS2 (up, 0.0216, 0.1380), KRT7 (up, 0.0017, 0.3227), LOC102174170 (up, 0.0065, 0.3036), DTD2 (up, 0.0089, 0.2513), ZNF165 (up, 0.0284, 0.2438), LOC102179192 (up,

DE ncRNA	Regu lated	P value	log2Fold Change	Target mRNA (Regulated, P value, log2Fold Change)
				0.0383, 0.2335), MARCKSL1 (up, 0.0218, 0.1985), RET (up, 0.0156, 0.1862)
MSTRG.828.2	down	0.0296	-1.729	LOC102185150 (down, 0.0323, -0.1983)
MSTRG.8373.13	up	0.0196	1.513	MARCH7 (up, 0.0025, 0.1770), POU2F1 (up, 0.0325, 0.1270), TMEM221 (up, 0.0000, 0.3318), KRT7 (up, 0.0017, 0.3227), GLI1 (up, 0.0045, 0.2678), PRRG4 (up, 0.0178, 0.2181), LOC102169288 (up, 0.03, 0.2069), C1D (up, 0.0062, 0.1999), RPE (up, 0.0204, 0.1995), PPP1R7 (down, 0.0455, -0.1478), SP2 (down, 0.0328, -0.1892), AIP (down, 0.0216, -0.1957), PITX2 (down, 0.0453, -0.1976), CD247 (down, 0.0151, -0.2586)
MSTRG.8542.1	up	0.0032	2.783	RBM3 (up, 0.0002, 0.3306), Capra_hircus_newGene_11982 (up, 0.0073, 0.3031), Capra_hircus_newGene_12726 (up, 0.0159, 0.2591), PLEKHH2 (up, 0.0076, 0.2063), TRMT13 (up, 0.0348, 0.1971), LOC106503943 (up, 0.0485, 0.1846), ASB3 (up, 0.0331, 0.1443), LOC102179921 (down, 0.0066, -0.2763)
MSTRG.8822.1	up	0.0314	2.338	Capra_hircus_newGene_9422 (up, 0.0058, 0.3034), MAT1A (up, 0.0042, 0.2817), LOC106503901 (up, 0.0144, 0.2708), LOC106503969 (up, 0.0224, 0.2105), CEP89 (down, 0.0356, -0.2204)
MSTRG.8823.1	up	0.0302	1.734	TNFRSF18 (up, 0.0027, 0.3213), MAT1A (up, 0.0042, 0.2817)
MSTRG.8900.1	up	0.0000	10.153	WRN (up, 0.0188, 0.1520), ATP13A4 (up, 0.0000, 0.5377), TNFSF4 (up, 0.0257, 0.2385), FASTKD2 (up, 0.0031, 0.2242), CDC40 (up, 0.0031, 0.2084), C1D (up, 0.0062, 0.1999), DCK (up, 0.034, 0.1895), CXADR (up, 0.009, 0.1649), PPP1R7 (down, 0.0455, -0.1478), STMN3 (down, 0.0379, -0.1576), SOX4 (down, 0.0000, -1.1673)
MSTRG.8905.1	down	0.0153	-2.130	SLC39A3 (down, 0.0162, -0.2300), Capra_hircus_newGene_2167 (up, 0.0015, 0.3595), Capra_hircus_newGene_21365 (up, 0.0024, 0.3424), Capra_hircus_newGene_5074 (up, 0.0067, 0.3016), KCND2 (up, 0.0141, 0.2751), LOC102191766 (up, 0.0171, 0.2697), OPA1 (up, 0.0123, 0.1729), ARHGEF1 (down, 0.041, -0.1163), DCTN2 (down, 0.0161, -0.1403), PPP1R7 (down, 0.0455, -0.1478), ZBTB47 (down, 0.0011, -0.2435), CD247 (down, 0.0151, -0.2586), PLAC9 (down, 0.0092, -0.2905), GAS1 (down, 0.0002, -0.3759), MRGPRF (down, 0.0001, -0.4224), AADACL3 (down, 0.0000, -0.4595)
MSTRG.9044.10	down	0.0109	-3.940	LOC102177708 (down, 0.0034, -0.3300), FAM169B (down, 0.047, -0.2005)
MSTRG.9044.13	down	0.0493	-1.485	LOC102185708 (down, 0.0452, -0.1771), ATP8B2 (down, 0.0088, -0.1796), TEF (down, 0.0243, -0.1869), RBPMS2 (down, 0.0458, -0.2247), GPR153 (down, 0.0185, -0.2531), DDAH2 (down, 0.0099, -0.2543), CORIN (down, 0.01, -0.2553)

DE ncRNA	Regu lated	P value	log2Fold Change	Target mRNA (Regulated, P value, log2Fold Change)
MSTRG.9053.25	down	0.0001	-3.730	PMM1 (down, 0.0377, -0.2179)
MSTRG.9138.1	down	0.0484	-1.686	LOC102188618 (up, 0.0315, 0.2370), SRXN1 (up, 0.0278, 0.2185), STMN3 (down, 0.0379, -0.1576), BSCL2 (down, 0.0406, -0.1864), CAMK4 (down, 0.0332, -0.2359), TMEM35A (down, 0.0306, -0.2444)
MSTRG.9292.1	down	0.0001	-4.338	KRT36 (down, 0.0001, -0.4450), SLC39A3 (down, 0.0162, -0.2300), Capra_hircus_newGene_1952 (up, 0.0029, 0.2627), FGD6 (up, 0.0034, 0.2506), Capra_hircus_newGene_17456 (up, 0.0337, 0.2401), FASTKD2 (up, 0.0031, 0.2242), CXADR (up, 0.009, 0.1649), PNPLA6 (down, 0.0213, -0.1405), TSC1 (down, 0.0098, -0.1441), PPP1R7 (down, 0.0455, -0.1478), RUBCN (down, 0.0015, -0.1962), CAMK4 (down, 0.0332, -0.2359), ZBTB47 (down, 0.0011, -0.2435), YPEL1 (down, 0.0203, -0.2455), PLAC9 (down, 0.0092, -0.2905), PRKCDBP (down, 0.0011, -0.3262), SEPTIN6 (down, 0.0016, -0.3371), GAS1 (down, 0.0002, -0.3759), MRGPRF (down, 0.0001, -0.4224), SOX4 (down, 0.0000, -1.1673)
MSTRG.9308.35	down	0.0000	-9.179	ATP13A4 (up, 0.0000, 0.5377), KLHL29 (up, 0.0013, 0.2747), ZNF165 (up, 0.0284, 0.2438), LOC102182782 (up, 0.0312, 0.2428), E2F3 (up, 0.0218, 0.1952), CCDC50 (up, 0.0212, 0.1801), PITX2 (down, 0.0453, -0.1976), SNRPN (down, 0.016, -0.1981), SIDT2 (down, 0.0011, -0.2037), LOC106503362 (down, 0.0208, -0.2331), RFX2 (down, 0.0003, -0.4097)
MSTRG.9366.2	down	0.0114	-1.593	ZNF605 (up, 0.0085, 0.2370), CANT1 (down, 0.0341, -0.1450), SSR2 (down, 0.035, -0.1160), Capra_hircus_newGene_2167 (up, 0.0015, 0.3595), Capra_hircus_newGene_5074 (up, 0.0067, 0.3016), OPA1 (up, 0.0123, 0.1729), CAPNS1 (down, 0.0452, -0.1094), BCAP31 (down, 0.0218, -0.1273), ADIPOR2 (down, 0.0441, -0.1419), KLHL21 (down, 0.0117, -0.1713), GAS7 (down, 0.009, -0.1735), BICDL2 (down, 0.0357, -0.2026), RIMS3 (down, 0.0428, -0.2172), ZBTB47 (down, 0.0011, -0.2435), SEPN1 (down, 0.0023, -0.2478), GAS1 (down, 0.0002, -0.3759), MRGPRF (down, 0.0001, -0.4224)
MSTRG.9514.1	down	0.0316	-2.570	DIO2 (up, 0.0195, 0.2441), METTL17 (up, 0.0256, 0.1940), MCC (up, 0.0197, 0.1551), LOC102174081 (down, 0.0163, -0.2513)
MSTRG.9716.1	up	0.0064	2.245	LOC102168573 (up, 0.0023, 0.2800), Capra_hircus_newGene_11982 (up, 0.0073, 0.3031), C8H9orf72 (up, 0.0236, 0.1854), ZBTB6 (down, 0.0223, -0.1686), PRKD1 (down, 0.0013, -0.3107)
NC_030813.1:7252196|7268635	up	0.0134	5.059	PRSS12 (up, 0.0286, 0.2448)
NC_030835.1:40072020|40072294	down	0.0359	-4.506	SIPA1L2 (down, 0.0443, -0.1743)

DE ncRNA	Regulated	P value	log2Fold Change	Target mRNA (Regulated, P value, log2Fold Change)
novel_miR_269	down	0.0418	-1.522	Capra_hircus_newGene_18403 (down, 0.0486, -0.2210), CABYR (up, 0.0000, 0.5116), Capra_hircus_newGene_13914 (up, 0.0000, 0.4721), Capra_hircus_newGene_18572 (up, 0.0001, 0.4124), Capra_hircus_newGene_2167 (up, 0.0015, 0.3595), Capra_hircus_newGene_21230 (up, 0.0007, 0.3532), Capra_hircus_newGene_11982 (up, 0.0073, 0.3031), Capra_hircus_newGene_22304 (up, 0.0097, 0.2914), Capra_hircus_newGene_5523 (up, 0.0105, 0.2529), DIO2 (up, 0.0195, 0.2441), LOC102175781 (up, 0.0338, 0.2392), Capra_hircus_newGene_2169 (up, 0.0211, 0.2286), Capra_hircus_newGene_15790 (up, 0.0496, 0.2215), Capra_hircus_newGene_5067 (down, 0.0193, -0.1314), ALG2 (down, 0.034, -0.1464), SIGLEC1 (down, 0.0113, -0.2555), Capra_hircus_newGene_3416 (down, 0.0015, -0.3259)
novel_miR_325	up	0.0075	3.400	ENPP4 (down, 0.0217, -0.2271)
novel_miR_351	down	0.0464	-1.967	Capra_hircus_newGene_17456 (up, 0.0337, 0.2401), Capra_hircus_newGene_15790 (up, 0.0496, 0.2215), HS6ST3 (up, 0.0216, 0.2028)
novel_miR_380	down	0.0458	-2.067	Capra_hircus_newGene_18572 (up, 0.0001, 0.4124), Capra_hircus_newGene_12245 (up, 0.0185, 0.2382), LRP2 (up, 0.0105, 0.2375), E2F3 (up, 0.0218, 0.1952), SIGLEC1 (down, 0.0113, -0.2555), MN1 (down, 0.0145, -0.2759)
novel_miR_390	down	0.0211	-1.949	Capra_hircus_newGene_18572 (up, 0.0001, 0.4124), Capra_hircus_newGene_2167 (up, 0.0015, 0.3595), Capra_hircus_newGene_22304 (up, 0.0097, 0.2914),
novel_miR_410	down	0.0172	-5.304	LRP2 (up, 0.0105, 0.2375), FZD8 (up, 0.0352, 0.1967), KLHDC8B (down, 0.0443, -0.2272), SIGLEC1 (down, 0.0113, -0.2555), SPTB (down, 0.0032, -0.2967), RFX2 (down, 0.0003, -0.4097)
novel_miR_420	down	0.0434	-4.016	CD40 (up, 0.0437, 0.2030), Capra_hircus_newGene_18572 (up, 0.0001, 0.4124), Capra_hircus_newGene_21230 (up, 0.0007, 0.3532), Capra_hircus_newGene_1504 (up, 0.0118, 0.2767), GLI1 (up, 0.0045, 0.2678), Capra_hircus_newGene_1952 (up, 0.0029, 0.2627), Capra_hircus_newGene_5523 (up, 0.0105, 0.2529), FGD6 (up, 0.0034, 0.2506), C14H8orf82 (up, 0.0302, 0.2351), Capra_hircus_newGene_2169 (up, 0.0211, 0.2286), Capra_hircus_newGene_15790 (up, 0.0496, 0.2215), Capra_hircus_newGene_11971 (up, 0.037, 0.2193), ALKBH5 (down, 0.0214, -0.1488), MAP3K3 (down, 0.0342, -0.1613), C2CD2L (down, 0.0163, -0.2549), Capra_hircus_newGene_3416 (down, 0.0015, -0.3259), DZIP1L (down, 0.0009, -0.3689)

DE ncRNA	Regulated	P value	log2Fold Change	Target mRNA (Regulated, P value, log2Fold Change)
novel_miR_421	down	0.0421	-2.845	TMTC1 (down, 0.0149, -0.1880), Capra_hircus_newGene_21230 (up, 0.0007, 0.3532), TMEM221 (up, 0.0000, 0.3318), Capra_hircus_newGene_22304 (up, 0.0097, 0.2914), Capra_hircus_newGene_1504 (up, 0.0118, 0.2767), Capra_hircus_newGene_9735 (up, 0.0153, 0.2735), PTPN21 (down, 0.0431, -0.1416), SNX33 (down, 0.0234, -0.1527), DYRK1B (down, 0.0187, -0.1879), CAMK4 (down, 0.0332, -0.2359), FAT4 (down, 0.0251, -0.2533), PRKCDBP (down, 0.0011, -0.3262)
novel_miR_59	down	0.0416	-1.556	ZNRF2 (up, 0.0382, 0.1620), Capra_hircus_newGene_18572 (up, 0.0001, 0.4124), Capra_hircus_newGene_12502 (up, 0.0035, 0.2992), CCDC50 (up, 0.0212, 0.1801)
novel_miR_662	up	0.0446	2.243	HSPA2 (down, 0.033, -0.2230), ZNRF2 (up, 0.0382, 0.1620), Capra_hircus_newGene_21365 (up, 0.0024, 0.3424), KRT7 (up, 0.0017, 0.3227), EPPK1 (up, 0.012, 0.2178), CCDC50 (up, 0.0212, 0.1801), MCC (up, 0.0197, 0.1551), TJP2 (down, 0.0315, -0.1445), FLNA (down, 0.0367, -0.1555), SLC15A4 (down, 0.0329, -0.1808), AKAP17A (down, 0.0305, -0.1924), LOC102182149 (down, 0.0116, -0.2094), LOC108634770 (down, 0.037, -0.2143), FZD2 (down, 0.0263, -0.2244), SOX4 (down, 0.0000, -1.1673)
novel_miR_723	up	0.0358	2.594	APLN (up, 0.0178, 0.2470), E2F3 (up, 0.0218, 0.1952), RASEF (up, 0.0469, 0.1949)
novel_miR_724	down	0.0500	-1.389	Capra_hircus_newGene_18403 (down, 0.0486, -0.2210), Capra_hircus_newGene_18572 (up, 0.0001, 0.4124), Capra_hircus_newGene_2167 (up, 0.0015, 0.3595), Capra_hircus_newGene_21230 (up, 0.0007, 0.3532), FMO4 (up, 0.006, 0.3088), Capra_hircus_newGene_5074 (up, 0.0067, 0.3016), Capra_hircus_newGene_12502 (up, 0.0035, 0.2992), Capra_hircus_newGene_22304 (up, 0.0097, 0.2914), Capra_hircus_newGene_12726 (up, 0.0159, 0.2591), Capra_hircus_newGene_5523 (up, 0.0105, 0.2529), PPAT (up, 0.0255, 0.2475), DIO2 (up, 0.0195, 0.2441), Capra_hircus_newGene_17456 (up, 0.0337, 0.2401), LOC102175781 (up, 0.0338, 0.2392), Capra_hircus_newGene_2169 (up, 0.0211, 0.2286), Capra_hircus_newGene_15790 (up, 0.0496, 0.2215), Capra_hircus_newGene_11971 (up, 0.037, 0.2193), Capra_hircus_newGene_19529 (up, 0.0029, 0.2146), DDX47 (up, 0.0314, 0.1675), LDAH (up, 0.0313, 0.1579), BLOC1S6 (up, 0.0432, 0.1478), CC2D1B (up, 0.0303, 0.1309), ALG2 (down, 0.034, -0.1464), ZBTB6 (down, 0.0223, -0.1686), GAS7 (down, 0.009, -0.1735), LOC102168757 (down, 0.0366, -0.2224), CAMK4 (down, 0.0332, -0.2359), CD247 (down, 0.0151, -0.2586), Capra_hircus_newGene_3416 (down, 0.0015, -0.3259)

DE ncRNA	Regulated	P value	log2Fold Change	Target mRNA (Regulated, P value, log2Fold Change)
novel_miR_726	down	0.0171	-5.304	LRP2 (up, 0.0105, 0.2375), FZD8 (up, 0.0352, 0.1967), KLHDC8B (down, 0.0443, -0.2272), SIGLEC1 (down, 0.0113, -0.2555), SPTB (down, 0.0032, -0.2967), RFX2 (down, 0.0003, -0.4097)
novel_miR_786	down	0.0228	-1.639	Capra_hircus_newGene_9735 (up, 0.0153, 0.2735), Capra_hircus_newGene_12245 (up, 0.0185, 0.2382), FLNC (down, 0.0403, -0.2226), Capra_hircus_newGene_19483 (down, 0.0094, -0.2813)
novel_miR_87	up	0.0219	2.610	FAM111B (up, 0.0007, 0.3640), ADAMTS15 (down, 0.0057, -0.3114), Capra_hircus_newGene_9136 (up, 0.0104, 0.2847), TBX1 (up, 0.0236, 0.2136), RP2 (down, 0.0474, -0.2078), MAP3K3 (down, 0.0342, -0.1613), CTU1 (down, 0.0105, -0.1511)
novel_miR_874	down	0.0122	-1.317	Capra_hircus_newGene_22856 (up, 0.0029, 0.3335), TPPP (down, 0.0373, -0.2333), LSAMP (down, 0.0286, -0.2352), ZBTB47 (down, 0.0011, -0.2435)
novel_miR_879	down	0.0171	-5.304	LRP2 (up, 0.0105, 0.2375), FZD8 (up, 0.0352, 0.1967), KLHDC8B (down, 0.0443, -0.2272), SIGLEC1 (down, 0.0113, -0.2555), SPTB (down, 0.0032, -0.2967), RFX2 (down, 0.0003, -0.4097)

Note: 0.000 indicates that the P value is less than 0.0001

Table S4. Summary of GO annotation of non-coding RNAs target genes associated with epidermal epithelial cells

DE lncRNA	Regulated of lncRNA	Target type	Targeted DE mRNA	Regulated of mRNA	GO term
MSTRG.10816.1	up	trans	*BCL9L*	down	positive regulation of epithelial to mesenchymal transition (GO:0010718)
MSTRG.40315.1	down	trans			
MSTRG.42054.1	up	trans			
MSTRG.23680.2	up	trans			
MSTRG.33425.1	up	trans			
MSTRG.14664.1	up	trans			
MSTRG.17227.2	up	trans			
MSTRG.27601.1	up	trans			
MSTRG.35724.1	up	trans			
MSTRG.13439.1	up	trans			
MSTRG.46907.1	down	trans	*CAV1*	down	negative regulation of epithelial cell differentiation (GO:0030857)
MSTRG.46161.2	down	trans			
MSTRG.35591.10	down	trans			
MSTRG.52657.1	down	trans			
MSTRG.23282.9	down	trans			
MSTRG.11813.4	up	trans	*COL5A1*	down	wound healing, spreading of Epidermal cells (GO:0035313)
MSTRG.28026.4	up	trans			

DE lncRNA	Regulated of lncRNA	Target type	Targeted DE mRNA	Regulated of mRNA	GO term
MSTRG.26322.32	up	trans			
MSTRG.2866.1	down	trans	*CXADR*	up	epithelial structure maintenance (GO:0010669)
MSTRG.18451.4	down	trans			
MSTRG.28095.1	up	trans			
MSTRG.19158.1	up	trans			
MSTRG.1312.2	up	trans			
MSTRG.36438.1	up	trans			
MSTRG.7691.16	up	trans			
MSTRG.13429.1	down	trans			
MSTRG.50083.1	up	trans			
MSTRG.40437.9	up	trans			
MSTRG.28754.1	down	trans			
MSTRG.43931.5	up	trans			
MSTRG.38981.1	down	trans			
MSTRG.40477.1	up	trans			
MSTRG.9292.1	down	trans			
MSTRG.24585.1	up	trans			
MSTRG.53873.4	up	trans			
MSTRG.58065.1	down	trans			
MSTRG.8900.1	up	trans			
MSTRG.4872.1	up	trans	*GLI1*	up	epidermal cell differentiation (GO:0009913)
MSTRG.44954.1	up	trans			
MSTRG.49210.1	down	trans			
MSTRG.11813.4	up	trans			
MSTRG.26212.1	up	trans			
MSTRG.51751.1	up	trans			
MSTRG.5971.1	up	trans			
MSTRG.47808.9	up	trans			
MSTRG.8373.13	up	trans			
MSTRG.37910.1	up	trans	*KLF4*	down	epidermal cell differentiation (GO:0009913), epidermis development (GO:0008544), epithelial cell differentiation (GO:0030855), epithelium development (GO:0060429), morphogenesis of an epithelium (GO:0002009), negative regulation of epithelial cell migration (GO:0010633), regulation of epithelial cell migration (GO:0010632)
MSTRG.37574.4	up	trans			
MSTRG.1312.2	up	trans	*MCC*	up	negative regulation of epithelial cell migration (GO:0010633), negative
MSTRG.6171.7	down	trans			

DE lncRNA	Regulated of lncRNA	Target type	Targeted DE mRNA	Regulated of mRNA	GO term
MSTRG.24679.39	up	cis			regulation of epithelial cell proliferation (GO:0050680)
MSTRG.53357.5	down	trans			
MSTRG.9514.1	down	trans			
MSTRG.18252.1	down	trans			
MSTRG.42054.1	up	trans	*MMP12*	up	positive regulation of epithelial cell proliferation involved in wound healing (GO:0060054)
MSTRG.23680.2	up	trans			
MSTRG.33425.1	up	trans			
MSTRG.25102.1	up	trans			
MSTRG.58272.19	up	trans			
MSTRG.57117.11	up	trans			
MSTRG.58695.9	up	trans			
MSTRG.56329.1	up	trans			
MSTRG.23593.17	up	trans			
MSTRG.22301.1	up	trans			
MSTRG.48456.9	up	trans			
MSTRG.40738.3	up	trans			
MSTRG.36911.4	up	trans			
MSTRG.13409.8	up	trans			
MSTRG.54591.4	up	trans			
MSTRG.520.1	up	trans			
MSTRG.22083.13	up	trans			
MSTRG.6557.1	down	trans	*NCSTN*	down	epithelial cell proliferation (GO:0050673)
MSTRG.21713.12	down	trans			
MSTRG.55802.1	down	trans			
MSTRG.18602.3	down	trans			
MSTRG.27029.1	down	trans			
MSTRG.49210.1	down	trans	*PTCH2*	up	positive regulation of Epidermal cell differentiation (GO:0045606)
MSTRG.2199.13	down	trans			
MSTRG.34920.1	up	trans			
MSTRG.25997.1	up	trans			
MSTRG.55802.1	down	trans	*TGFB1I1*	down	epithelial cell differentiation (GO:0030855), positive regulation of epithelial to mesenchymal transition (GO:0010718), morphogenesis of embryonic epithelium (GO:0016331)
MSTRG.18602.3	down	trans			
MSTRG.34920.1	up	trans	*WNT5A*	up	establishment of epithelial cell apical/basal polarity (GO:0045198), mesenchymal-epithelial cell signaling (GO:0060638), negative regulation of epithelial cell proliferation (GO:0050680)

Table S5. KEGG annotation results of non-coding RNA target gene

Non-coding RNA	Target genes	KEGG pathway
MSTRG.28870.6	*CASP3*	MAPK signaling pathway (ko04019)
MSTRG.10816.1		
MSTRG.48174.1		
MSTRG.40315.1		
MSTRG.12102.1		
MSTRG.33425.1		
novel_miR_662	*FLNA*	MAPK signaling pathway (ko04019)
MSTRG.53655.1	*FLNC*	MAPK signaling pathway (ko04019)
novel_miR_786		
MSTRG.40315.1	*FZD2*	Wnt signaling pathway (ko04310); mTOR signaling pathway (ko04153)
novel_miR_662		
MSTRG.46477.1	*FZD8*	Wnt signaling pathway (ko04310); mTOR signaling pathway (ko04152)
MSTRG.55880.4		
novel_miR_410		
novel_miR_726		
novel_miR_879		
MSTRG.42720.20	*HSPA2*	MAPK signaling pathway (ko04020)
novel_miR_662		
MSTRG.4872.1	*ITGA2*	PI3K-Akt signaling pathway (ko04155)
MSTRG.265.11		
MSTRG.10297.1		
MSTRG.56717.1		
MSTRG.28656.4	*MAP3K3*	MAPK signaling pathway (ko04015)
novel_miR_420		
novel_miR_87		
MSTRG.6557.1	*NCSTN*	Notch signaling pathway (ko04330)
MSTRG.44954.1	*PIK3CB*	PI3K-Akt signaling pathway (ko04151); mTOR signaling pathway (ko04150)
MSTRG.49210.1		
MSTRG.30256.4	*PPP2R2B*	PI3K-Akt signaling pathway (ko04153)
MSTRG.45196.1	*RALGPS2*	MAPK signaling pathway (ko04012)
MSTRG.7691.16		
MSTRG.28870.6	*RASA1*	MAPK signaling pathway (ko04016)
MSTRG.10816.1		
MSTRG.28095.1	*STMN3*	MAPK signaling pathway (ko04011)
MSTRG.50548.9		
MSTRG.6630.1		
MSTRG.19158.1		
MSTRG.4315.36		
MSTRG.43654.7		
MSTRG.49414.1		

Non-coding RNA	Target genes	KEGG pathway
MSTRG.48174.1	*THBS2*	PI3K-Akt signaling pathway (ko04154)
MSTRG.1312.2	*THSD7A*	Wnt signaling pathway (ko04310)
MSTRG.46598.1		
MSTRG.6171.7		
MSTRG.7691.16		
MSTRG.16128.1	*TSC1*	PI3K-Akt signaling pathway (ko04156); mTOR signaling pathway (ko04154)
MSTRG.51287.1		

Table S6. Primer information of lncRNA

Gene Name	Sequence（5'- 3'）	product Length (bp)	ID/ Location
MSTRG.2199.13	F: GCTCAGGAAGCACTCACTAGG<br>R: GAGGAGGGTCTCTGGCATTC	140	NC_030808.1<br>130619220-130621649
MSTRG.35284.4	F: TGTGTGTTGTCACGAGTGGAA<br>R: TGGGCACAACTTACAGGAACA	178	NC_030823.1<br>58100875-58121435
MSTRG.46161.2	F: TCCAAGCTATGGCGTGTGTT<br>R: GAAAGCAACTTGAGGTCGCC	115	NC_030829.1<br>59172504-59176799
MSTRG.4208.4	F: TGTCCTCAGTATCCAGCCAGA<br>R: AGCTGCTCCTCCTGCTAAAT	196	NC_030809.1<br>50085278-50169981
MSTRG.58766.1	F: GCCTTTCTCTGTGCCCACTC<br>R: CCCTGTAATCTTGGCTCGGTT	110	NW_017216215.1<br>10700-12378
MSTRG.24220.5	F: GAACACCGCAGGAACTGGAA<br>R: AGTTTCCTTGCTGTCACCCG	118	NC_030817.1<br>83633965-83642096
MSTRG.8900.1	F: TGCCACCCATTGTAGGTTCC<br>R:AGAGCCCACTGAACCCTTTG	198	NC_030811.1<br>18106103-18127046
MSTRG.40421.14	F: TCAGGCACCAGTAGGGTAGAA<br>R: ATCAGCAAGCCAGTGGTACT	196	NC_030826.1<br>33362427-33372026
GAPDH	F: GGCGTGAACCACGAGAAGTAT<br>R: ATGGCGTGGACAGTGGTCAT	143	XM_005680968.3
